# The Crowded Uterine Horn Mouse Model for Examining Postnatal Metabolic Consequences of Intrauterine Growth Restriction vs. Macrosomia in Siblings

**DOI:** 10.3390/metabo12020102

**Published:** 2022-01-22

**Authors:** Julia A. Taylor, Benjamin L. Coe, Toshi Shioda, Frederick S. vom Saal

**Affiliations:** 1 Division of Biological Sciences, University of Missouri-Columbia, Columbia, MO 65211, USA; coeb@health.missouri.edu (B.L.C.); vomsaalf@missouri.edu (F.S.v.S.); 2 Center for Cancer Research, Massachusetts General Hospital and Harvard Medical School, Building 149, 13th Street, Charlestown, MA 02129, USA; shioda@helix.mgh.harvard.edu

**Keywords:** intrauterine growth restriction, macrosomia, glucose tolerance, abdominal adipocyte gene expression, thrifty phenotype hypothesis

## Abstract

Differential placental blood flow and nutrient transport can lead to both intrauterine growth restriction (IUGR) and macrosomia. Both conditions can lead to adult obesity and other conditions clustered as metabolic syndrome. We previously showed that pregnant hemi-ovariectomized mice have a crowded uterine horn, resulting in siblings whose birth weights differ by over 100% due to differential blood flow based on uterine position. We used this crowded uterus model to compare IUGR and macrosomic male mice and also identified IUGR males with rapid (IUGR-R) and low (IUGR-L) postweaning weight gain. At week 12 IUGR-R males were heavier than IUGR-L males and did not differ from macrosomic males. Rapid growth in IUGR-R males led to glucose intolerance compared to IUGR-L males and down-regulation of adipocyte signaling pathways for fat digestion and absorption and type II diabetes. Macrosomia led to increased fat mass and altered adipocyte size distribution compared to IUGR males, and down-regulation of signaling pathways for carbohydrate and fat digestion and absorption relative to IUGR-R. Clustering analysis of gonadal fat transcriptomes indicated more similarities than differences between IUGR-R and macrosomic males compared to IUGR-L males. Our findings suggest two pathways to adult metabolic disease: macrosomia and IUGR with rapid postweaning growth rate.

## 1. Introduction

In the United States and many developed countries, the incidence of obesity and related diseases, collectively referred to as metabolic syndrome, are increasing at a rapid rate [1]. Evidence from epidemiological studies links the rate of fetal growth, body weight at birth, rate of growth during early postnatal life, and adult metabolic diseases [2]. Namely, when there is reduced fetal growth and body weight at birth, but the subsequent postnatal growth rate is markedly higher than the median, this results in body weight centile crossing, and significant metabolic abnormalities occur. This sequence of events is a central feature of the developmental basis of health and disease (DOHaD) hypothesis for metabolic diseases [3].

Many studies have investigated the effects of maternal nutrition during pregnancy. Decreased maternal nutrition during pregnancy leads to intrauterine growth-restriction (IUGR), and the small for gestational age babies in the bottom 10th percentile for birth weight are at increased risk of being overweight in adulthood [4,5]. Similarly, babies that are in the top fifth percentile for body weight at birth (macrosomic), a condition often associated with maternal obesity and/or diabetes, are also at risk for adult obesity [6]. Regardless of the basis for their obesity, the outcome is that most obese individuals are at increased risk for developing other disorders associated with metabolic syndrome. Among these co-morbidities are increased blood pressure, cardiovascular disease, insulin insensitivity, glucose intolerance, diabetes, fatty liver disease, and elevated triglycerides and cholesterol [1,2,7].

A large number of animal studies investigating these conditions have manipulated maternal nutrition to induce IUGR or macrosomia in the offspring [8,9,10]. These models alter fetal growth trajectories via maternal protein restriction, caloric restriction, use of a feed with a very high percentage of fat, or streptozocin-induced type 1 diabetes mellitus to increase fetal glucose uptake. However, although maternal malnutrition is a cause of IUGR in non-developed countries, war zones, and in cases of eating disorders [4,11], dietary restriction paradigms do not adequately represent the human condition associated with IUGR in developed countries, where the cause of IUGR is not typically severe caloric or protein restriction.

Many factors can contribute to reduced fetal nutrition without a decrease in maternal nutrition, such as insufficient blood flow to the placenta or deficits in placental transport of specific nutrients [12]. Another approach to creating fetal undernutrition has been to experimentally create insufficient transport of nutrients to fetuses from the maternal circulation as a result of ligation of the uterine blood vessels [13]. However, a problem with this approach is that there are physiological responses to trauma in addition to surgically restricting blood flow to specific placentae [14].

While it was once not uncommon for parents to be advised to over-feed IUGR babies, there is now extensive epidemiological evidence showing that IUGR babies who experience a rapid “catch-up” growth spurt during infancy or early childhood are at high risk for adult obesity, type 2 diabetes and other co-morbidities of metabolic syndrome, consistent with the “thrifty phenotype” hypothesis [15]. The hypothesis is that the physiological “program” of IUGR babies is one that makes them adapted for a lifetime of reduced nutrition, and these babies are thus at high risk for becoming overweight when they are exposed to a typical highly processed Western diet that is higher in calories than their physiological program’s “set point” [16,17]. Thus, in humans, fetal growth rate interacts with the growth rate during early postnatal life to determine whether IUGR leads to adult obesity and other metabolic diseases.

We examined here the consequences for male mice of developing in a crowded uterine horn that results in differential placental blood flow based on the random implantation of fetuses in the middle vs. either end of the uterine horn. This is an IUGR and macrosomia model that results in animals that match the clinical description of IUGR in the bottom 10th percentile for human fetuses and of macrosomia at the other end of the birth weight spectrum [18].

**Figure 1:**
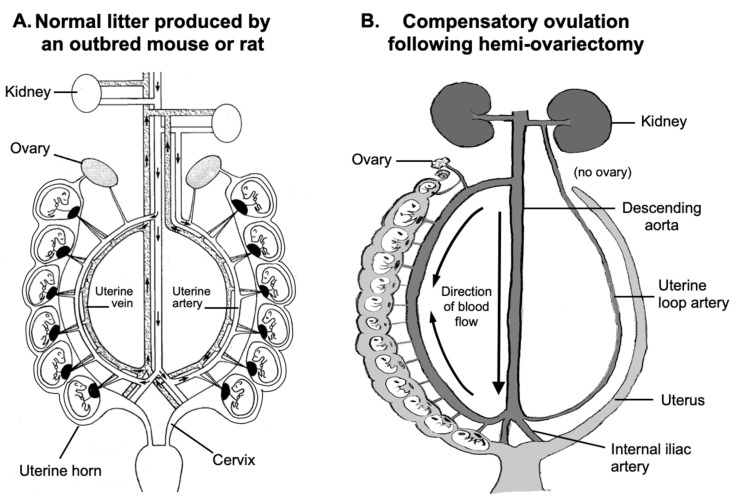
The pregnant mouse uterus. (**A**) The intact duplex uterus of the mouse or rat, with each uterine horn having an independent cervix. The bi-direction blood flow in each uterine artery and vein is indicated by arrows. Modified from Even et al. [21], with permission. The left kidney is caudal to the right kidney, which leads to variability in the cranial vascular anatomy of the left uterine horn. (**B**) The consequence of hemi-ovariectomy on ovulation in litter-bearing species that normally ovulate from both ovaries, which is referred to as compensatory ovarian hypertrophy due to the remaining ovary ovulating the normal number of oocytes that would have been produced by both ovaries. Due to the variability in the vascular anatomy of the left uterine horn, all mice had the left ovary removed, which resulted in compensatory ovulation by the remaining right ovary and crowding of fetuses in the right uterine horn. Fetuses that end up randomly implanted in the middle portion of the uterus [24] have reduced blood flow and nutrient transport across the placenta relative to siblings at the ends of the horn, due to the bi-directional uterine arterial blood flow; however, if the fetus at the cranial end of the crowded uterus has a placental artery that branches off of the ovarian artery (as shown in Panel **B**), this fetus will be IUGR rather than macrosomic [19,22]. Modified from Coe et al. [22], with permission.

In more detail, previous studies in mice suggested that crowding a uterus with more fetuses than is normal resulted in the production of offspring that varied in the rate of fetal growth as a result of differences in the amount of placental blood flow [19]. In a series of studies, we demonstrated [20,21] that blood flows from two directions into the artery supplying each independent uterine horn in rats and mice, which have a duplex uterus. Specifically, blood flows into each uterine artery from both the cranial end, branching off of the descending aorta, and the caudal end, which branches off of the ipsilateral iliac artery (Figure 1). The unusual “loop” vascular structure results in a greater flow of blood to the placentae located at the cranial and caudal ends of each uterine horn relative to placentae located in the middle of each horn in both rats and mice. The magnitude of the effect on blood flow to each placenta and on fetal growth as a result of being positioned at the ends or middle of a uterine horn in mice is greatly exaggerated if one ovary is removed since a change in follicular dynamics in the remaining ovary causes hyper-ovulation, referred to as compensatory ovarian hypertrophy [19,22,23]. A mouse that becomes pregnant after hemi-ovariectomy will thus have a crowded uterine horn since embryos cannot migrate from one uterine horn to the other in mice. This crowded uterine horn results in dramatic differences between siblings in fetal nutrient availability based on implantation site, which is a random event [24].

In the present study, we used the crowded uterus phenomenon as a model system to increase differences between siblings in the rate of fetal growth and thus body weight at birth. This allowed us to examine the consequences for postnatal growth rate, and for adipocyte number, size and gene expression in the largest abdominal fat pad, which in mice is associated with the gonads. We also weighed the other major fat pads in mice: the abdominal renal fat pads and the subcutaneous inguinal fat pads. In addition, we examined glucose tolerance in male mice that were classified as IUGR (<10% of birth weight range), median (~50%) or macrosomic (>93%) at birth due to differential placental blood flow based purely on their random position within the uterus.

We report here results from the crowded uterus model, which provides the opportunity to examine the etiology of differences caused by differential fetal and postnatal growth without nutrient or surgical intervention. Our hypothesis was that IUGR males that experienced a rapid period of postnatal growth (referred to as IUGR-R males) would in adulthood appear similar to males identified as macrosomic at birth. However, we expected both IUGR-R and macrosomic males to show significant differences from IUGR males that exhibited a low rate of postnatal growth (referred to as IUGR-L males). We identified significant differences in adult phenotype between IUGR male mice based on their rate of growth (low vs. rapid), providing support in this animal model for the “thrifty phenotype” hypothesis. Interestingly, there were also differences in phenotype between IUGR-R males and macrosomic males, suggesting that these two pathways to adult metabolic diseases need to be considered when seeking approaches to mitigate the metabolic abnormalities of these individuals.

## 2. Results

### 2.1. Placental Blood Flow and Fetal Growth

The placement of fetuses in the uterus impacts fetal growth [19,22]. Here we chose to only examine offspring that developed in one crowded uterine horn. A schematic of a normal pregnancy is shown in Figure 1A, and a crowded uterine horn is shown in Figure 1B.

### 2.2. Birth Weight Criteria and Postnatal Growth Rate

**Figure 2:**
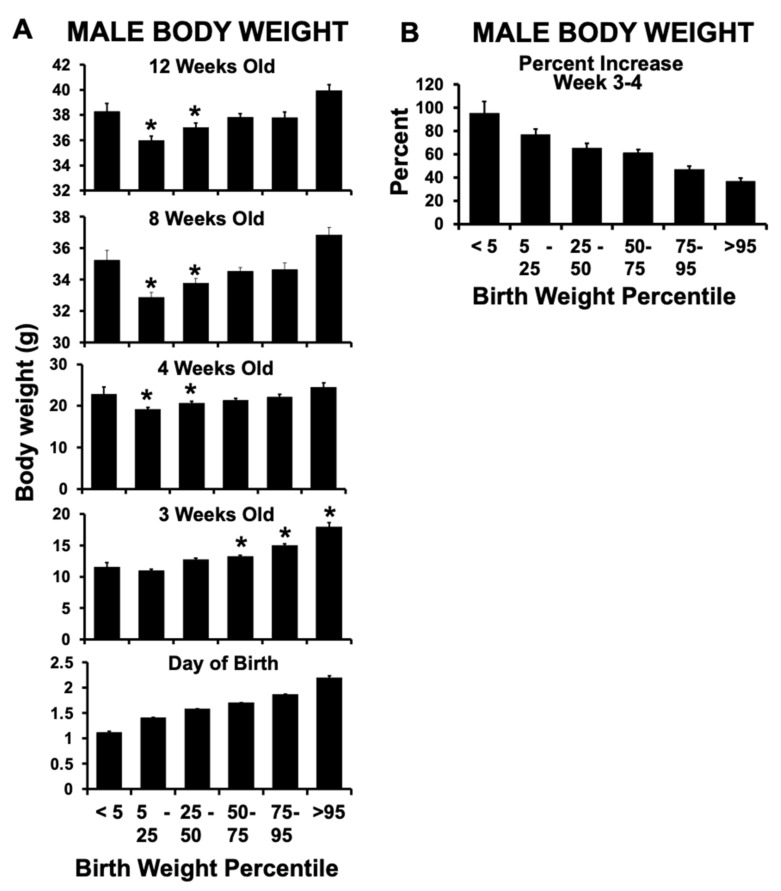
Body weight and growth of Block 1 male mice. (**A**) Body weights of male mice produced in 52 litters by Block 1 hemi-ovariectomized females based on birth weight categories, from the ≤5th percentile to ≥95th percentile. Beginning at postnatal week 3 (weaning), body weights were measured for all surviving male offspring until postnatal week 12. (**B**) the percent increase in body weight for these males during the first week of free-feeding after weaning (week 3–4). A subset of these Block 1 males (from IUGR, median and macrosomic birth weight categories) were retained after week 12 for additional studies. (See Table S1). * *p* < 0.05 compared to males from the bottom 5th percentile (IUGR males). Values are mean ± SEM.

CD-1 mouse litters were produced in two blocks. In Block 1, 52 litters were produced by hemi-ovariectomized postpartum females resulting in a total of 605 offspring, of which 585 survived until weaning. The overall pre-weaning mortality rate was 3.3%, with 25 percent of all pre-weaning deaths occurring in IUGR animals, and only 0.05% in macrosomic animals. The total number of animals at birth consisted of 297 females and 308 males; the distribution of all male offspring body weights from birth until 12 weeks old is presented in Figure 2A; body weight at birth data were normally distributed. The mean weight for all male mice on the day of birth was 1.64 ± 0.24 g (mean ± SEM).

Of the animals that survived until weaning, the weight range for males with birth weights in the bottom 5th percentile (designated as IUGR; *n* = 14) was 0.95–1.23 g (mean ± SEM: 1.12 ± 0.03 g) (Figure 2A). Males categorized as being at the “median” (~1.64 g) body weight range were in the 47.4–51.2 percentile of all birth weights (*n* = 16 saved for follow-up experiments). The weight range for males with birth weights in the top 5th percentile (designated as macrosomic) was 2.02–2.40 g (*n* = 15); the mean (±SEM) was 2.20 ± 0.04 g.

Body weight between birth and weaning (week 3) increased as a function of body weight at birth (Figure 2A). However, the percent weight gain in the first week after weaning (between weeks 3–4) showed a dramatic decrease as a function of body weight category at birth (Figure 2B). Namely, the IUGR males (bottom 5th percentile) exhibited a 95% increase in their body weight between week 3 (weaning) and week 4 of life, while macrosomic males (top 95th percentile) showed only a 37% increase during the same time. Thus, by week 4, the IUGR males had reached a body weight that was not significantly different from males identified as macrosomic at birth. In contrast, the males in the 5–50% body weight at birth categories were significantly lighter than IUGR males by postnatal week 4 and into adulthood, with the last measurement being made when all males were 12 weeks old (Figure 2A).

### 2.3. Different Sub-Groups of IUGR Males

**Figure 3:**
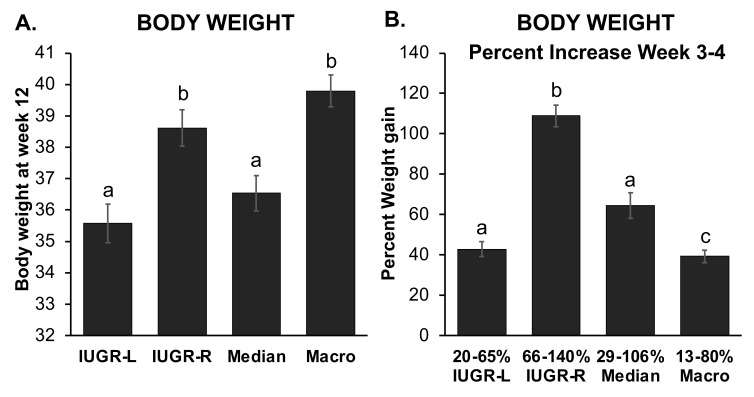
Body weight and percent growth of IUGR-L (*n* = 13), IUGR-R (*n* = 16), median (*n* = 17) and macrosomic (*n* = 21) males. (**A**) body weights at week 12, when IUGR-L and median males weighed less than IUGR-R and macrosomic males. (**B**) the percent postweaning (week 3–4) weight gain, showing that IUGR-R males exhibited the most rapid post-weaning weight gain, while macrosomic males showed the lowest week 3–4 percent weight gain. Groups with different letters above the error bar are significantly different from each other; groups that share a letter in common are not statistically different. Values are mean ± SEM.

A sub-group of the IUGR males did not go through a rapid post-weaning growth phase, although in Figure 2 they are included in the mean growth rate for all IUGR males. Of the animals that were below the 10th percentile for birth weight, 45% (*n* = 13/29) did not experience a post-weaning rapid weight gain. These males, who gained less than 65% of their weight during weeks 3–4, referred to here as IUGR-low postweaning growth rate (IUGR-L) males, were compared with IUGR-rapid post-weaning growth rate males (IUGR-R) that gained over 65% body weight during this period. IUGR-L males achieved a lower adult body weight by week 12 relative to IUGR-R or macrosomic males, while IUGR-L did not differ from median body weight at birth males (Figure 3A). The percent weight gain between weeks 3–4 is shown in Figure 3B. The IUGR-R males had a lower birth weight than IUGR-L males (1.166 ± 0.030 g vs.1.253 ± 0.021 g, *p* = 0.029) and were also in a lower birth weight percentile than IUGR-L males (3.925 ± 0.618% vs. 6.248 ± 0.748%, *p* = 0.023).

Within the macrosomic group of males, the range of body weight gain during the week after weaning was smaller, and there was no significant difference in adult body weight based on the rate of growth during the week after weaning (data not shown).

### 2.4. Experiments on Block 1 and Block 2 Animals When 6 Months Old

After week 12, selected IUGR, median and macrosomic males from Block 1, here defined as below the 10th percentile at birth, at the median, and above the 93rd percentile respectively, were saved for further studies. IUGR males were subdivided into IUGR-L and IUGR-R animals based on differential growth rate during postnatal weeks 3–4, as described above. Block 1 males included the first set of animals that were subjected to a 14-h fast prior to a glucose tolerance test (GTT) and a second set that did not experience either a fast or a GTT (Sets 1 and 2, respectively, see Table S1).

We generated a second cohort of animals (Block 2) from an additional 21 hemi-ovariectomized females (see Table S1), IUGR, median and macrosomic at birth animals were identified using the same criteria as for Block 1. IUGR males were again separated into rapid- and slow-growing based on the differential postweaning growth rate, although for Block 2 we selected a slightly higher cutoff, at less than or above 76% postweaning weight gain.

We conducted experiments on males from the three different birth weight percentiles (IUGR, median and macrosomic), as well as differing post-weaning growth percentiles for IUGR animals (IUGR-L and IUGR-R) when they were six months old.

First, we conducted a glucose tolerance test (GTT) on selected males (Set 1 in Table S1) from Block 1. IUGR-L, IUGR-R, median and macrosomic males were fasted for 14 h overnight (during the dark phase of the light:dark cycle prior to the GTT and were sacrificed immediately after the GTT; organ weights were measured, and the fat pads were collected for analysis of gonadal adipocyte cell number and size, and analysis of expression of selected genes by quantitative reverse transcription polymerase chain reaction (qPCR) in gonadal fat.

Second, we also conducted a GTT on the Block 2 males. These animals were only fasted during the first 4 h of the light phase, for comparison with the effects of the overnight 14-h fast, and were not sacrificed for at least 1–2 weeks post-test. Body weights and gonadal fat pad weights were also measured, but no further analyses were conducted.

Finally, to rule out any effect of fasting associated with the GTT on fat, we also collected fat from the second set of males from Block 1 (Set 2 in Table S1), without fasting the animals or conducting a GTT prior to collection of fat. We collected gonadal, renal and inguinal fat pads from these males, and then conducted an analysis of gonadal adipocyte number and size, as well as analysis of gonadal fat gene expression by both qPCR and microarray.

### 2.5. Glucose Tolerance Test (GTT)

#### 2.5.1. GTT in 14-h Fasted Males

**Figure 4:**
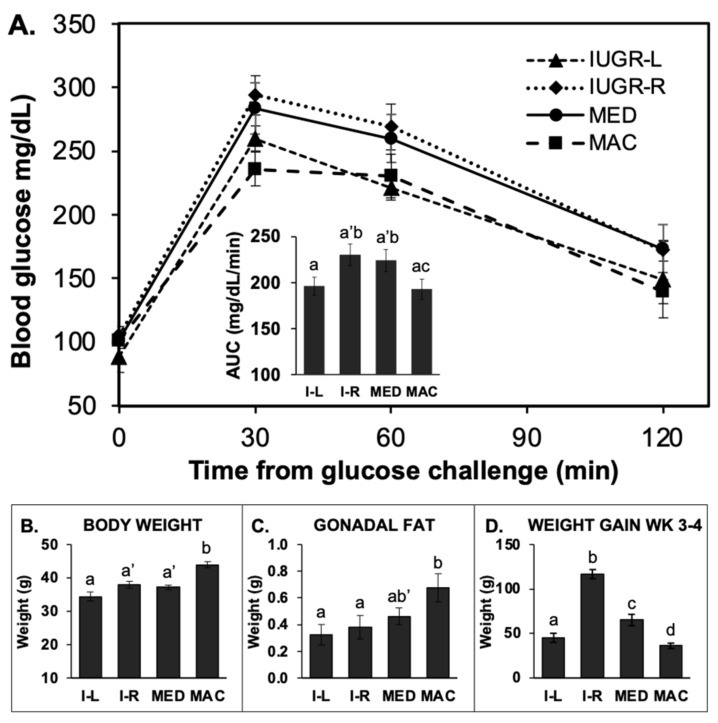
Glucose tolerance test in 14-h fasted Block 1 males. (**A**) glucose tolerance test data for IUGR-L (I-L; *n* = 6), IUGR-R (I-R; *n* = 9; one animal died in adulthood), median (MED, *n* = 16) and macrosomic (MAC, *n* = 16) males that underwent a 14 hr-fast prior to glucose challenge. Inset: Area under the curve (AUC) for this test (a’ *p* < 0.08 vs. IUGR-L). (**B**) body weight at the time of tissue collection following the GTT (a’ *p* < 0.08 compared to IUGR-L). (**C**) gonadal fat weight (b’ *p* = 0.06 compared to macrosomic). (**D**) the week 3–4 percent weight gain for these males. Groups with different letters above the error bar are significantly different from each other; groups that share a letter in common are not statistically different; letters with a dash indicate *p* values between 0.05 and 0.01 as indicated above. Values are mean ± SEM.

We observed a significant difference in response to the glucose challenge between median and IUGR-R compared to macrosomic and IUGR-L males. Specifically, blood glucose concentrations after the glucose challenge were similar in animals for the median and IUGR-R birth weight males, which showed impaired glucose tolerance in comparison to macrosomic and IUGR-L males based on the area under the blood glucose concentration-time curve (AUC; Figure 4A). Importantly, males in the slower post-weaning growth sub-group of IUGR males (IUGR-L) had better glucose tolerance than the heavier IUGR sub-group (IUGR-R) males (Figure 4A). Specifically, blood glucose concentrations tended to be lower in IUGR-L males compared to the IUGR-R males based on AUC (*p* = 0.06), and at both 30 and 60 min after glucose challenge (*p* = 0.08 for each comparison). What was surprising was that the median males had impaired glucose tolerance relative to the macrosomic males (*p* < 0.05). Macrosomic males’ body weight and gonadal fat pad weight were greater than all other groups (Figure 4B,C).

#### 2.5.2. GTT in 4-h Fasted (Block 2) Males

**Figure 5:**
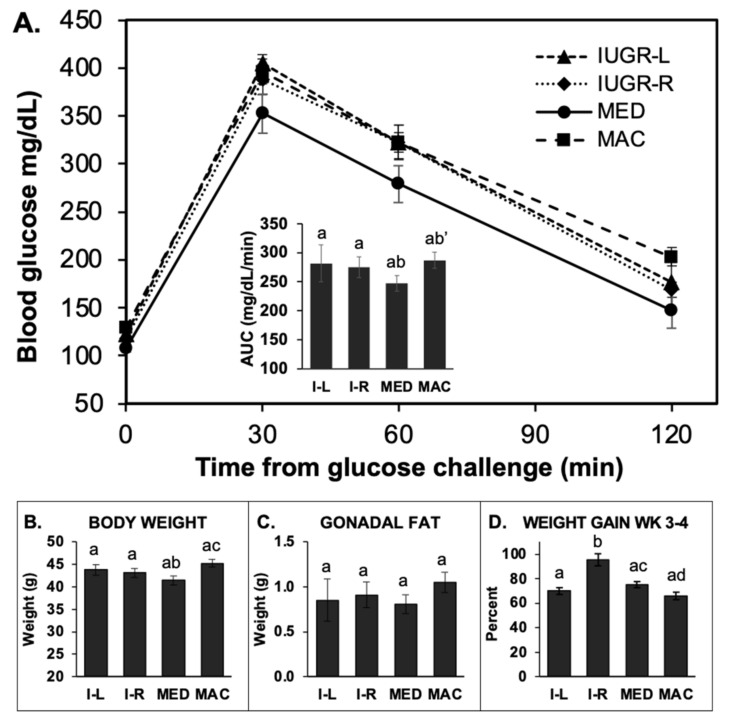
Glucose tolerance test in 4-h fasted Block 2 males. (**A**) glucose tolerance test for male IUGR-L (I-L; *n* = 7), IUGR-R (I-R; *n* = 19), median (MED; *n* = 25) and macrosomic (MAC; *n* = 25) mice that underwent a 4-h fast prior to glucose challenge. Inset: Area under the curve (AUC) for this test. The macrosomic males had reduced glucose tolerance relative to median males (b’ *p* = 0.055). (**B**) body weight at the time of tissue collection following the GTT. Macrosomic males were significantly heavier than IUGR-R and median males (*p* < 0.05). (**C**) gonadal fat weight. While gonadal fat pad weight was slightly greater in macrosomic males, it did not differ significantly between the groups. (**D**) the week 3–4 percent weight gain for these males. Groups with different letters above the error bar are significantly different from each other; groups that share a letter in common are not statistically different; letters with a dash indicate *p* values between 0.05 and 0.01 as indicated above. Values are mean ± SEM.

The results from the 4-h fasted males in Block 2 were different from those observed for the 14-h fasted males. First, the blood glucose levels were markedly higher for the 4-h fasted males relative to 14-h fasted males both at baseline and at 30 and 60 min after glucose injection. Second, based on AUC for 4-h fasted males, macrosomic males had reduced glucose tolerance relative to the median males (*p* = 0.055; Figure 5A), while both IUGR-L and IUGR-R showed somewhat lower glucose tolerance but did not differ significantly from median males.

### 2.6. Body Weight, Organ Weights, Fat Pad Weights, and Gonadal Fat Pad Adipocyte Number and Size: Comparison of 14-h Fasted and Non-Fasted Block 1 Males

#### 2.6.1. Data from 14-h Fasted Males

**Figure 6:**
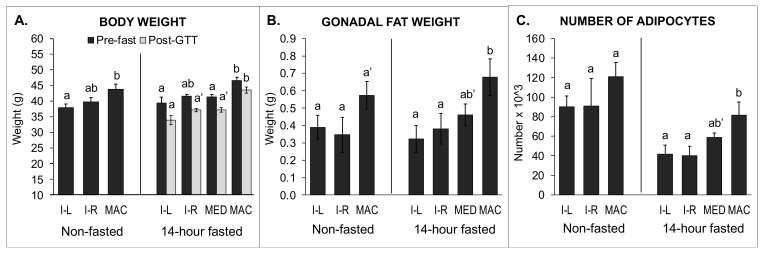
Comparison of body weight, fat pad weights, and adipocyte number in non-fasted vs. 14-h fasted animals. Data are from non-fasted males (IUGR-L *n* = 4, IUGR-R *n* = 5 and macrosomic *n* = 5) that did not undergo a GTT, and males fasted for 14 h prior to GTT and fat pad collection (IUGR-L *n* = 5, IUGR-R *n* = 8, median *n* = 14, macrosomic *n* = 13). (**A**) body weights, (**B**) gonadal fat pad weights, and (**C**) the number of adipocytes per total mass of fat. Groups with different letters are significantly different from each other; groups that share a letter in common are not statistically different; a’ and b’ *p* < 0.1. Values are mean ± SEM.

**Figure 7:**
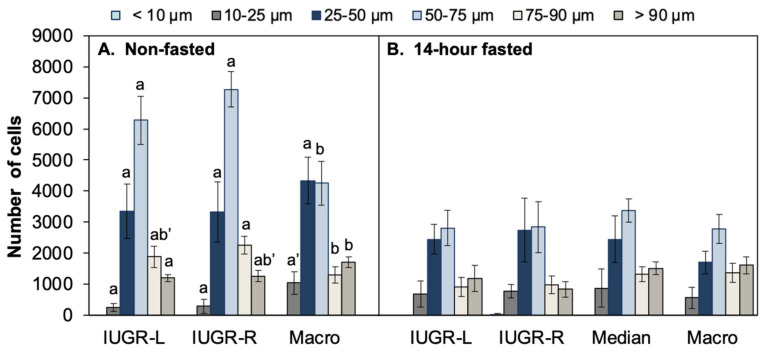
Gonadal adipocyte size distribution. Distribution of size categories for gonadal adipocytes collected from (**A**) non-fasted and (**B**) 14-h fasted males. For non-fasted males (**A**), the IUGR-L and IUGR-R males had significantly more mid-size adipocytes relative to macrosomic males, while macrosomic males had more of the largest adipocytes. a’ *p* < 0.1; b’ *p* < 0.08. For the 14-h fasted males, there were no significant differences between males from the different birth weight categories in any of the size categories (**B**). Different letters indicate statistically significant differences between birth weight groups within a size category (*p* < 0.05); groups that share a letter in common are not statistically different; letters with a dash indicate *p* values between 0.05 and 0.01 as indicated above. Values are mean ± SEM.

An important issue identified in this experiment was that the 14-h fasting procedure prior to the glucose tolerance test reduced body weight in all groups of males (IUGR-L: 12.5%, IUGR-R 9.2%, median: 11%, macrosomic: 6.3%), although less so in macrosomic males compared with IUGR-L males (Figure 6A). Weight loss in mice due to fasting is consistent with prior findings [25]. This weight loss resulted in the non-fasted IUGR-L and IUGR-R males being heavier at the time of fat pad collection than fasted IUGR animals. The 14-h fast altered the adipocyte size distribution (Figure 7) and also affected gene expression in gonadal fat collected after the GTT experiment relative to non-fasted males from the same prenatal growth category groups (see Section 2.7).

In 14-h fasted males, there were significant effects of birth weight category on body weight both prior to the GTT and also at the time of fat pad collection. Both prior to testing and after the 14-h fast and GTT, macrosomic animals were significantly heavier than IUGR-L and median animals (Figure 6A). IUGR-R animals were not statistically different from IUGR-L and median animals prior to testing, but at the time of collection, following the GTT, IUGR-L animals tended to be lighter than IUGR-R and median animals (*p* < 0.1, Figure 6A).

There were significant differences between IUGR-R and macrosomic males in heart, kidney and spleen weights, with macrosomic males having significantly heavier organ weights (Table S2). There were also significant differences between the different groups of 14-h fasted males in the weights of the gonadal fat pad weight collected immediately after the GTT test that indicated an effect of birth weight category. The gonadal fat pads from the macrosomic males were significantly heavier than for IUGR-L and IUGR-R males and tended (*p* = 0.07) to be heavier than the median male fat pads (Figure 6B; Table S3). The total amount of fat (summed renal, inguinal and gonadal fat pad weights) was significantly greater in macrosomic males than either IUGR or median animals (Table S3).

The total number of adipocytes in the gonadal fat pads is shown in Figure 6C. Macrosomic males had the highest mean adipocyte count and a significantly greater number of gonadal adipocytes than either IUGR-L males or IUGR-R males. Males in the median group tended to have fewer gonadal adipocytes than macrosomic males (*p* < 0.1). For these 14-h fasted males, there were no differences in the size distribution of gonadal adipocytes from IUGR-L, IUGR-R, median or macrosomic animals (Figure 7B).

#### 2.6.2. Data from Non-Fasted Males

In non-fasted males, there were significant effects of birth weight category on body weight at the time of fat pad collection, with macrosomic animals being heavier than either IUGR group (Figure 6A).

Gonadal fat pad weights did not differ significantly between IUGR-L, IUGR-R and macrosomic animals (Figure 6B) although gonadal fat pads from macrosomic males tended (*p* = 0.07) to be heavier than those of IUGR-R males. Total fat weight was significantly higher in macrosomic animals compared to either IUGR group (Table S3).

Total adipocyte number did not differ significantly for non-fasted males (Figure 6C). However, analysis of adipocyte size distribution in gonadal fat pads of non-fasted males indicated that the size distribution was similar for IUGR-L and IUGR-R males and that both were significantly different from the macrosomic males, that had fewer adipocytes in the mid-size range (*p* < 0.05; Figure 7A). In contrast, macrosomic males had significantly more large adipocytes compared to IUGR-L and IUGR-R males. Thus, in non-fasted males, macrosomic males had a significantly different gonadal adipocyte size distribution relative to either IUGR-L or IUGR-R males.

### 2.7. Gonadal Adipose Tissue Gene Expression by qPCR

**Figure 8:**
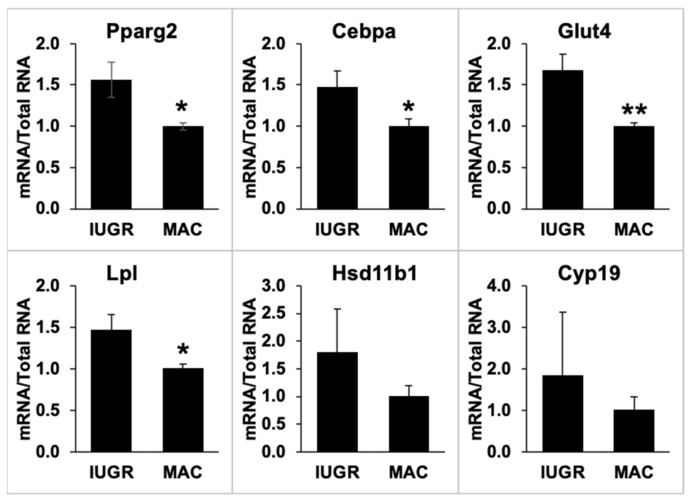
qPCR quantification of gene expression in gonadal fat of non-fasted IUGR and macrosomic male mice (*n* = 4–5 per group.) Values for IUGR animals are expressed relative to values for the macrosomic animals. Values are mean ± SEM. * *p* < 0.05, ** *p* < 0.01 compared to IUGR.

Six target genes were selected for analysis by qPCR based on their known roles in adipose tissue function. Gene expression was first measured in samples prepared from the gonadal fat from non-fasted males not previously administered a GTT, and these results are shown in Figure 8. Due to the limited number of IUGR and macrosomic males examined, we did not attempt to separate the IUGR males into IUGR-L and IUGR-R categories.

In non-fasted animals, the expression of Pparg2, Cebpa, Glut4 and Lpl was significantly higher in IUGR animals than in macrosomic animals (*p* < 0.05 for Pparg2, Cebpa and Lpl; *p* < 0.01 for Glut4). There were no significant differences between IUGR and macrosomic animals in the expression of Hsd11b1 and Cyp19, which appeared to be due to the high variance in samples from IUGR males.

We also measured the expression of these genes in fat samples from selected 14-h fasted male mice. In fat collected after the long fasting conditions and GTT, no differences were seen between the IUGR and macrosomic animals in the expression of these same six genes (Figure S3). Details of postnatal growth and body fat of the non-fasted and 14-h fasted animals whose tissues were used in the qPCR analyses are presented in Table S4.

### 2.8. Gonadal Adipose Tissue Gene Expression by Microarray Analysis

We conducted a microarray analysis of gene expression in gonadal fat from non-fasted males that had not experienced a GTT. We examined 4 macrosomic males, 3 IUGR-L males and 3 IUGR-R males. Postnatal growth, body fat and other characteristics of these animals are presented in Table S5. A list of differentially expressed genes is given in Table S7.

Analysis of the microarray data was specifically aimed at identifying differences between the IUGR-L and IUGR-R groups, and between the IUGR-R and macrosomic animals (median body weight at birth males were not examined). Initial analysis of the array data was performed using a two-tailed *t*-test with Benjamini-Hochberg correction and a two-fold difference cutoff, and further analysis was conducted by *t*-test for the comparisons of interest. Impacted signaling pathways and gene ontologies were also identified. In addition, we used ANOVA to identify genes that were differentially expressed among the pooled microarray data from all three treatment groups.

#### 2.8.1. Direct Comparison of Gene Expression in IUGR-L, IUGR-R and Macrosomic Males

**Figure 9:**
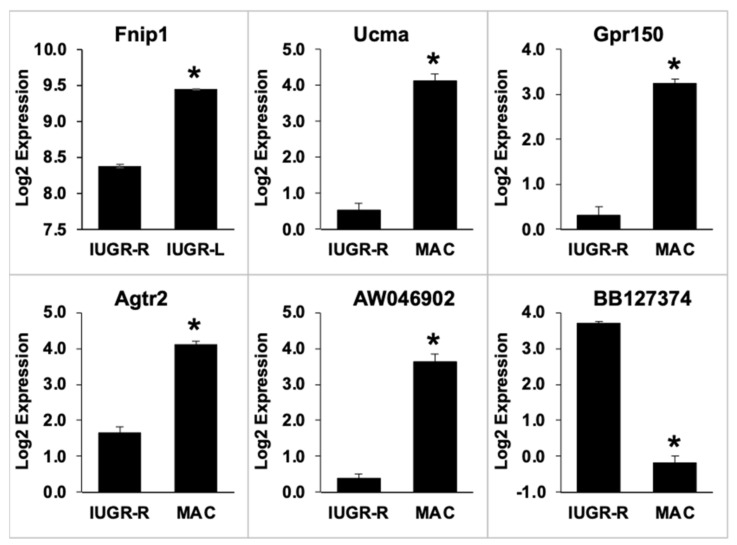
Differentially expressed genes. Statistical comparisons were between either IUGR-L and IUGR-R animals or between IUGR-R and Macrosomic animals. * *p* < 0.05 compared to IUGR-R. *p* values are Benjamini-Hochberg-corrected; uncorrected *p* values are all < 0.001. Values are mean ± SEM.

Examining the two IUGR groups, from the *t*-test with Benjamini-Hochberg correction, only one gene was identified as being differentially expressed between the two IUGR groups: Fnip1 (folliculin-interacting protein 1) was down-regulated 2.1-fold in the IUGR-R animals compared to the IUGR-L animals (Figure 9). When the data were analyzed without the multiple hypothesis correction, 1197 genes were identified as being differentially expressed between the two groups. KEGG pathways impacted (Table 1) included Type II Diabetes Mellitus (down-regulated in IUGR-R samples, z-score = 3.86) and Fat Digestion and Absorption (down-regulated in IUGR-R samples, z-score = 2.12).

For the IUGR-R vs. macrosomic comparison, analysis with Benjamini-Hochberg correction identified 5 differentially expressed genes, which included Ucma (unique cartilage matrix-associated protein, down-regulated 12.2-fold in macrosomic animals), Gpr150 (G protein-coupled receptor 150, down-regulated 7.6-fold) and Agtr2 (angiotensin II receptor, type 2, down-regulated 5.6-fold). The relative expression of these and other genes is shown in Figure 9. Analysis without the multiple hypothesis correction yielded a list of 659 genes. Impacted KEGG pathways identified (Table 1) included Carbohydrate Digestion and Absorption (up-regulated in IUGR-R, z-score = 4.67) and Fat Digestion and Absorption (up-regulated in IUGR-R, z-score = 4.09).

#### 2.8.2. Clustering Analysis of Differentially Expressed Genes

**Figure 10:**
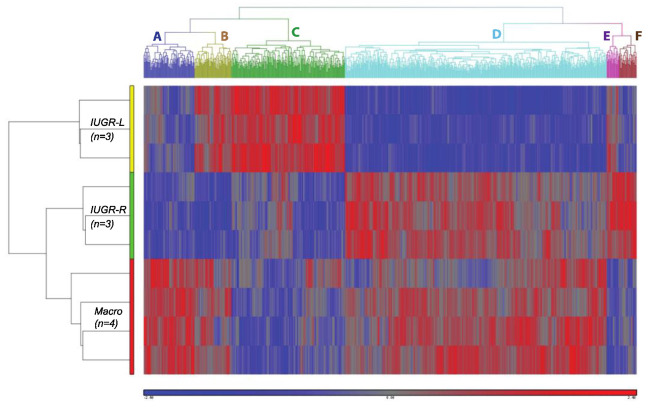
Unsupervised hierarchical clustering analysis of transcriptomes of gonadal fat. The heatmap shows differential expression of gene clusters in male adipose tissue from IUGR-L (first three rows; yellow bar), IUGR-R (center three rows; green bar) and macrosomic (“Macro”, last four rows; red bar) groups. Each column represents a gene and each row represents a different animal. Red indicates up-regulation and blue indicates down-regulation; grey indicates unchanged expression. Six clusters were identified (A–F at top). Genes were identified by ANOVA and were significant at *p* < 0.01.

Due to the small sample size, ANOVA was conducted without multiple hypothesis correction, yielding 855 genes that were differentially expressed among all groups at *p* < 0.01. These genes were subjected to hierarchical clustering, yielding the heatmap in Figure 10. The heatmap shows the separation of six clusters of differentially expressed genes identified as A–F, which segregate by birth weight, post-weaning growth rate, or adult weight.

An unsupervised hierarchical clustering analysis revealed that the macrosomic group was linked closely to the IUGR-R group (red and green bars on the left) and separated from the IUGR-L group (yellow bar). This was largely due to the gene Clusters C and D, which together (651 genes) represent 76% of the entire set of differentially expressed genes. Cluster D represents the largest gene node consisting of 454 genes (53%) that were more strongly expressed in the macrosomic and IUGR-R groups compared to the IUGR-L group, and Cluster C represents the second-largest node consisting of 197 genes (23%) that were more strongly expressed in the IUGR-L group compared to the Macrosomic or IUGR-R groups. These data show a close similarity of abdominal (gonadal) adipose tissue gene expression in IUGR-R and macrosomic males compared to IUGR-L males.

The clusters were subjected to gene ontology (GO) analysis using DAVID, but enrichment results were limited. No significant GO results were obtained for Clusters A, B, E or F. Cluster C genes were enriched in phosphoprotein, acetylation, nucleus, mRNA splicing, mRNA processing, Spliceosome, cell division, and ubl conjugation. Cluster D genes were enriched in phosphorylation, acetylation, cytoskeleton, phosphoprotein, and cell division. The biological significance of these enrichments was not clear. Combining Clusters C and D drove enrichment toward cell division, especially spindle and microtubule formation, but overall these GO results did not provide ready explanations of the observed phenotypic differences between the three groups of animals. Similar results were obtained using DAVID to identify impacted pathways. No significant functional pathways were obtained for Clusters A, B, E or F when analyzed singly. Submitting Cluster C indicated effects within Spliceosome, transcriptional misregulation in cancer, and oocyte meiosis. More pathways were indicated for Cluster D, and included B cell receptor signaling, MAPK signaling and Ras signaling, and pathways in cancer. Combining Clusters B and F pointed toward MAPK signaling and combining Clusters C and D pointed toward B cell receptor signaling, MAPK signaling and Ras signaling, among others.

It is likely that the small sample size limited the sensitivity of the formal analyses. In both the results from the *t*-test comparisons and those of the clustering analysis, we identified changes in the expression of several genes of interest that were not assigned to pathways by the software, several of which were directly relevant to adipocyte function. Accordingly, as an additional/alternative approach, we drew from both sets of data and identified groupings of genes that suggested potential impacts within specific signaling pathways. Examples (Table S6) were effects on the renin-angiotensin system (eight genes), PPAR signaling (five genes), adipocytokine signaling (5 genes) and glycolysis and gluconeogenesis (five genes), all of which generally distinguished between heavier compared to lighter-weight adult animals.

We also found changes in the expression of a number of genes in the Krüppel-Like Factor (Klf) family. *t*-test results identified a 2.23-fold up-regulation of Klf9 in IUGR-R vs. IUGR-L animals; expression was similar in macrosomic and IUGR-R animals. Klf2, Klf4 and Klf13 were present in Cluster D and Klf6 was present in Cluster A. Thus, for four of these five genes (Klf2, Klf4, Klf9 and Klf13) expression was higher in the heavier-in-adulthood macrosomic and IUGR-R males, while Klf6 expression was higher as a function of birth weight category (Figure S4A).

In addition, we saw standalone effects on three genes of interest, all of which were downregulated in IUGR-R compared to IUGR-L: Tbx15 (T-box transcription factor 15, an early patterning gene recently identified as a master regulator of obesity genes [26]) and Repin1 (replication initiator 1, strongly associated with adipogenesis) were down-regulated 13.16-fold and 2.36-fold respectively in IUGR-R, and in both cases expression was similar in IUGR-R and macrosomic samples. Dlk1 (delta-like 1 homolog) was down-regulated 2.12-fold in IUGR-R compared to IUGR-L (Figure S4B).

## 3. Discussion

Using a novel crowded uterus model, we evaluated the consequences of IUGR and macrosomia on outcomes associated with metabolic syndrome. An important part of this work was the comparison of IUGR males that experienced a rapid post-weaning increase in body weight (IUGR-R males) and those that did not (IUGR-L males). At this time, we do not have an explanation for why IUGR males segregated after weaning into rapid and low growth groups. IUGR-R males showed about a 2.5-fold greater weight gain than IUGR-L males during the first week after weaning, and by 12 weeks of age, IUGR-R males had reached the same body weight as macrosomic animals and were significantly heavier than slow-growing IUGR males. At six months of age, IUGR-R males showed impaired response to a glucose challenge compared to IUGR-L males and altered expression of many genes in abdominal adipose tissue was revealed by microarray analysis. Clustering analysis of gonadal fat transcriptomes (discussed further below) showed that rapidly-growing IUGR males (IUGR-R) were aligned with macrosomic males in outcomes that are related to metabolic abnormalities throughout life [6]. However, at that age IUGR-R males differed from macrosomic males in terms of body weight, fat weight, adipocyte counts and size, as well as heart, kidney and spleen weights. There were also differences in abdominal fat gene expression between IUGR-R and macrosomic males revealed by microarray analysis.

Thus, these findings provide evidence for two distinct pathways to abdominal fat gene regulation and glucose regulation, and reveal that IUGR that is followed by a rapid period of weight gain during postnatal life results in changes in adipose tissue gene activity that in many respects are similar to those seen due to macrosomia at birth. However, there are also significant differences between the IUGR-R phenotype and the macrosomic phenotype, and both IURG-L and IUGR-R males were in some respects more similar to each other than to macrosomic males. These results are complex, and it is not possible at this time to predict disease or metabolic outcomes in these animals. Clearly, more research is needed to unravel the complex interaction of fetal under- or over-growth with postnatal growth trajectories in order to understand the factors that lead to adult overweight, which, itself, is a predictor of dysregulation of abdominal adipocyte gene expression.

A consequence of IUGR (often associated with a period of rapid postnatal weight increase), as well as macrosomia, is the development of glucose intolerance and other co-morbidities of obesity [2,15]. Using the 14-h fasting procedure, the faster-growing IUGR-R males showed reduced glucose tolerance compared to the slower-growing IUGR-L and macrosomic males. We did not anticipate that macrosomic animals would have superior glucose tolerance; however, since the median birth weight animal also showed reduced glucose tolerance compared to these groups; this test did seem to distinguish between animals with slow and more rapid post-weaning weight gain. Using the shorter 4-h fast, both of the IUGR groups and the macrosomic males showed reduced glucose tolerance relative to males in the median birth weight category, although the comparisons did not reach statistical significance; it is likely that the fasting time was too short to allow for sensitive testing. Thus, under the 14-h fasting regime, there was a tendency for faster-growing animals to have reduced glucose tolerance, and under the 4-h regime, both the IUGR and macrosomic animals tended toward impaired glucose tolerance. It was beyond the scope of this study to include insulin measurements in these animals, but this would provide very valuable information on glucose regulation in these animals and should be included in future work.

The 14-h fasting procedure is a common approach for a mouse GTT, but we show here that the consequences to the animal may preclude further useful analyses. Although a decrease in body weight with fasting is consistent with other findings [25], the degree of weight loss observed in these animals was suggestive of physiological stress and indicated that alternative testing protocols should be considered in future work, as discussed elsewhere [27]. In this study, macrosomic animals appeared to be more resilient in the face of this fast; the degree of body weight loss was less, fat mass and adipocyte counts were less impacted, and glucose tolerance was less impacted compared to IUGR-L males that lost a greater percentage of their body weight during an overnight fast than IUGR-R or macrosomic males. Taken together with other findings, these results suggest that IUGR creates a phenotype that is sensitive to environmental stressors, such as starvation, relative to non-IUGR animals. We also showed elsewhere that another source of environmental stress, namely, exposure during fetal life to a very low dose of the manmade toxic chemical bisphenol A (BPA), exacerbated the rate of postweaning growth in low birth and low weaning weight male mice, and also significantly impaired glucose tolerance [28]. BPA is one of many manmade chemicals referred to as metabolic disrupting chemicals [1,29].

We did not monitor weights between week 12 (three months) and six months of age, but by six months body the weight differences seen at 12 weeks had shifted, and the two IUGR groups were more similar and significantly lighter than macrosomic animals. This may reflect slowed growth during this period in IUGR-R animals, but the reason for this shift is not clear and further investigation is needed. Of note, in the Goto-Kakizaki diabetic rat, growth slows, and adipose tissue accumulation stops after week 12 [30], and it is interesting to speculate on possible mechanistic similarities although these are very different models. Overall, for body weight, there is a birth weight effect but also a post-weaning growth rate effect that is age-dependent.

Birth weight category appeared to strongly influence both fat weight and adipocyte number and size. In both 14-h fasted and non-fasted animals, macrosomic animals had higher amounts of total fat; gonadal fat pads tended to be heavier in macrosomic animals compared to either IUGR group, which did not differ, although this did not reach significance in non-fasted animals. Analysis of adipocyte size distribution in non-fasted animals clearly showed a difference between the two IUGR groups and macrosomic animals. After the 14-h fast this difference was eliminated, owing to a marked decrease in mid-sized adipocytes in IUGR-L and IUGR-R males, and a smaller decrease in these adipocytes in macrosomic males. However, the Coulter counter would not have detected any particle than ~8 µm diameter, so the apparent loss of adipocytes due to the 14-h fast might also reflect such a significant decrease in adipocyte size that they were not counted.

Analysis of gene expression revealed further differences and similarities between IUGR and macrosomic animals. The up-regulation of Pparg2, Cebpa, Glut4 and Lpl expression in IUGR males (or down-regulation in macrosomic males) is consistent with findings of clustering effects on PPAR signaling identified in the microarray analysis. This indicates the effects of IUGR on genes associated with an increased predisposition to store fat in adipocytes and the potential development of obesity-related abnormalities. This finding may be reflected in the increased number of mid-sized adipocytes observed in IUGR compared to macrosomic animals, although gonadal fat weights do not differ between these two groups of non-fasted animals. To confirm these results, future experiments should consider confirmation of effects on gene expression through Western blot and other approaches.

The objective of the microarray analysis was to provide preliminary information regarding the potential pathways in gonadal adipocytes that differed based on the rate of fetal growth as well as the rate of postnatal growth. Importantly, clustering analysis demonstrated that in terms of gene expression the association between the heavier-in-adulthood (week 12) IUGR-R and macrosomic animals was closer than the association between low birthweight IUGR-L and IUGR-R animals; the similarity of IUGR-R and macrosomic adipocyte gene expression was in spite of the very different birth weights, and also in spite of the more similar adipose tissue weights and adipocyte counts in the two groups of IUGR animals. In the mouse, adipocyte number may be determined prenatally but adipogenesis is initiated after birth [31]. Thus, although the amount of fat and adipocyte size in the adult may be influenced by the relatively low prenatal and pre-weaning nutrient availability, the accelerated growth during the immediate postweaning period occurred during the ongoing development of adipose tissue and permanently impacted overall gene expression in this tissue. Our results are limited by the small sample size, but we identified differences in metabolic pathways related to fat digestion and absorption, carbohydrate digestion, and type II diabetes mellitus signaling. We showed using clustering analysis that the expression of many genes in adipose tissue was influenced by either fetal growth or post-weaning growth or both.

Two of the differentially expressed genes identified using the more stringent *t*-test were particularly interesting. Fnip1, down-regulated in IUGR-R adipose tissue compared to IUGR-L tissue, codes for a protein that interacts with folliculin, which in turn interacts with the AMPK and mTOR signaling pathways [32,33]. The known involvement of AMPK and mTOR signaling in cellular energy and nutrient sensing [34] may be relevant here although our results did not point specifically toward alterations to either pathway.

The reduced expression of Agtr2 in IUGR-R animals relative to macrosomic animals is also interesting since it is thought that an overactive renin-angiotensin system (RAS) is involved in metabolic syndrome [35]. Increased angiotensinogen (AGT) production by white adipose tissue has been related to both obesity and hypertension, and loss of Agtr2 expression is sufficient to rescue obesity induced by adipose tissue AGT overexpression [36]. We found additional effects on the RAS pathway by manual searching rather than through selection by the software, and these results generally distinguished between heavier (at week 12) and lighter animals. Although the overall direction of the impact on this pathway is not clear, the multiple hits do suggest a possible association between our fetal/postnatal growth paradigm and altered signaling within the renin-angiotensin system.

Recent work in cardiomyocytes has shown cross-talk between mTOR and RAS signaling, acting in part through changes to Pik3 expression [37,38]. It is possible that our results reflect this: although the link to the mTOR pathway is only by association with the effects of Fnip1, we did see up-regulation of Pik3 in IUGR-R compared to IUGR-L and a general indication of effects on the RAS pathway in Clusters C and D (heavier vs. lighter animals). Although these are very different cell types, these findings may provide some mechanistic information.

The Klf family of genes are widely involved in diverse biological processes that include adipogenesis and adipocyte differentiation, many of which interact with Cebpa or Cebpb and/or Pparg [39,40,41]. Of the five members of this family identified in our dataset, Klf4, Klf6 and Klf9 promote adipogenesis, Klf2 suppresses adipogenesis, and Klf9 and Klf13 are pro-adipogenic transcription factors [40,42]. The general trend in our data was for the expression of these genes to be higher in the heavier (IUGR-R and macrosomic) animals. Klf6 promotes adipocyte differentiation via suppression of the pre-adipocyte differentiation factor Dlk1 [43], and since our data show reduced expression of Dlk1 in IUGR-R compared to IUGR-L, this is generally consistent and point to a role of this gene family in the observed growth effects.

Alterations to adipocyte morphology can be directly related to alterations in adipocyte function as well as to insulin resistance [44,45]. Repin1 expression is reported to be increased during adipogenesis and reduced expression is associated with reduced adipocyte size as well as altered glucose uptake in 3T3 cells [46]. The observed down-regulation of Repin1 in macrosomic animals compared to IUGR-L may be consistent with the reduced number of smaller and mid-size adipocytes that we observed in macrosomic animals. However, Repin1 expression was also down-regulated in IUGR-R animals, which had a very similar adipocyte size distribution to that of IUGR-L animals, so it is not clear if whether Repin1 expression directly relates to adipocyte size in these animals or whether another factor modulates the effect in IUGR-R animals.

**Figure 11:**
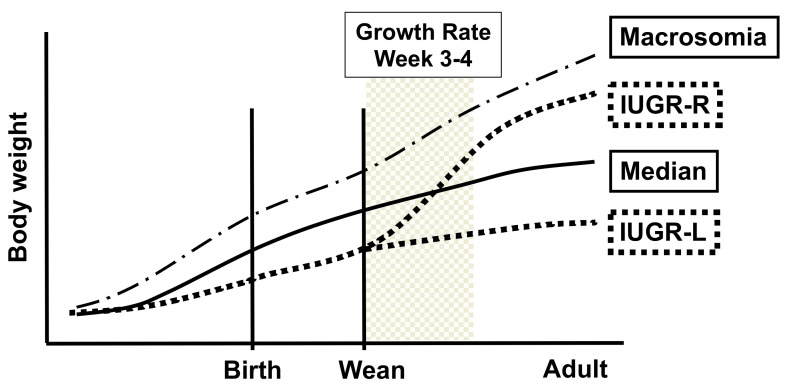
Centile crossing by IUGR-R male mice. IUGR-R mice show 120% increase in body weight during the first week (week 3–4) after weaning that by week four results in IUGR-R males having a body weight similar to that of males that were macrosomic at birth (see Figure 3).

The phenomenon of “centile crossing” is well documented for IUGR babies that experience a rapid increase in body weight and fat in early childhood (here, IUGR-R males), while other IUGR babies (here, IUGR-L males) do not show this (Figure 11). There are significant adverse consequences that occur as a result of rapid weight gain in childhood that creates an obese phenotype [6], referred to as a “thrifty phenotype”, and leads to the spectrum of metabolic diseases related to obesity, including glucose intolerance, that are extremely difficult to reverse [1,15,47]. As a result of a dramatic increase in body weight immediately after weaning, IUGR-R males exhibit postweaning growth rate centile crossing, and thus the prenatal and postnatal nutrient mismatch that is theorized in the thrifty phenotype hypothesis. This could occur even if a child is subsequently provided with a nutritionally balanced diet, although the impact on obesity would be greatly exacerbated with a highly processed, high-calorie meal typical of the standard Western diet [48].

Other developmental studies of IUGR have focused on exposures to environmental stressors, such as metabolic disrupting chemicals, physiological/psychological stress, and the consequences of consumption of processed foods instead of the nutrient (breast milk) that infants evolved to consume for far longer than is common in developed countries [1,49,50]. Not surprising is that our preliminary findings of different outcomes in males and females are, in fact, a common outcome [51].

While our prior findings suggest that the IUGR male mice were first malnourished due to decreased transplacental nutrient availability in utero, they were also possibly malnourished prior to weaning, potentially due to competition for resources against larger siblings (there were ~2 more pups per litter than nipples), although this remains to be investigated. It is also necessary to examine the neural control systems that regulate food hunger and satiety in IUGR-L and IUGR-R as well as macrosomic males to determine how they differ from median body weight at birth males. It was only in the period immediately after weaning when the IUGR mice had the ability to feed freely that differences between IUGR-L and IUGR-R males became apparent. It is during this brief post-weaning period that should be the focus of future studies, even though we were still able to identify differences in GTT and gene activity in the adipose tissue when the mice were examined much later in adulthood.

## 4. Materials and Methods

### 4.1. Animal Husbandry

CD-1 mice (*Mus domesticus*) were purchased from Charles River Breeding Laboratories (Wilmington, MA, USA) and maintained as an outbred colony with periodic replacement. The mice used in this study were housed in 18 × 29 × 13 cm polypropylene cages on corncob bedding. Pregnant and lactating mice were fed Purina mouse breeder chow 5008 (soy-based, Purina-Mills, St. Louis, MO, USA). After weaning, offspring were fed Purina standard laboratory chow 5001 (soy-based). Water was provided ad libitum in glass bottles and was purified by ion exchange followed by a series of carbon filters. Rooms were maintained at 25 ± 2 °C under a 12-h light:dark cycle, with the lights on at 1030 h. All animal procedures were approved by the University of Missouri Animal Care and Use Committee and conformed to the Guide for the Care and Use of Laboratory Animals of the National Institutes of Health. The animal facility is accredited by the Association for Assessment and Accreditation of Laboratory Animal Care, International (AAA-LAC).

### 4.2. Hemi-Ovariectomy Procedure

To examine the consequences of developing in a crowded uterine horn, offspring from two blocks of hemi-ovariectomized pregnant female CD-1 mice were examined. In Block 1, 52 postpartum dams were hemi-ovariectomized under ketamine-based anesthesia. In Block 2 an additional 21 postpartum dams were hemi-ovariectomized. We used postpartum dams so that we could examine offspring from the second litter, which has more pups than the first litter [23]; our objective was to increase the crowding of fetuses within one uterine horn. The left ovary was removed with a small incision, and the ovarian artery was cauterized. The left ovary was removed rather than the right ovary because of differences in the anatomy of the blood vessels associated with the left and right uterine horns.

Figure 1A depicts the normal female mouse reproductive tract with a duplex uterus. We have reported that the cranial end of the right utero-ovarian artery consistently inserts into the descending aorta, whereas the left utero-ovarian artery has a variable insertion into the descending aorta or the renal artery, including in some cases immediately adjacent to the left kidney. The variability in the left uterine horn vasculature at the cranial end of the uterus impacts placental blood flow at the cranial end of the uterine horn in both mice and rats [20,21]. Since placental blood flow impacts fetal growth [19,22], we chose to only examine offspring that developed in the right uterine horn to avoid variability due to the vascular anatomy of the left uterine horn. A schematic of the crowded uterine horn is shown in Figure 1B. This model has been shown to lead to differences in placental blood flow between fetuses located in the middle vs. the cranial or caudal ends of the uterus [22].

### 4.3. Mating of Females and Determination of Offspring Body Weights

Beginning five days after surgery, the hemi-ovariectomized female mice were singly housed with a stud male for up to 14 days. The day of natural birth was designated as postnatal day (PND) 1. At approximately 1400 h on the day of birth (PND 1), all pups were weighed and toe-clipped for identification, so that each individual could be categorized by birth weight percentile and then followed for the postnatal rate of growth and other outcomes. Toe-clipping was performed on the back paws of the animal using a set numbering system. Mouse pups were then not handled again between the day of birth and weaning at week 3 (postnatal day 21), since handling multiple times prior to weaning reduces pre-pubertal growth rate (unpublished observation). After the pups were weaned, the dams were euthanized with CO_2_ and then necropsied to confirm the location of implantation sites as well as the vascular anatomy of the right uterine horn. After weaning at week 3, all male and female offspring from all of the birth weight percentile groups were housed 3–4 siblings of the same sex per cage and were weighed once per week until they were 12 weeks old. Experimental animals were generated from two separate breedings, referred to below as Block 1 (52 litters) and Block 2 (21 litters). As stated in the Introduction, we defined animals below the bottom 5th percentile for birth weights as IUGR and animals above the 95th percentile as macrosomic. We defined animals in the median range of birth weights as median-at-birth.

### 4.4. Experimental Animals

Summarized information of the experimental animals, group designations and experiments are in Table S1 for reference.

#### 4.4.1. Block 1 Animals

All 605 animals (308 males and 297 females at birth) from 52 litters were used for the analysis of body weight between birth and postnatal week 12. After week 12, only males in the IUGR, median and macrosomic ranges were retained for further study.

The group of male mice selected from Block 1 for further study consisted of groups of IUGR, macrosomic and median-at-birth animals that were selected, respectively, from the bottom 9.9%, above the 93rd%, and close to the median (47.4–51.2%) of all birth weights (*n* = 29, 21 and 16, respectively). Based on the percent increase in body weight between postnatal week 3–4, we divided the IUGR males into low (IUGR-L, up to 65% weight gain) and rapid (IUGR-R, over 65% weight gain) subgroups, since there is evidence that IUGR interacts with the rate of postnatal growth in terms of risk for developing metabolic diseases [15]. Some of these male mice (IUGR-L *n* = 6, IUGR-R *n* = 10, median *n* = 16, and macrosomic *n* = 16) were used for glucose tolerance tests (GTT), fat pad (gonadal, renal and inguinal) and organ (liver, kidney, heart, spleen, testes, epididymides) collections, as well as analysis of gonadal adipocyte number and size; other males (IUGR-L *n* = 5, IUGR-R *n* = 5, and macrosomic *n* = 5) were used for further fat pad collections and adipocyte analysis, and also for analysis of gene expression by qPCR and microarray. While we followed the body weights of female siblings through postnatal week 12, due to an absence of body weight differences by week 12 in females (Figure S1), we focused here on males.

#### 4.4.2. Block 2 Animals

A second block of 21 hemi-ovariectomized pregnant females produced pups solely for a follow-up GTT test. These IUGR-L (*n* = 7), IUGR-R (*n* = 19), median (*n* = 24) and macrosomic (*n* = 25) males were selected using the above criteria (IUGR < 10th percentile of birth weight, and macrosomic > 93rd percentile for birth weight). The body weights of these animals were recorded to establish the post-weaning weight gain and again at 24 weeks old.

### 4.5. Glucose Tolerance Test (GTT)

For the first glucose tolerance test (GTT), we examined Block 1 IUGR-L, IUGR-R, median and macrosomic males (*n* = 5, 9, 16 and 16, respectively). The GTT was conducted prior to sacrifice when animals were approximately 6 months old. Animals were fasted for 14 h prior to the test, but water was available ad libitum. Following the fast, animals were weighed and injected i.p. with a single bolus of glucose at a dose of 2 g glucose per kg body weight in an average volume of 176 µL of saline. Serum glucose measurements were taken by tail nick just prior to glucose administration and again at 30, 60 and 120 min after glucose administration. Gonadal, renal and inguinal fat pads were collected and weighed from these males at sacrifice. The area under the concentration-time curve (AUC) for the 0–120 min prior to and after dosing was calculated using the linear trapezoidal rule.

We conducted a second GTT test on Block 2 males, using the same methods as described for the first GTT, except that in this study the animals only experienced a 4-h fast prior to the GTT. The 4 h commenced at the start of the light phase; mice do not eat much food during the early light phase of the light:dark cycle [52] and this was thus a minimal fasting period. Bodyweights were recorded prior to the GTT, and gonadal fat-pad weights were recorded at sacrifice. Animals were sacrificed at least 7 days after the GTT rather than immediately afterwards as for Block 1.

### 4.6. Collection of Gonadal Fat

Gonadal fat was collected from all 14-h fasted and non-fasted Block 1 males, for analysis of adipocyte number and size distribution and gene expression analysis. For all animals, the weight of both gonadal fat pads was recorded. A 50–60 mg portion of the fat was placed in 5.2 mL of 2.9% OsO_4_ in 0.05 M acidic collidine and retained for cell counting, and the remainder of the fat was snap-frozen in liquid nitrogen for later analysis of gene expression by qPCR and microarray analysis.

### 4.7. Analysis of Fat Cell Number and Volume

Fat from all Block 1 males was used in this study. The reserved 50–60 mg portion of the fat pad (above) was prepared for cell counting using a modification of the method of Hirsch and Gallian [53] as described by Kump and Booth [54], and cell number and size distribution were measured by the Coulter method [55]. Briefly, the fat was fixed and adipocytes were separated from other tissue in the OsO_4_-collidine solution for 3–4 weeks; this procedure results in free cells. The cells were washed with isotonic saline solution and left in saline for 24 h, and then washed with 8 M urea in saline and left in that solution for 3–4 days. The cells were finally rinsed with 0.1% Triton X-100 and filtered through a 250 µm filter onto a 10 µm filter. The collected cells were suspended in Isoton II (Beckman-Coulter, Fullerton, CA, USA) containing 10% glycerol. Cells were counted and cell size was determined on a Coulter Multi-Sizer II, with the particle counting window set to 8.03–271.1 µm. Cells were counted in three 15-s bursts, and the three counts were summed to give a single measure per 45 s period. Cell counts reported here are the total number of cells contained in the combined fat pads.

### 4.8. Analysis of Gonadal Fat Gene Expression

We used both qPCR and microarray analysis to examine gene expression in gonadal fat. For qPCR we used samples from both the 14-h fasted and non-fasted males; we selected 4–5 animals per IUGR and macrosomic group, but because of the low numbers we did not stratify the IUGR animals further into L and R categories. For microarray analysis we used samples from selected non-fasted animals only, using 4 of the five macrosomic males, 3 IUGR-L males, and 3 IUGR-R males.

RNA was prepared from 50–80 mg of gonadal fat pad samples. Fat was homogenized in TRI Reagent (Sigma, St. Louis, MO, USA), and RNA was isolated according to the manufacturer’s protocol, with a final precipitation using lithium chloride. The integrity of the RNA samples was verified by electrophoresis in 0.75% agarose gel. The RNA concentrations were measured spectrophotometrically. For microarray analysis, the samples went through a further cleanup on Qiagen RNeasy kit.

#### 4.8.1. qPCR Assay

Expression of specific mRNAs was measured by one-step qPCR as described by Bustin [56] with the TaqMan EZ RT-PCR kit (Applied Biosystems, Foster City, CA, USA) on the ABI PRISM 7700 Sequence Detection System (Applied Biosystems, Waltham, MA, USA). The concentrations of Mn2^+^, probe, and primers were optimized for each primer/probe set. Six target genes were selected based on their known roles in adipose tissue. Of these 6 genes, Pparg2 is a well-known master regulator of adipogenesis [57] that is also important for the regulation of glucose and lipid homeostasis and insulin sensitivity [58]. Cebpa interacts with Pparg2 to promote adipocyte differentiation as well as the transcription of glucose transporters, such as Glut4 (Slc2a4) [59]. These three genes are thus early-stage adipogenic genes [60]. Lpl is a central enzyme of lipoprotein metabolism that is important to the development of obesity, metabolizing lipoproteins and triglyceride particles, and producing fatty acids that are taken up by the adipocyte [61]. Hsd11b1 and Cyp19 are both candidate obesity genes [62].

Primer/probe sets for Hsd11b1 (11ß-hydroxysteroid dehydrogenase; GenBank accession no. NM-001044751.1) and Cyp19 (aromatase; GenBank accession no. NM-007810.3) were designed using Primer Express software (Applied Biosystems) and are shown in Table 2. Primers were designed to span exon boundaries in order to prevent the amplification of genomic DNA. Primers were synthesized by Invitrogen (Carlsbad, CA, USA), and probes were synthesized by Applied Biosystems. Other primer/probe sets, for Pparg2 (peroxisome proliferator receptor gamma 2), Ceba (CCAAT enhancer-binding protein α), Lpl (lipoprotein lipase) and Glut4 (glucose transporter type 4), were TaqMan Gene Expression Assays (pre-optimized and validated) obtained from Applied Biosystems; details are given in Table 3. The relative concentrations of specific mRNAs in each sample were normalized to total RNA per sample, as previously described [56,63,64].

#### 4.8.2. Microarray Analysis

Total RNA was isolated from gonadal fat as described above and purified with the RNeasy Mini kit (Qiagen, Valencia, CA, USA) according to the manufacturers’ instructions, and RNA quality was determined on an Agilent Bioanalyzer (Agilent, Palo Alto, CA, USA). The transcriptomal profiles were determined using Affymetrix mouse 430 2.0 microarrays, which assess over 39,000 gene transcripts. Scanned image data were converted into numerical tables using Affymetrix GeneChip Operating Software and Gene Expression Console. Data analysis and mining, including gene ontology enrichment analysis, were performed using GeneSifter (Giospiza Inc., Seattle, WA, USA) and Partek Genomics Suite (Partek Inc., St. Louis, MO, USA) Further functional characterization was performed using DAVID (The Database for Annotation, Visualization and Integrated Discovery; http://david.abcc.ncifcrf.gov (accessed on 12 January 2020) [65,66]. Microarray data were deposited in NCBI Gene Expression Omnibus (accession number GSE33761.)

### 4.9. Statistics

Data (other than microarray data, described above) were analyzed by analysis of variance or analysis of covariance (organ weights with body weight as the covariate) using SAS (v9.2, SAS Institute, Cary, NC, USA). In a few cases, data were log-transformed. Comparisons of overall statistically significant findings were made using Fisher’s LSD with Bonferroni correction for multiple comparisons. qPCR data were compared using *t*-tests. Statistical significance was set at *p* < 0.05).

## 5. Conclusions

We identified here the effects of both prenatal and postweaning growth on factors related to adult metabolic disease. While the effects of pre- and post-natal growth are complex, we have clearly identified two groups in this study that have characteristics of metabolic syndrome in adulthood: the first being the result of overgrowth during fetal life (macrosomic males), and the second resulting from an interaction of prenatal growth restriction followed by a very rapid rate of postnatal growth over a short period of time after weaning, which occurred in IUGR-R males. We describe an interesting set of similarities between macrosomic and IUGR-R males, which experienced very different pathways to elevated weight by the end of the first week of free-feeding after weaning, but also outcomes that revealed similarities between IUGR-L and IUGR-R males in comparison to macrosomic males. Our studies here provide an initial profile of these three groups of males, but future research will be required to move beyond these preliminary findings.

In summary, with the increasing incidence of obesity and its related co-morbidities throughout the developed world, further research is needed on any promising method to identify those at risk for obesity and to develop customized, realistic therapies for those individuals. Our crowded uterus model presents a novel method to study the progression of three different phenotypes, IUGR with rapid postweaning growth, IUGR without rapid postweaning growth and macrosomic animals. The objective of future experiments would be to better understand the different characteristics of these three groups (in comparison to median bodyweight at birth animals). A long-term objective would be the potential to develop focused treatments to not only mitigate the abnormal metabolic consequences for these different sub-groups, but to intervene in the processes that lead to abnormal adult phenotypes.

## Figures and Tables

**Table 1:** KEGG Pathways differentially impacted by growth. Statistical comparisons were between either IUGR-L and IUGR-R animals or between IUGR-R and Macrosomic animals.

Groups Compared	KEGG Pathway	Ratio	Direction	*p*-Value	Gene Identifier	Gene ID
IUGR-L	**Type 2 Diabetes Mellitus**	9.5	Up	0.002	NM_008840	Pik3cd
Vs. IUGR-R	down-regulated in IUGR-R	4.31	Down	0.047	BE943756	Cacna1a
	z score = 3.86	3.45	Up	0.019	NM_010438	Hk1
		2.92	Down	0.045	BB184171	Mapk8
		2.11	Down	0.004	BB205102	Pik3cg
		2.11	Down	0.002	BB048682	Cacna1d
		2.03	Down	0.038	BE947490	Mapk10
	**Fat digestion and absorption**	3.04	Up	0.025	BG070618	-
	down-regulated in IUGR-R	3.01	Down	0.047	NM_011128	Pnliprp2
	z-score = 2.12	2.56	Down	0.023	NM_025469	Clps
		2.05	Down	0.006	AI326372	Pnlip
Macrosomic vs.	**Carbohydrate digestion and** **absorption**	5.67	Up	0.019	NM_008840	Pik3cd
IUGR-R		2.89	Down	0.011	BI696040	Atp1a1
	up-regulated in IUGR-R	2.42	Up	0.028	NM_019741	Slc2a5
	z score = 4.67	2.22	Up	0.037	BC027319	Atp1b1
		2.2	Up	0.043	BC027319	Atp1b1
	**Fat digestion and absorption**	6.49	Up	0.048	AI194999	Apoa1
	up-regulated in IUGR-R	4.96	Up	0.015	NM_007980	Fabp2
	z-score = 4.09	4.29	Up	0.006	AI527359	Apoa1
		3.41	Up	0.019	NM_025469	Clps

**Table 2:** Sequences of primers and probes synthesized for real-time qPCR assays.

Gene Name		Sequence (5′–3′)
Hsd11b1	Forward	GCAGCATTGCCGTCATCTC
	Reverse	GAACCCATCCAGAGCAAACTTG
	Probe	TGGCTGGGAAAATGACCCAGCCTATG
Cyp19	Forward	CCGAGCCTTTGGAGAACAATT
	Reverse	TCCACACAAACTTCCACCATTC
	Probe	TTTCTTTATGAAAGCTCTGACGGGCCCT

**Table 3:** Primer/probe gene expression assays purchased for real time qPCR from Applied Biosystems.

Gene Name	Assay ID	Context Sequence
Pparg	Mm00440945_m1	TCAGTGGAGACCGCCCAGGCTTGCT
Cebpa	Mm00514283_s1	ACCAGCCACCGCCGCCACCGCCACC
Lpl	Mm00434764_m1	ATGGATGGACGGTAACGGGAATGTA
Glut4 (Slc2a4)	Mm00436615_m1	CTGCTGCTGCTGGAACGGGTTCCAG

## Data Availability

The microarray data have been deposited in the NCBI GEO database, accession number GSE33761. Other data presented in this study are contained within the article and Supplementary Materials and may be obtained from the authors.

## Supplementary Materials

The following supporting information can be downloaded at: https://www.mdpi.com/article/10.3390/metabo12020102/s1. Figure S1: Birth weight and growth of female mice from Block 1, Figure S2: Glucose tolerance test in 4-h fasted females, Figure S3: qPCR quantification of gene expression in gonadal fat in 14-h fasted IUGR and macrosomic male mice, Figure S4: Expression of selected genes of interest from the microarray data set, Table S1: Outline of Block 1 and Block 2 experiments conducted on male offspring. Table S2: Organ weights of 14-h fasted Block 1, Set 1 males. Table S3: Body weights and fat pad weights in 14-h fasted and non-fasted males, Table S4: Growth and fat weights of the sub-group of 14-h fasted males and of non-fasted males used in qPCR analyses, Table S5: Growth and fat weights of animals used in Microarray analyses, Table S6: Gene pathways manually identified as being potentially impacted by either prenatal or postweaning development, Table S7A, Microarray data: T-test results, Table S7B, Microarray Data: Genes identified by clustering analysis.
