# Supplementary material for: The Crowded Uterine Horn Mouse Model for Examining Postnatal Metabolic Consequences of Intrauterine Growth Restriction vs. Macrosomia in Siblings
Source: Metabolites. 2022 Jan 22;12(2):102. doi: 10.3390/metabo12020102 (PMC8880550; doi:10.3390/metabo12020102)
Supplement: Supplementary file 1 [file metabolites-12-00102-s001.zip › metabolites-1538699-SI.pdf]

SUPPLEMENTAL MATERIAL

The crowded uterine horn mouse model for examining postnatal metabolic consequences of intrauterine growth restriction vs. macrosomia in siblings

Julia A. Taylor[1], Benjamin L. Coe[1], Toshi Shioda[2], Frederick S. vom Saal[1]
[1]Division of Biological Sciences, University of Missouri-Columbia
[2] Center for Cancer Research, Massachusetts General Hospital and Harvard Medical School

Contents:

Figure S1: Birth weight and growth of female mice from Block 1,

Figure S2: Glucose tolerance test in 4-hour fasted females,

Figure S3: qPCR quantification of gonadal adipocyte gene expression in 14-hr fast-ed IUGR and macrosomic male mice,

Figure S4: Expression of selected genes of interest from the microarray data set,

Table S1: Outline of Block 1 and Block 2 experiments conducted on male off-spring.

Table S2: Organ weights of 14-hr fasted Block 1 males.

Table S3: Body weights and fat pad weights in 14-hr fasted and non-fasted males,

Table S4: Growth and fat weights of the sub-group of 14-hr fasted males and non-fasted males used in qPCR analyses,

Table S5: Growth and fat weights of animals used in microarray analyses,

Table S6: Gene pathways manually identified as being potentially impacted by either prenatal or postweaning development.

Table S7: Microarray data, differentially expressed genes. A) T-test results and B) Genes identified by clustering analysis.

Data for female offspring from Block 1 crowded uterus study

The mean weight for all female mice was 1.59±0.23 g (mean±SEM). Of the animals that survived to weaning, the weight range for females with birth weights in the bottom 5th percentile (designated as IUGR; n=15) was 0.72-1.21 g (mean±SEM: 1.04±0.04 g) (Figure S1). Females categorized as being at the median (~1.59 g) body weight range were in the 43.6-48.8 percentile of all birth weights. The weight range for females with birth weights in the top 5th percentile (designated as macrosomic) was 1.96-2.40 g (n=14; mean±SEM: 2.06±0.03 g). Overall, the mean weights in each percentile for body weight at birth were slightly lower for females than males. In females there were no differences in week 12 (adult) body weights based on body weight at birth category (Figure S1).

The relationship between bodyweight at birth and adult bodyweight in females was different from that observed in their male siblings. All females, regardless of birth weight, were statistically indistinguishable by weight at 4 weeks of age, and remained statistically similar until the end of the bodyweight measurements at week 12 (Figure S1-A). Specifically, at birth the females had a wide weight distribution, similar to that seen in males, and had similar patterns of pre-weaning weight gain. After weaning, however, the bodyweight differences rapidly disappeared, primarily because the females born in IUGR and median bodyweight categories grew to the same weight as the heavier birth weight animals within the first week after weaning. Thus, an inverse relationship between bodyweight at birth and post-weaning (week 3-4) bodyweight gain was also observed for females (Figure S1-B). However, because there was no difference in week 12 (adult) bodyweight based on bodyweight at birth, we did not continue to examine females from Block 1 and instead focused additional studies on males.

Regarding the postweaning growth rate in IUGR females, using the same postweaning growth rate cutoffs as for males (Figure 3), 33% of IUGR females showed below a 65% increase in bodyweight after weaning, while the remaining 67% of the IUGR females grew faster, similar to what was observed in males (data not shown).

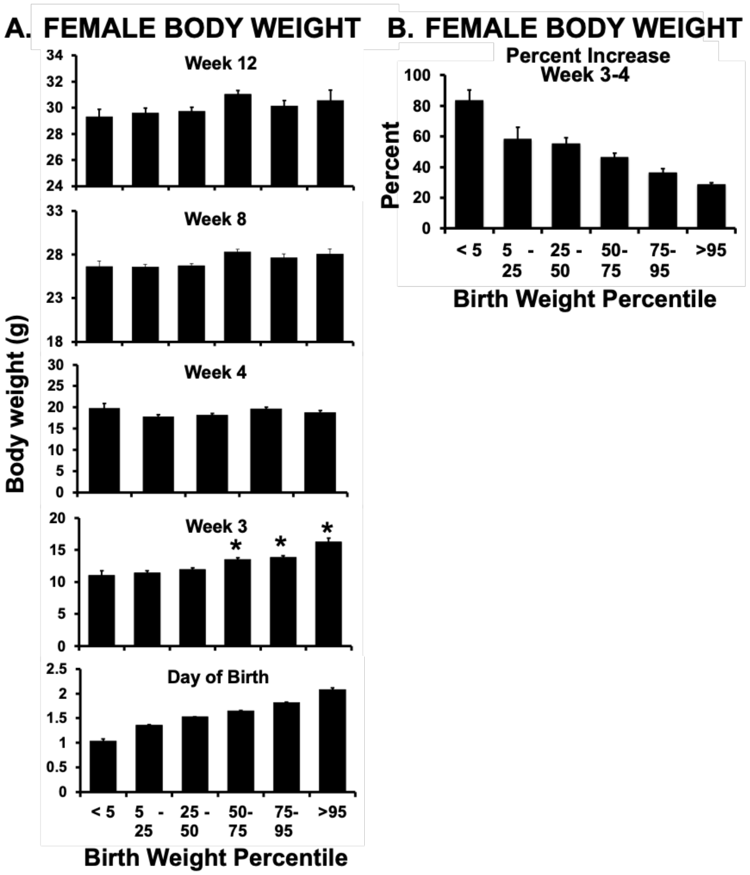

Figure S1. Birth weight and growth of female mice from Block 1. **A:** Body weights of female pups produced in 52 litters by Block 1 hemi-ovariectomized females based on birth weight categories, from the ≤5th percentile to ≥95th percentile. Beginning at postnatal week 3 (weaning), body weights were measured for all surviving female offspring until postnatal week 12. **B**: The percent increase in body weight for these females during the first week of free feeding after weaning (week 3-4). * $P < 0.05$ compared to females from the bottom 5th percentile (IUGR females). Data are mean±SEM.

Data for female offspring from Block 2 crowded uterus study

We conducted a GTT on female siblings of the Block 2 males, at the same age and using the same 4-hr fast procedure, and there were no significant effects on glucose tolerance based on analysis of the area under the glucose concentration time curve (AUC). These data are presented in Figure S2. Interestingly, the median birth weight females had significantly heavier gonadal fat pads relative to the IUGR-R(I-R) females (Figure S2-C).

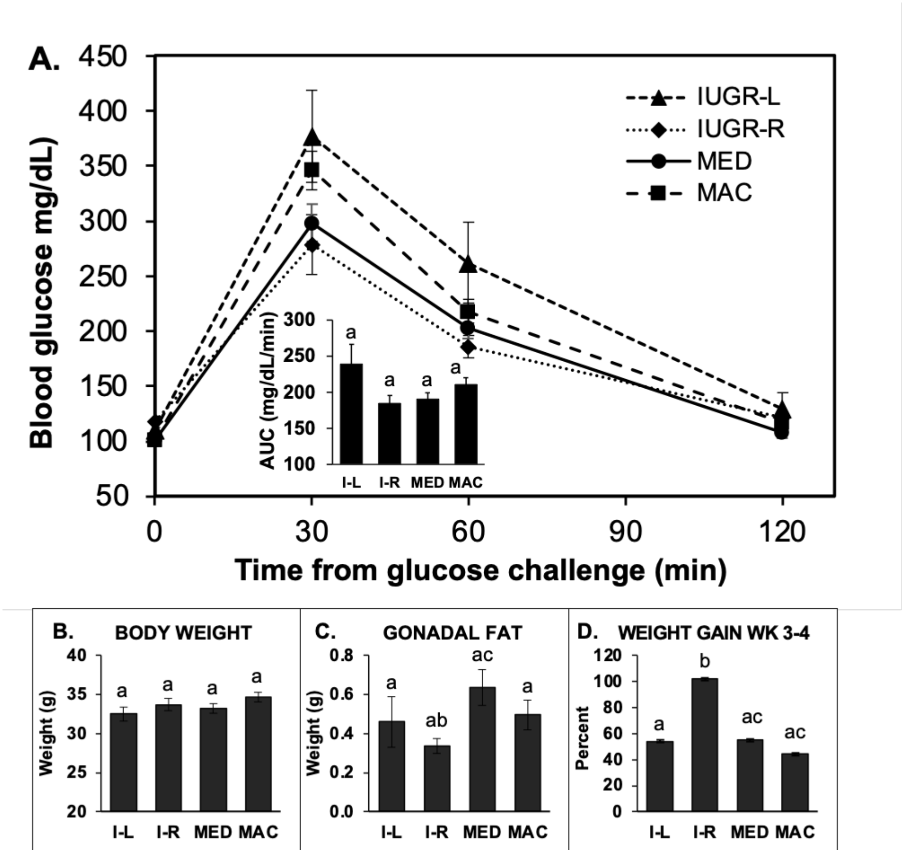

Figure S2. Glucose tolerance test in 4-hour fasted females. **A:** Glucose tolerance test for Block 2 female IUGR-L (I-L; n=9), IUGR-R (I-R; n=15), median (MED; n= 26) and macrosomic (MAC; n=22) females that underwent a 4-hr fast prior to glucose challenge. Inset: Area under the curve (AUC) for this test. No differences were seen in glucose tolerance between the four groups. **B**: Body weight at the time of tissue collection following the GTT. **C:** Gonadal fat weight, which was significantly greater in median females compared to IUGR-R females. **D:** The week 3-4 percent weight gain for these females. Groups with different letters are significantly different from each other ($P<0.05$); groups that share a letter in common are not statistically different. Values are mean±SEM.

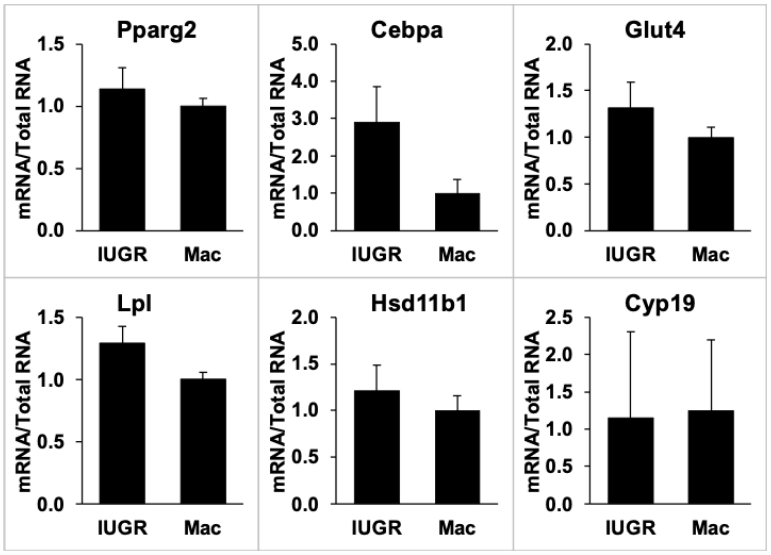

Figure S3. qPCR quantification of gonadal adipocyte gene expression in 14-hr fasted IUGR and macrosomic male mice. Values for IUGR animals are expressed relative to values for the macrosomic animals. n=5 per group. All values are mean±SEM.

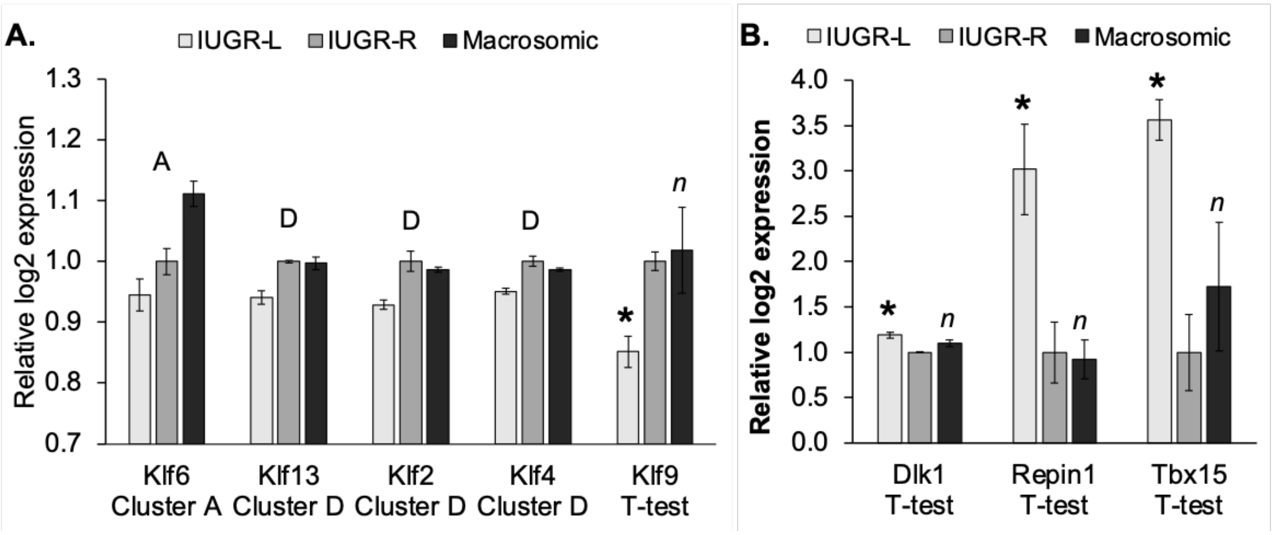

Figure S4. Expression of selected genes of interest from the microarray data set. **A:** Expression of differentially expressed Klf family genes; results are from Clustering analysis or T-test as indicated. T-test compares IUGR-L with IUGR-R only; the additional group is included to aid with data interpretation. **B:** Expression of other selected genes of interest; results are from T-tests comparing either Macrosomic with IUGR-R, or IUGR-L with IUGR-R; the additional group is included to aid with data interpretation. For both A and B, log2 expression values are shown relative to IUGR-R, and values are mean±SEM. A, IUGR-R and IUGR-L down-regulated relative to Macrosomic; D. IUGR-R and Macrosomic up-regulated relative to IUGR-L; *P<0.05 relative to IUGR-R; n = no statistical comparison between Macrosomic and IUGR-L samples. Values are mean ± SEM.

Table S1. Experimental design for studies with male offspring. Animals used in Block 1 (below) were selected from those shown in Figure 3; 2 IUGR-L animals and 1 median animal from those shown in Figure 3 were not used in these follow-up experiments.

**Breeding block**	**BLOCK 1 (52 litters)**	**BLOCK 2 (21 litters)**
**Sample sizes and data collected**	*Set 1*<br>IUGR-L n=6<br>IUGR-R n=10<br>Median n=16<br>Macrosomic n=16<br><br>• Body weights from weaning to week 12<br>• Glucose tolerance test 14-hour fast)<br>• Fat collection and fat weights (inguinal, renal, gonadal depots)<br>• Organ weights (heart, liver, kidney, spleen, testis, epididymis)<br>• Gonadal adipocyte counts, size distribution<br><br>*Set 2*<br>IUGR-L n=5<br>IUGR-R n=5<br>Macrosomic n=5<br><br>• Fat collection and fat weights (inguinal, renal, gonadal depots)<br>• Gonadal adipocyte counts, size distribution<br>• Gonadal fat qPCR<br>• Gonadal fat microarray	IUGR-L n=7<br>IUGR-R n=19<br>Median n=24<br>Macrosomic n=25<br><br>• Body weights, to establish birth and growth category<br>• Glucose tolerance test (4-hr fast)<br>• Gonadal fat pad weight

Table S2. Organ and body weights at time of collection in 14-hr fasted Block 1 males.

Tissue	IUGR-L	IUGR-R	Median	Macrosomic
Right testis (mg)	123.6 ± 7.7	136.9 ± 5.9	134.4 ± 5.5	137.7 ± 5.5
Right epididymis (mg)	50.9 ± 3.8	57.1 ± 2.9	58.8 ± 2.2 §	60.1 ± 2.7 §
Heart (mg)	200.2 ± 7.8 *	201.2 ± 6 *	206.3 ± 4.5 *	224 ± 5.6
Kidney (mg)	354.0 ± 28.8	353.7 ± 21.9 *	327.3 ± 16.7 ***	432.2 ± 20.5
Liver (g)	1.597 ± 0.109	1.641 ± 0.082	1.496± 0.063	1.746 ± 0.077
Spleen (mg)	87.3 ± 7.9	85.6 ± 6.0 *†	103.0 ± 4.6	108.7 ± 5.6
Body weight (g)	41.8 ± 0.6	39.4 ± 1.5	41.7 ± 0.7	47.0 ± 1.0
N	6	9	16	15

Values are based on LS means (± SEM) based on analysis of covariance with body weight as the covariate. * P<0.05, *** P<0.001 vs Macrosomic; † P<0.05 vs. Median; § P<0.09 vs. IUGR-L.

Table S3. Body weights and fat pad weights in 14-hr fasted and non-fasted Block 1 males. Values are mean±SEM. Statistical significance P < 0.05 indicated by different letters. a' and b' P < 0.1. *Inguinal fat n=4 for IUGR-L.

**14-hr FASTED:**	**IUGR-L**	**IUGR-R**	**MEDIAN**	**MACROSOMIC**
Inguinal fat (g)	0.126 ± 0.034	0.110 ± 0.016	0.163 ± 0.020	0.219 ± 0.026
Renal fat (g)	0.120 ± 0.050	0.120 ± 0.034	0.127 ± 0.017	0.271 ± 0.055
Gonadal fat (g)	0.323 ± 0.078^{a}	0.381 ± 0.087^{a}	0.462 ± 0.063$^{a,b'}$	0.678 ± 0.105^{b}
Total fat (g)	0.568 ± 0.160^{a}	0.611 ± 0.135^{a}	0.752 ± 0.098^{a}	1.168 ± 0.152^{b}
Body weight (g)	34.4 ± 1.3^{a}	38 ± 1$^{a'}$	37.2 ± 0.7$^{a'}$	44 ± 0.9^{b}
N	6	9	16	15
**NON-FASTED:**	**IUGR-L**	**IUGR-R**	**MACROSOMIC**	
Inguinal fat (g)*	0.152 ± 0.022^{a,b}	0.127 ± 0.015^{a}	0.202 ± 0.024^{b}	
Renal fat (g)	0.132 ± 0.025^{a}	0.163 ± 0.038^{a,b}	0.230 ± 0.032^{b}	
Gonadal fat (g)	0.351 ± 0.065^{a}	0.347 ± 0.101^{a}	0.574 ± 0.080$^{a'}$	
Total fat (g)	0.605 ± 0.093^{a}	0.637 ± 0.118^{a}	1.006 ± 0.125^{b}	
Body weight (g)	38.8 ± 1.2^{a}	39.7 ± 1.5^{a,b}	43.8 ± 1.7^{b}	
N	5	5	5	

Inguinal, renal and gonadal fat pad weights for 14-hr fasted males in were significantly correlated. Specifically, relative to the weight of the gonadal fat pads, renal fat pad weights were approximately 66% lower (r^2=0.880), and inguinal fat pads were 60% lower (r^2=0.652). Gonadal fat accounted for 58-61% of the total fat measured (summed weights of gonadal, inguinal and renal fat pads). Groups with different letters are significantly different from each other, P < 0.05. Values are mean±SEM.

Table S4. Growth and fat weights of the sub-group of 14-hr fasted males and non-fasted males used in qPCR analyses. Values are mean ± SEM. *P<0.05, **P<0.01, ***P<0.001 vs IUGR; †P<0.05, ††† P<0.001 vs. Median.

**Set 1: 14-hr fasted**	**IUGR**	**MACROSOMIC**
Weight gain week 3-4 (%)	110.5 ± 13.0	32.2 ± 5.0 **
Birth Weight (g)	1.16 ± 0.04	2.20 ± 0.05 ***
Week 3 weight (g)	12.4 ± 0.7	17.2 ± 0.6 **
Week 4 weight (g)	25.9 ± 0.8	22.7 ± 1.3
Week 12 weight (g)	39.2 ± 0.6	41.4 ± 1.1
Inguinal fat (mg)	0.112 ± 0.015	0.205 ± 0
Gonadal fat (mg)	0.302 ± 0.034	0.594 ± 0.1 *
Renal fat (mg)	0.093 ± 0.016	0.189 ± 0 *
Total Fat (mg)	0.507 ± 0.054	0.988 ± 0.2 *
Group N	5	5
**Set 2: non-fasted**	**IUGR**	**MACROSOMIC**
Weight gain week 3-4 (%)	101.0 ± 11.8	49.1 ± 8.5 **
Birth Weight (g)	1.28 ± 0.01	1.99 ± 0.01 ***
Week 3 weight (g)	10.5 ± 1.6	16.1 ± 1.0 **
Week 4 weight (g)	20.7 ± 2.6	24.1 ± 2.0
Week 12 weight (g)	36.1 ± 1.6	39 ± 1.6
Inguinal fat (mg)	0.121 ± 0.017	0.202 ± 0.024 *
Gonadal fat (mg)	0.406 ± 0.106	0.574 ± 0.08
Renal fat (mg)	0.133 ± 0.031	0.23 ± 0.032
Total Fat (mg)	0.660 ± 0.150	1.006 ± 0.125
Group N	4	5

Table S5. Growth and fat weights of animals used in Microarray analyses. Values are mean ± SEM. *P < 0.05, **P < 0.01, ***P < 0.001 vs IUGR-L; †P < 0.05, ††† P < 0.001 vs. macrosomic.

**MICROARRAY ANIMALS:**	**IUGR-L**	**IUGR-R**	**MACROSOMIC**
Weight gain week 3-4 (%)	34.7 ± 2.9	98.4 ± 16.1 *†	41.6 ± 5.1
Birth Weight (g)	1.32 ± 0.01	1.26 ± 0.00 **†††	2.00 ± 0.01 ***
Week 3 weight (g)	10.3 ± 1.3	11.3 ± 1.7	16.4 ± 1.2 *
Week 4 weight (g)	13.8 ± 1.4	21.8 ± 1.8 *	23.4 ± 2.4 *
Week 12 weight (g)	35.1 ± 2.2	39.0 ± 1.0	39.0 ± 2.0
Inguinal fat (mg)	0.132 ± 0.012	0.148 ± 0.011	0.191 ± 0.028 *p=0.085*
Gonadal fat (mg)	0.25 ± 0.032	0.427 ± 0.158	0.579 ± 0.103
Renal fat (mg)	0.093 ± 0.005	0.212 ± 0.044*	0.235 ± 0.04 *
Total Fat (mg)	0.474 ± 0.043	0.786 ± 0.131	1.005 ± 0.161 *
Group N	3	3	4

Table S6. Gene pathways manually identified as being potentially impacted by either prenatal or postweaning development. Drawing from results of both T-tests and cluster analysis, we identified multiple genes that were differentially expressed within these pathways. *Direction is effect on gene expression in first group named in Comparison column. Where (2) is indicated, two transcripts of the same gene met the criteria but only one is reported.

Pathway	Analysis source	Comparison	Direction*	Gene ID	Gene identifier
**Renin-angiotensin system**	T-test	IUGR-R v IUGR-L	Up	Cpa3	NM_007753
	T-test	IUGR-R v IUGR-L	Down	Mas1	NM_008552
	T-test	IUGR-R v IUGR-L	Down	Agtr2	NM_007429
Trend: heavy vs. light animals	T-test	IUGR-R v Mac	Down	Agtr2	NM_007429
	Cluster A	(IUGR-L+-IUGR-R) v Macro	Down	Ctsa	NP_032932
	Cluster C	(IUGR-R + Mac) v IUGR-L	Down	Atp6ap2 (2)	NM_027439
	Cluster D	(IUGR-R + Mac) v IUGR-L	Up	Thop1	NP_073144
	Cluster D	(IUGR-R + Mac) v IUGR-L	Up	Anpep	NP_032512
	Cluster D	(IUGR-R + Mac) v IUGR-L	Up	Nln	NP_083723
**Ppar signaling**	T-test	IUGR-R v Mac	Up	Apoa1 (2)	AI527359
	T-test	IUGR-R v Mac	Up	Apoc3	BC021776
Trend: rapid vs. slow-growing	T-test	IUGR-R v Mac	Up	Fabp2	NM_007980
	T-test	IUGR-R v IUGR-L	Down	Ubc	BI653033
	Cluster D	(IUGR-R + Mac) v IUGR-L	Up	Acaa1 (2)	NM_130864
**Adipocytokine signaling**	T-test	IUGR-R v IUGR-L	Up	Ptpn11	L08663
	T-test	IUGR-R v IUGR-L	Down	Mapk10	BE947490
	T-test	IUGR-R v IUGR-L	Down	Mapk8	BB184171
Trend: heavy vs. light animals	Cluster D	(IUGR-R + Mac) v IUGR-L	Up	Ikbkg (2)	NP_001028333
	Cluster D	(IUGR-R + Mac) v IUGR-L	Up	Tradd	NP_001129539
**Glycolysis and gluconeogenesis**	T-test	IUGR-R v IUGR-L	Up	Hk1	NM_010438
	T-test	IUGR-R v IUGR-L	Up	Bpgm	NM_007563
	T-test	IUGR-R v IUGR-L	Down	Adh7	NM_009626
Trend: heavy vs. light animals	Cluster C	(IUGR-R + Mac) v IUGR-L	Down	Gpi1	NM_008155
	Cluster E	(IUGR-L + IUGR-R) v Macro	Up	Eno3	NM_001136062
**Fatty acid elongation, fatty acid metabolism**	T-test	IUGR-R v IUGR-L	Down	Adh7	NM_009626
	T-test	IUGR-R v IUGR-L	Down	Mecr	BB310008
	T-test	IUGR-R v IUGR-L	Down	Elovl	BB829575
Trend: heavy vs. light animals	Cluster C	(IUGR-R + Mac) v IUGR-L	Down	Acat2	NM_009338
	Cluster D	(IUGR-R + Mac) v IUGR-L	Up	Acaa2	NM_177470
	Cluster D	(IUGR-R + Mac) v IUGR-L	Up	Adh5	NM_007410
**Type II diabetes mellitus**	T-test	IUGR-R v IUGR-L	Down	Mapk8	BB184171
	T-test	IUGR-R v IUGR-L	Down	Mapk10	BE947490
	T-test	IUGR-R v IUGR-L	Down	Cacna1a	BE943756
Trend: heavy vs. light animals	T-test	IUGR-R v IUGR-L	Down	Cacna1d	BB048682
	Cluster C	(IUGR-R + Mac) v IUGR-L	Down	Cacna1d	NM_001083616
	Cluster C	(IUGR-R + Mac) v IUGR-L	Down	Pik3r2	NM_008841

Table S7-A, Microarray data: T-test results Differentially expressed genes. All data are log-transformed. The T-test adjustment used is Benjamini and Hochberg correction. *Direction is for IUGR-R compared to other group (IUGR-L or Macrosomic.)

IUGR-L vs. IUGR-R

Gene Identifier	Gene ID	Direction*	p-value	adj. p-value
BB023525	547127	Down	0.0346	0.2602
BB125596	100038868	Down	0.0161	0.2105
AI256288	100040465	Down	0.0309	0.2546
AK017006	100043539	Down	0.0349	0.2602
BM211445	-	Down	0.0257	0.2425
BB198943	-	Down	0.0327	0.2564
BE634837	-	Down	0.0024	0.1322
BB312387	-	Down	0.0006	0.0870
AK021269	-	Down	0.0003	0.0844
BG066461	-	Down	0.0007	0.0870
BB200185	-	Down	0.0055	0.1553
AV106191	-	Down	0.0029	0.1330
AW240647	-	Down	0.0006	0.0870
AV209128	-	Down	0.0000	0.0568
BG069546	-	Down	0.0002	0.0784
AV161067	-	Down	0.0002	0.0784
BQ175333	-	Down	0.0060	0.1553
AK012219	-	Down	0.0010	0.0898
BE953598	-	Down	0.0000	0.0568
BG071941	-	Down	0.0262	0.2440
BG066492	-	Down	0.0015	0.1125
BB361965	-	Down	0.0067	0.1608
BB235011	-	Down	0.0112	0.1914
AV291496	-	Down	0.0422	0.2698
C79035	-	Down	0.0030	0.1330
BB799477	-	Down	0.0081	0.1668
BG066120	-	Down	0.0041	0.1386
BB307421	-	Down	0.0006	0.0870
AI666381	-	Down	0.0023	0.1322
BB235388	-	Down	0.0048	0.1489
AV264531	-	Down	0.0116	0.1928
AI448156	-	Down	0.0079	0.1661
BC016545	-	Down	0.0016	0.1148
BG066943	-	Down	0.0127	0.1959
BG063160	-	Down	0.0347	0.2602
AK011375	-	Down	0.0149	0.2045
BB724383	-	Down	0.0388	0.2645
BB311937	-	Down	0.0007	0.0870
BG074947	-	Down	0.0105	0.1881
AV279288	-	Down	0.0319	0.2564
BB349272	-	Down	0.0362	0.2628
BB273442	-	Down	0.0004	0.0844
BI465596	-	Down	0.0288	0.2506
AK015212	-	Down	0.0190	0.2199
BB393368	-	Down	0.0418	0.2695
X04603	-	Down	0.0091	0.1767
AV277330	-	Down	0.0051	0.1514
AK019269	-	Down	0.0049	0.1489
AA511261	-	Down	0.0287	0.2506
NM_053239	-	Down	0.0188	0.2199
AK016497	-	Down	0.0341	0.2595
AV110775	-	Down	0.0447	0.2731
BG069002	-	Down	0.0488	0.2788
BB164560	-	Down	0.0440	0.2731
BM221121	-	Down	0.0270	0.2462
C79752	-	Down	0.0417	0.2694
BE457624	-	Down	0.0385	0.2645
BE984840	-	Down	0.0377	0.2645
AK015582	-	Down	0.0032	0.1330
BB022219	-	Down	0.0001	0.0784
AK016521	-	Down	0.0271	0.2463
BB748724	-	Down	0.0264	0.2440
BB229543	-	Down	0.0354	0.2602
BB470919	-	Down	0.0414	0.2688
AK017963	-	Down	0.0001	0.0694
BB244079	-	Down	0.0167	0.2120
AK009494	-	Down	0.0468	0.2752
BB306048	-	Down	0.0484	0.2781
BB051684	-	Down	0.0437	0.2729
BB530192	-	Down	0.0169	0.2120
BB241583	-	Down	0.0421	0.2695
AV044095	-	Down	0.0457	0.2749
BB076411	-	Down	0.0241	0.2388
AI503156	-	Down	0.0144	0.2037
BB078690	-	Down	0.0233	0.2370
AA408425	-	Down	0.0256	0.2425
BQ175183	-	Down	0.0157	0.2093
BM116762	-	Down	0.0320	0.2564
BG063268	-	Down	0.0476	0.2760
BE956390	-	Down	0.0076	0.1650
BG070556	-	Down	0.0113	0.1917
BM247146	-	Down	0.0209	0.2271
BB283836	-	Down	0.0125	0.1951
BB335802	-	Down	0.0081	0.1668
BG071903	-	Down	0.0119	0.1951
C77955	-	Down	0.0113	0.1917
BB357975	-	Down	0.0351	0.2602
AV118421	-	Down	0.0431	0.2729
BG070052	-	Down	0.0179	0.2166
AV245300	-	Down	0.0084	0.1700
BB410295	-	Down	0.0268	0.2448
BM229080	-	Down	0.0363	0.2628
U01310	-	Down	0.0064	0.1570

Macrosomic vs. IUGR-R

Gene Identifier	Gene ID	Direction*	p-value	adj. p-value
BB377136	-	Down	0.0019	0.2387
BB127374	-	Down	0.0000	0.0145
M55219	-	Down	0.0181	0.3617
L38424	-	Down	0.0204	0.3617
AU022537	-	Down	0.0418	0.3617
AV230350	-	Down	0.0007	0.1927
BG069010	-	Down	0.0183	0.3617
BG064921	-	Down	0.0035	0.2705
NM_027028	-	Down	0.0365	0.3617
BB138441	-	Down	0.0404	0.3617
BB424956	-	Down	0.0026	0.2465
BB312387	-	Down	0.0023	0.2465
NM_053239	-	Down	0.0166	0.3617
BB477142	-	Down	0.0144	0.3617
BB083632	-	Down	0.0101	0.3617
BB366490	-	Down	0.0049	0.2916
AK020969	-	Down	0.0488	0.3617
BB667376	-	Down	0.0011	0.2293
AV090440	-	Down	0.0381	0.3617
BG076663	-	Down	0.0374	0.3617
BB822932	-	Down	0.0006	0.1927
BB240193	-	Down	0.0304	0.3617
AI666381	-	Down	0.0027	0.2465
BB038663	-	Down	0.0461	0.3617
BB473586	-	Down	0.0430	0.3617
BG069002	-	Down	0.0459	0.3617
BG066943	-	Down	0.0177	0.3617
AI849504	-	Down	0.0188	0.3617
AK017840	-	Down	0.0385	0.3617
BB829808	-	Down	0.0413	0.3617
BM934616	-	Down	0.0462	0.3617
AV097026	-	Down	0.0441	0.3617
BB009491	-	Down	0.0165	0.3617
BE947483	-	Down	0.0474	0.3617
AV266901	-	Down	0.0435	0.3617
AK016655	-	Down	0.0023	0.2465
BG067536	-	Down	0.0335	0.3617
AV085233	-	Down	0.0358	0.3617
BG071676	-	Down	0.0347	0.3617
BE990172	-	Down	0.0336	0.3617
BB164560	-	Down	0.0312	0.3617
AU022728	-	Down	0.0310	0.3617
AW553082	-	Down	0.0129	0.3617
BB470919	-	Down	0.0114	0.3617
BB335802	-	Down	0.0454	0.3617
AK017183	-	Down	0.0325	0.3617
AV041077	-	Down	0.0351	0.3617
BB309880	-	Down	0.0430	0.3617
BB522820	-	Down	0.0465	0.3617
NM_020500	-	Down	0.0191	0.3617
BE991564	-	Down	0.0253	0.3617
AV209796	-	Down	0.0106	0.3617
BG070409	-	Down	0.0210	0.3617
BQ175333	-	Down	0.0166	0.3617
BE986922	-	Down	0.0248	0.3617
AV260036	-	Down	0.0364	0.3617
BB241408	-	Down	0.0110	0.3617
BB078690	-	Down	0.0040	0.2723
BG075336	-	Down	0.0330	0.3617
AI174045	-	Down	0.0293	0.3617
AV106191	-	Down	0.0099	0.3617
AK017624	-	Down	0.0276	0.3617
AK015613	-	Down	0.0301	0.3617
BF152877	-	Down	0.0212	0.3617
AK020800	-	Down	0.0141	0.3617
AV204697	-	Down	0.0355	0.3617
AK012914	-	Down	0.0414	0.3617
AV208370	-	Down	0.0417	0.3617
BG069101	-	Down	0.0395	0.3617
AV316320	-	Down	0.0489	0.3617
AK018281	-	Down	0.0015	0.2387
BE944943	-	Down	0.0088	0.3617
BB209673	-	Down	0.0330	0.3617
BB124449	-	Down	0.0241	0.3617
BG065715	-	Down	0.0386	0.3617
AK006049	-	Down	0.0243	0.3617
BE951141	-	Down	0.0485	0.3617
AK013397	-	Down	0.0225	0.3617
AU024679	-	Down	0.0121	0.3617
AV210911	-	Down	0.0163	0.3617
AK015582	-	Down	0.0414	0.3617
BB400852	-	Down	0.0128	0.3617
AI853502	-	Down	0.0119	0.3617
BG067824	-	Down	0.0482	0.3617
BG066010	-	Down	0.0119	0.3617
BC003855	-	Down	0.0487	0.3617
BG072147	-	Down	0.0021	0.2426
BG070203	-	Down	0.0419	0.3617
NM_025503	-	Down	0.0392	0.3617
AK012864	-	Down	0.0460	0.3617
BG063156	-	Down	0.0397	0.3617
BB244079	-	Down	0.0406	0.3617
BI248587	-	Down	0.0134	0.3617

BG073400	-	Down	0.0168	0.2120
BB033554	-	Down	0.0343	0.2595
NM_023123	-	Down	0.0193	0.2207
BF162286	-	Down	0.0350	0.2602
AA511261	-	Down	0.0407	0.2680
BG066572	-	Down	0.0370	0.2637
AW543121	-	Down	0.0210	0.2271
AA020017	-	Down	0.0062	0.1570
AI851453	-	Down	0.0401	0.2679
AK015390	-	Down	0.0330	0.2574
BB252309	-	Down	0.0454	0.2741
AW552781	-	Down	0.0498	0.2804
AV153257	-	Down	0.0093	0.1767
BE948119	-	Down	0.0030	0.1330
AU022574	-	Down	0.0366	0.2634
AV090279	-	Down	0.0207	0.2265
AV275253	-	Down	0.0312	0.2546
BG076107	-	Down	0.0264	0.2440
AI427465	-	Down	0.0401	0.2679
AV326590	-	Down	0.0421	0.2695
BM939341	-	Down	0.0492	0.2799
AK006049	-	Down	0.0110	0.1910
AK013917	-	Down	0.0322	0.2564
AK017055	-	Down	0.0328	0.2564
BB524016	-	Down	0.0387	0.2645
AK014907	-	Down	0.0335	0.2593
AK007209	-	Down	0.0246	0.2400
AW492805	-	Down	0.0025	0.1322
BB154792	-	Down	0.0383	0.2645
BG072069	-	Down	0.0145	0.2038
AW122758	-	Down	0.0194	0.2214
BG066304	-	Down	0.0047	0.1482
BG066204	-	Down	0.0116	0.1928
BB234224	-	Down	0.0282	0.2501
BE945018	-	Down	0.0102	0.1881
AV089258	-	Down	0.0343	0.2595
BG074689	-	Down	0.0448	0.2734
BG066309	-	Down	0.0459	0.2749
BB227199	-	Down	0.0103	0.1881
BG068088	-	Down	0.0265	0.2440
BE944943	-	Down	0.0032	0.1330
NM_053265	-	Down	0.0489	0.2788
BG075144	-	Down	0.0495	0.2801
AU024679	-	Down	0.0213	0.2271
AV273436	-	Down	0.0274	0.2469
AW554716	-	Down	0.0247	0.2400
BG071710	-	Down	0.0415	0.2688
BB489500	-	Down	0.0009	0.0898
BI248587	-	Down	0.0257	0.2425
AI464135	-	Down	0.0464	0.2751
BE980371	-	Down	0.0261	0.2440
BB160042	-	Down	0.0354	0.2602
BB546734	-	Down	0.0122	0.1951
AU024501	-	Down	0.0390	0.2645
BB712583	-	Down	0.0089	0.1748
AK014341	-	Down	0.0274	0.2469
BG066533	-	Down	0.0187	0.2199
BG068164	-	Down	0.0390	0.2645
BB497102	-	Down	0.0444	0.2731
BB385572	-	Down	0.0286	0.2506
BG067308	-	Down	0.0450	0.2741
AK019498	-	Down	0.0113	0.1917
BM222742	-	Down	0.0071	0.1632
AV007867	-	Down	0.0372	0.2643
BG076089	-	Down	0.0021	0.1311
BB465155	-	Down	0.0264	0.2440
AI315336	-	Down	0.0297	0.2524
AK019561	-	Down	0.0232	0.2370
AK020548	-	Down	0.0142	0.2033
C76434	-	Down	0.0342	0.2595
BB387845	-	Down	0.0056	0.1553
BB555188	-	Down	0.0432	0.2729
AK010621	-	Down	0.0260	0.2439
BE985527	-	Down	0.0005	0.0844
AV261947	-	Down	0.0006	0.0870
BG068488	-	Down	0.0107	0.1896
BG071190	-	Down	0.0062	0.1570
BM238039	-	Down	0.0123	0.1951
BB380864	-	Down	0.0276	0.2471
BB050233	-	Down	0.0442	0.2731
AU022537	-	Down	0.0493	0.2799
BG067074	-	Down	0.0404	0.2680
AK017060	-	Down	0.0392	0.2649
BM877939	-	Down	0.0091	0.1767
BG073901	-	Down	0.0246	0.2400
BG074145	-	Down	0.0073	0.1635
BE945821	-	Down	0.0076	0.1648
C87349	-	Down	0.0213	0.2271
AK017183	-	Down	0.0159	0.2093
AK004807	-	Down	0.0030	0.1330
AV262303	-	Down	0.0312	0.2546
AK019579	-	Down	0.0183	0.2177
AV006589	-	Down	0.0407	0.2680
BB368905	-	Down	0.0164	0.2114
BB167641	-	Down	0.0252	0.2425
BB204368	-	Down	0.0122	0.1951
AU067808	-	Down	0.0446	0.2731
AK016251	-	Down	0.0434	0.2729
BM942479	-	Down	0.0364	0.2631
BM239519	-	Down	0.0185	0.2188
BB175647	-	Down	0.0077	0.1656
BM225255	-	Down	0.0292	0.2513
BM117148	-	Down	0.0089	0.3617
BB532219	-	Down	0.0358	0.3617
AK014614	-	Down	0.0016	0.2387
AV209602	-	Down	0.0218	0.3617
BE984540	-	Down	0.0342	0.3617
NM_021479	-	Down	0.0161	0.3617
AK017917	-	Down	0.0050	0.2917
AK015959	-	Down	0.0129	0.3617
AK016303	-	Down	0.0465	0.3617
BB181879	-	Down	0.0104	0.3617
BG065957	-	Down	0.0305	0.3617
BG067309	-	Down	0.0370	0.3617
BB283702	-	Down	0.0038	0.2723
BB040330	-	Down	0.0305	0.3617
BB385572	-	Down	0.0309	0.3617
BB560802	-	Down	0.0442	0.3617
AV046698	-	Down	0.0417	0.3617
AK014767	-	Down	0.0292	0.3617
AK015582	-	Down	0.0357	0.3617
C87473	-	Down	0.0358	0.3617
X17013	-	Down	0.0490	0.3617
AK021364	-	Down	0.0467	0.3617
BB308046	-	Down	0.0490	0.3617
BB310489	-	Down	0.0361	0.3617
BB391920	-	Down	0.0399	0.3617
AW491893	-	Down	0.0305	0.3617
BG071901	-	Down	0.0165	0.3617
BG066461	-	Down	0.0201	0.3617
BG073901	-	Down	0.0421	0.3617
AV271801	-	Down	0.0148	0.3617
BE686035	-	Down	0.0354	0.3617
AK015030	-	Down	0.0367	0.3617
BB548602	-	Down	0.0359	0.3617
BE956835	-	Down	0.0323	0.3617
BG073400	-	Down	0.0335	0.3617
AK016931	-	Down	0.0129	0.3617
BB230861	-	Down	0.0496	0.3617
BB469236	-	Down	0.0307	0.3617
AK018677	-	Down	0.0496	0.3617
AW492960	-	Down	0.0049	0.2916
BB528953	-	Down	0.0304	0.3617
AV381718	-	Down	0.0236	0.3617
BE988444	-	Down	0.0110	0.3617
C88061	-	Down	0.0037	0.2723
BB497755	-	Down	0.0478	0.3617
AK005564	-	Down	0.0467	0.3617
NM_008735	-	Down	0.0071	0.3464
BB308401	-	Down	0.0111	0.3617
BG068007	-	Down	0.0121	0.3617
BB531351	-	Down	0.0384	0.3617
AF027503	-	Down	0.0345	0.3617
BG069763	-	Down	0.0201	0.3617
BI499265	-	Down	0.0307	0.3617
AK014963	-	Down	0.0412	0.3617
AK009355	-	Down	0.0262	0.3617
AI849122	-	Down	0.0456	0.3617
BE957062	-	Down	0.0445	0.3617
AK015836	-	Down	0.0373	0.3617
AK005639	1700003H04Rik	Down	0.0412	0.3617
BF018978	1700007E06Rik	Down	0.0296	0.3617
AK005884	1700012A03Rik	Down	0.0375	0.3617
AK006213	1700021K02Rik	Down	0.0359	0.3617
AK018840	1700034E13Rik	Down	0.0119	0.3617
NM_028494	1700034I23Rik	Down	0.0219	0.3617
NM_026096	1700054O13Rik	Down	0.0287	0.3617
AK007082	1700095J03Rik	Down	0.0315	0.3617
BC019553	2210407C18Rik	Down	0.0090	0.3617
BB667186	4930412F15Rik	Down	0.0408	0.3617
AV255799	4930523C11Rik	Down	0.0040	0.2723
AV267411	4930526D03Rik	Down	0.0108	0.3617
AK016041	4930544O15Rik	Down	0.0138	0.3617
AK016218	4930564D02Rik	Down	0.0163	0.3617
AK016475	4931423N10Rik	Down	0.0410	0.3617
AK016488	4931429L15Rik	Down	0.0220	0.3617
AI789751	9030619P08Rik	Down	0.0321	0.3617
BB371633	9530059O14Rik	Down	0.0093	0.3617
BB262224	A830005F24Rik	Down	0.0089	0.3617
AJ457190	Abi3	Down	0.0478	0.3617
U38806	Adam2	Down	0.0440	0.3617
BB723712	Afp	Down	0.0088	0.3617
NM_007429	Agtr2	Down	0.0000	0.0424
AI852969	AI854703	Down	0.0097	0.3617
AI315673	Ankrd44	Down	0.0102	0.3617
BM248774	Arhgap5	Down	0.0034	0.2705
NM_008553	Ascl1	Down	0.0238	0.3617
BI696040	Atp1a1	Down	0.0114	0.3617
NM_134157	Atp6v1b1	Down	0.0065	0.3289
BB303874	Atp8a1	Down	0.0368	0.3617
BM945167	B3gat1	Down	0.0286	0.3617
AV328055	BC030500	Down	0.0469	0.3617
NM_022884	Bhmt2	Down	0.0095	0.3617
NM_019430	Cacng3	Down	0.0159	0.3617
BB333386	Cadm2	Down	0.0039	0.2723
BF660296	Camk4	Down	0.0275	0.3617
NM_030558	Car15	Down	0.0041	0.2723
AF407332	Catsper1	Down	0.0406	0.3617
BB385581	Cbl	Down	0.0331	0.3617
AF091432	Ccne2	Down	0.0080	0.3617
NM_009913	Ccr9	Down	0.0174	0.3617
AI894122	Cd82	Down	0.0077	0.3617
BE949427	Cdc37l1	Down	0.0457	0.3617
BB273513	Cdh8	Down	0.0300	0.3617

BB320816	-	Down	0.0068	0.1608
AK014767	-	Down	0.0377	0.2645
BB737833	-	Down	0.0489	0.2788
BB202185	-	Down	0.0052	0.1517
BB147330	-	Down	0.0145	0.2038
AK021364	-	Down	0.0328	0.2564
BM199355	-	Down	0.0471	0.2752
BM225255	-	Down	0.0461	0.2749
BB375259	-	Down	0.0109	0.1901
BB424956	-	Down	0.0434	0.2729
BG068241	-	Down	0.0123	0.1951
BM117148	-	Down	0.0384	0.2645
BM224192	-	Down	0.0402	0.2679
BG068396	-	Down	0.0188	0.2199
AK018281	-	Down	0.0459	0.2749
AI450773	-	Down	0.0093	0.1767
BB667885	-	Down	0.0410	0.2684
BB749667	-	Down	0.0289	0.2506
AK017498	-	Down	0.0421	0.2695
BG063165	-	Down	0.0212	0.2271
BM198834	-	Down	0.0381	0.2645
BB323133	-	Down	0.0305	0.2541
C88061	-	Down	0.0143	0.2037
BB531351	-	Down	0.0047	0.1482
AU043103	-	Down	0.0042	0.1392
BB025762	-	Down	0.0373	0.2643
BB204558	-	Down	0.0141	0.2030
BB188646	-	Down	0.0178	0.2166
AW048415	-	Down	0.0463	0.2751
BB209183	-	Down	0.0478	0.2768
BG075843	-	Down	0.0276	0.2469
AV327883	-	Down	0.0482	0.2780
BG066901	-	Down	0.0265	0.2440
BB528213	-	Down	0.0140	0.2030
C76358	-	Down	0.0051	0.1514
BB207470	-	Down	0.0388	0.2645
BB038556	-	Down	0.0002	0.0844
BM196689	-	Down	0.0255	0.2425
AW492692	-	Down	0.0069	0.1617
BB150912	-	Down	0.0452	0.2741
AI480624	-	Down	0.0050	0.1496
AV381718	-	Down	0.0364	0.2630
BB283805	-	Down	0.0131	0.1992
BB127498	-	Down	0.0110	0.1910
AW120636	-	Down	0.0353	0.2602
BB386165	-	Down	0.0380	0.2645
BG066333	-	Down	0.0207	0.2265
AI615965	-	Down	0.0357	0.2618
BE949326	-	Down	0.0349	0.2602
BB183984	-	Down	0.0078	0.1656
AK015547	-	Down	0.0419	0.2695
BB534600	-	Down	0.0322	0.2564
BI158554	-	Down	0.0146	0.2044
BF020502	-	Down	0.0060	0.1553
AI429425	-	Down	0.0150	0.2056
BB471224	-	Down	0.0076	0.1648
W91649	-	Down	0.0039	0.1383
AF071431	-	Down	0.0000	0.0568
BB165850	-	Down	0.0325	0.2564
AI464227	-	Down	0.0163	0.2111
BB662083	-	Down	0.0168	0.2120
BB224546	-	Down	0.0325	0.2564
BB381263	-	Down	0.0017	0.1196
BC024928	-	Down	0.0306	0.2541
C77957	-	Down	0.0083	0.1684
BM218863	-	Down	0.0358	0.2618
BB507994	-	Down	0.0021	0.1311
BB346879	-	Down	0.0489	0.2788
BG066303	-	Down	0.0406	0.2680
BB463760	-	Down	0.0310	0.2546
AA517023	-	Down	0.0104	0.1881
BB324138	-	Down	0.0050	0.1504
BB320171	-	Down	0.0235	0.2372
BB223643	-	Down	0.0286	0.2506
BB556191	-	Down	0.0346	0.2602
AV006589	-	Down	0.0155	0.2075
BE957062	-	Down	0.0069	0.1617
BG067629	-	Down	0.0078	0.1656
AK016932	-	Down	0.0457	0.2749
NM_030247	-	Down	0.0022	0.1311
BE995561	-	Down	0.0182	0.2166
BB525437	-	Down	0.0487	0.2788
NM_008735	-	Down	0.0323	0.2564
AW557641	-	Down	0.0231	0.2370
BM939280	-	Down	0.0131	0.1992
BQ175171	-	Down	0.0005	0.0844
BE992440	-	Down	0.0255	0.2425
AK017106	-	Down	0.0034	0.1330
BB751612	-	Down	0.0391	0.2645
AU043549	-	Down	0.0406	0.2680
BG067678	-	Down	0.0046	0.1477
BB534298	-	Down	0.0088	0.1739
BB246383	-	Down	0.0438	0.2731
AK005972	-	Down	0.0085	0.1707
BG064867	-	Down	0.0406	0.2680
BG070395	-	Down	0.0274	0.2469
BB484739	-	Down	0.0060	0.1553
AW124917	-	Down	0.0324	0.2564
BB271145	-	Down	0.0475	0.2757
BB084839	-	Down	0.0127	0.1951
BB219075	-	Down	0.0309	0.2546
AI427801	-	Down	0.0353	0.2602
X15351	Ceacam1	Down	0.0151	0.3617
BC019716	Cep350	Down	0.0341	0.3617
BB425924	Clint1	Down	0.0416	0.3617
U20265	Copg2as2	Down	0.0371	0.3617
AK003088	Cpa1	Down	0.0077	0.3617
U21729	Crhr2	Down	0.0282	0.3617
BG067914	Crygb	Down	0.0015	0.2387
BB148221	Csprs	Down	0.0421	0.3617
NM_033616	Csprs	Down	0.0298	0.3617
NM_033616	Csprs	Down	0.0343	0.3617
AK006130	Csrnp1	Down	0.0422	0.3617
AK005665	Cst13	Down	0.0497	0.3617
C86683	D6Mit97	Down	0.0170	0.3617
BE980110	Dact3	Down	0.0396	0.3617
NM_019728	Defb4	Down	0.0335	0.3617
BG295389	Dhcr24	Down	0.0208	0.3617
AF022078	Dlx6	Down	0.0009	0.2236
BE952491	Dnajb14	Down	0.0305	0.3617
AV161550	Dpysl3	Down	0.0008	0.2026
BB293562	Dtl	Down	0.0163	0.3617
BB539070	E130016E03Rik	Down	0.0171	0.3617
NM_007903	Edn3	Down	0.0044	0.2765
BB668107	EG218444	Down	0.0258	0.3617
BB807687	EG624855	Down	0.0313	0.3617
BF227830	Eif4g1	Down	0.0401	0.3617
NM_007972	F10	Down	0.0164	0.3617
NM_027828	Fam110c	Down	0.0014	0.2381
BG071043	Fbxl13	Down	0.0399	0.3617
BE947102	Fbxl7	Down	0.0148	0.3617
AF263536	Fgf23	Down	0.0433	0.3617
BI249549	Foxj2	Down	0.0397	0.3617
AB032770	Foxo4	Down	0.0020	0.2387
NM_008066	Gabra2	Down	0.0041	0.2723
BQ175863	Gabra5	Down	0.0144	0.3617
BB316100	Gabrg3	Down	0.0087	0.3617
NM_010275	Gdnf	Down	0.0161	0.3617
BB548169	Gm941	Down	0.0388	0.3617
BB354676	Gpr150	Down	0.0000	0.0424
NM_032399	Gpr87	Down	0.0151	0.3617
AK002633	Grhl2	Down	0.0023	0.2465
NM_008169	Grin1	Down	0.0043	0.2765
BB443417	Gucy1a2	Down	0.0464	0.3617
AV018723	H2-Aa	Down	0.0230	0.3617
BB786529	Hamp2	Down	0.0230	0.3617
BF451327	Hdhd2	Down	0.0174	0.3617
AV337579	Hes5	Down	0.0209	0.3617
BB486740	Hif3a	Down	0.0395	0.3617
AK005588	Hmgb4	Down	0.0175	0.3617
NM_008264	Hoxa13	Down	0.0041	0.2723
AK017731	Hspbap1	Down	0.0199	0.3617
BG066466	Iffo2	Down	0.0072	0.3488
BB794657	Igf2bp1	Down	0.0403	0.3617
NM_023670	Igf2bp3	Down	0.0297	0.3617
BC003727	Il1a	Down	0.0139	0.3617
BB042417	Il1r1	Down	0.0328	0.3617
AJ277831	Il1rapl2	Down	0.0073	0.3506
BM220651	Irak4	Down	0.0123	0.3617
BG069095	Irf8	Down	0.0365	0.3617
AK020772	Itk	Down	0.0066	0.3305
BE981282	Kbtbd7	Down	0.0087	0.3617
BF322939	Kcnk12	Down	0.0427	0.3617
NM_133235	Khdrbs2	Down	0.0404	0.3617
NM_019974	Klk11	Down	0.0232	0.3617
NM_008457	Klk1b8	Down	0.0392	0.3617
AF288377	Klra1	Down	0.0448	0.3617
AF288379	Klra7	Down	0.0065	0.3289
NM_008476	Krt6a	Down	0.0114	0.3617
BE987427	Lpgat1	Down	0.0013	0.2346
AK017594	Lrfn2	Down	0.0316	0.3617
BB437173	Lrp6	Down	0.0250	0.3617
AI267139	Lrrc19	Down	0.0134	0.3617
AI326034	Lyzl6	Down	0.0455	0.3617
AK008018	Mtap7	Down	0.0142	0.3617
AJ293626	Myh1	Down	0.0427	0.3617
AK002312	Myl3	Down	0.0385	0.3617
AK010332	Nanog	Down	0.0394	0.3617
BF019797	Nbeal2	Down	0.0238	0.3617
AK017458	Neto2	Down	0.0477	0.3617
AF289078	Nfatc2	Down	0.0046	0.2863
BC025455	Nipbl	Down	0.0039	0.2723
AK018248	Nkain2	Down	0.0378	0.3617
NM_008702	Nlk	Down	0.0309	0.3617
BB204913	Nlrc3	Down	0.0320	0.3617
D32210	Notch2	Down	0.0034	0.2705
NM_010933	Nppc	Down	0.0472	0.3617
NM_016708	Npy5r	Down	0.0033	0.2696
BB322941	Nr4a2	Down	0.0307	0.3617
NM_020610	Nrip3	Down	0.0221	0.3617
BB265776	Pcdha6	Down	0.0199	0.3617
NM_009981	Pcyt1a	Down	0.0025	0.2465
AK020261	Pdx1	Down	0.0409	0.3617
BB315077	Pex5l	Down	0.0469	0.3617
AK015656	Pick1	Down	0.0483	0.3617
BB490622	Plekha5	Down	0.0363	0.3617
AF408412	Pof1b	Down	0.0433	0.3617
AW240531	Polr3a	Down	0.0006	0.1927
AW455905	Ppp2r2c	Down	0.0187	0.3617
BM250725	Prl2c5	Down	0.0003	0.1362
AV225384	Prl7a1	Down	0.0059	0.3209
AF182065	Prok2	Down	0.0375	0.3617
AW060288	Qk	Down	0.0292	0.3617
BM214169	Rab8b	Down	0.0490	0.3617

BB466083	-	Down	0.0338	0.2594
BB082509	-	Down	0.0324	0.2564
BM120569	-	Down	0.0187	0.2199
AW213766	-	Down	0.0306	0.2541
BB356354	-	Down	0.0263	0.2440
AK015242	-	Down	0.0449	0.2738
BE627942	-	Down	0.0003	0.0844
AK018213	-	Down	0.0071	0.1632
AI505326	-	Down	0.0455	0.2747
BB247338	-	Down	0.0175	0.2163
AW413496	-	Down	0.0004	0.0844
BB702472	-	Down	0.0235	0.2372
AV347837	-	Down	0.0399	0.2678
BB201888	-	Down	0.0025	0.1330
BB546069	-	Down	0.0465	0.2751
AK014957	-	Down	0.0089	0.1749
BB205102	-	Down	0.0037	0.1349
BB102433	-	Down	0.0014	0.1125
BB328387	-	Down	0.0194	0.2213
NM_008735	-	Down	0.0026	0.1330
BE989344	-	Down	0.0470	0.2752
BB233502	-	Down	0.0004	0.0844
BE691677	-	Down	0.0181	0.2166
BB076855	-	Down	0.0193	0.2207
BB230894	-	Down	0.0077	0.1656
BB466114	-	Down	0.0388	0.2645
BG070025	-	Down	0.0142	0.2033
BB518847	-	Down	0.0155	0.2077
AU022339	-	Down	0.0019	0.1256
AK015383	-	Down	0.0233	0.2370
AI851474	-	Down	0.0285	0.2506
BF318475	-	Down	0.0203	0.2265
BB277721	-	Down	0.0255	0.2425
BB735036	-	Down	0.0012	0.1000
BE686035	-	Down	0.0282	0.2501
AK019900	-	Down	0.0417	0.2694
BB467267	-	Down	0.0216	0.2285
BB604994	1110021J02Rik	Down	0.0289	0.2506
AV023018	1110028C15Rik	Down	0.0094	0.1788
BC022950	1600029D21Rik	Down	0.0190	0.2199
BM214338	1700019G17Rik	Down	0.0211	0.2271
NM_023064	1700021K02Rik	Down	0.0425	0.2711
AK006428	1700027J07Rik	Down	0.0441	0.2731
AK006746	1700049K14Rik	Down	0.0407	0.2680
AV258486	1700073E17Rik	Down	0.0488	0.2788
AK007082	1700095J03Rik	Down	0.0088	0.1748
AK007241	1700122O11Rik	Down	0.0011	0.0919
AK010086	2310067E19Rik	Down	0.0306	0.2541
AK010362	2410003J06Rik	Down	0.0460	0.2749
BF467494	2810408A11Rik	Down	0.0444	0.2731
AK017673	2810417H13Rik	Down	0.0047	0.1482
AK014549	4632404H12Rik	Down	0.0075	0.1643
AV278097	4921528O07Rik	Down	0.0442	0.2731
AV255799	4930523C11Rik	Down	0.0031	0.1330
AK016248	4930568D16Rik	Down	0.0346	0.2602
AV381533	5830403L16Rik	Down	0.0211	0.2271
AV376276	6330416L07Rik	Down	0.0064	0.1570
AV133931	6720463M24Rik	Down	0.0105	0.1881
BB079733	9330159F19Rik	Down	0.0039	0.1383
BB177150	A230065H16Rik	Down	0.0336	0.2593
BM243643	A430107P09Rik	Down	0.0376	0.2645
BB262224	A830005F24Rik	Down	0.0334	0.2589
AK020834	A930009A15Rik	Down	0.0242	0.2388
NM_021370	Accn5	Down	0.0354	0.2602
BE573407	Acer3	Down	0.0165	0.2119
NM_009599	Ache	Down	0.0025	0.1330
NM_009626	Adh7	Down	0.0011	0.0935
NM_007423	Afp	Down	0.0220	0.2315
NM_007429	Agtr2	Down	0.0001	0.0694
U39200	Alox12e	Down	0.0465	0.2751
AV267870	Als2cr11	Down	0.0228	0.2364
NM_009669	Amy2	Down	0.0055	0.1553
BM220087	Ank3	Down	0.0491	0.2793
AI315673	Ankrd44	Down	0.0032	0.1330
AW240637	Anxa2	Down	0.0072	0.1635
AW493372	Ap2b1	Down	0.0447	0.2731
NM_020563	Apbh	Down	0.0436	0.2729
AF104416	Aqp3	Down	0.0383	0.2645
BM248774	Arhgap5	Down	0.0126	0.1951
AV233793	Arnt	Down	0.0139	0.2030
AV233423	Asb10	Down	0.0063	0.1570
NM_025758	Asb17	Down	0.0233	0.2370
C78762	Atp5a1	Down	0.0001	0.0694
AK021146	Atp5sl	Down	0.0390	0.2645
NM_134157	Atp6v1b1	Down	0.0167	0.2120
BB825830	Atrx	Down	0.0125	0.1951
BB667330	B230339M05Rik	Down	0.0387	0.2645
BB424673	B3gat1	Down	0.0460	0.2749
BM945167	B3gat1	Down	0.0197	0.2225
NM_007519	Baat	Down	0.0162	0.2111
BG069765	Baz2b	Down	0.0293	0.2516
NM_022884	Bhmt2	Down	0.0228	0.2364
BB658793	Bicd1	Down	0.0189	0.2199
AK016125	Boll	Down	0.0288	0.2506
BE951353	Btbd17	Down	0.0301	0.2537
NM_009773	Bub1b	Down	0.0153	0.2061
BE981290	C130039O16Rik	Down	0.0010	0.0898
BI964419	C530008M17Rik	Down	0.0106	0.1889
AF419324	Cabp7	Down	0.0178	0.2166
BE943756	Cacna1a	Down	0.0472	0.2752
BB048682	Cacna1d	Down	0.0019	0.1275
BB333386	Cadm2	Down	0.0036	0.1349

BB274058	Rgs6	Down	0.0404	0.3617
AV047988	Rhobtb3	Down	0.0349	0.3617
AV269723	Rhox13	Down	0.0011	0.2293
BM210473	Rhox5	Down	0.0366	0.3617
AF155141	Rp1	Down	0.0017	0.2387
BC026459	RP23-433P19.11	Down	0.0496	0.3617
AW489945	Rundc3a	Down	0.0093	0.3617
NM_013650	S100a8	Down	0.0372	0.3617
NM_011315	Saa3	Down	0.0196	0.3617
BG064756	Sall4	Down	0.0033	0.2696
AV259481	Sdk2	Down	0.0363	0.3617
AB021291	Sema4f	Down	0.0282	0.3617
BB471743	Setd8	Down	0.0307	0.3617
BF468072	Slc12a5	Down	0.0328	0.3617
C85957	Slc35c2	Down	0.0277	0.3617
BG066440	Slco6c1	Down	0.0009	0.2180
BB227643	Slfn10	Down	0.0302	0.3617
BI684556	Smc2	Down	0.0166	0.3617
AK010671	Snrpn	Down	0.0327	0.3617
NM_009237	Sox3	Down	0.0250	0.3617
BG070168	Spry4	Down	0.0068	0.3366
NM_030237	Spz1	Down	0.0012	0.2293
X91043	Stom	Down	0.0354	0.3617
BB352105	Tbc1d16	Down	0.0426	0.3617
M87849	Tcrb-V13	Down	0.0242	0.3617
AK016186	Tmem159	Down	0.0298	0.3617
BF138523	Tmprss13	Down	0.0440	0.3617
NM_009407	Tnp1	Down	0.0392	0.3617
BC025631	Tnrc18	Down	0.0114	0.3617
AY034576	Tpbpb	Down	0.0321	0.3617
AW554765	Tpr	Down	0.0041	0.2723
NM_011635	Trap1a	Down	0.0126	0.3617
AF223415	Trdn	Down	0.0147	0.3617
AF223417	Trdn	Down	0.0276	0.3617
BB762957	Tyrp1	Down	0.0286	0.3617
AK003750	Ucma	Down	0.0000	0.0424
BC022133	Uroc1	Down	0.0434	0.3617
NM_053225	V1rb1	Down	0.0147	0.3617
BB551556	Vtcn1	Down	0.0266	0.3617
AW123067	Wapal	Down	0.0257	0.3617
BG067702	Wdfy4	Down	0.0294	0.3617
NM_009521	Wnt3	Down	0.0260	0.3617
NM_009522	Wnt3a	Down	0.0446	0.3617
BI102531	Wwp1	Down	0.0032	0.2696
AW488774	Zfp287	Down	0.0207	0.3617
AF290196	Zfp352	Down	0.0433	0.3617
AA682050	100042198	Up	0.0263	0.3617
AK015416	100042722	Up	0.0155	0.3617
BG069060	-	Up	0.0002	0.1221
AV298153	-	Up	0.0001	0.0947
BB498027	-	Up	0.0013	0.2346
BE947764	-	Up	0.0293	0.3617
AK015008	-	Up	0.0200	0.3617
BC010208	-	Up	0.0042	0.2765
AW046902	-	Up	0.0000	0.0424
C77533	-	Up	0.0049	0.2916
AI746421	-	Up	0.0058	0.3178
BB189091	-	Up	0.0046	0.2863
BB198406	-	Up	0.0386	0.3617
BG068057	-	Up	0.0093	0.3617
AK018062	-	Up	0.0177	0.3617
AI504240	-	Up	0.0165	0.3617
BB533980	-	Up	0.0487	0.3617
BE630431	-	Up	0.0251	0.3617
BG067109	-	Up	0.0476	0.3617
BE380915	-	Up	0.0002	0.1221
AK018489	-	Up	0.0028	0.2516
BG068598	-	Up	0.0127	0.3617
BG069718	-	Up	0.0423	0.3617
BB337425	-	Up	0.0216	0.3617
BB247020	-	Up	0.0190	0.3617
BB088844	-	Up	0.0061	0.3217
C80868	-	Up	0.0026	0.2465
BB130655	-	Up	0.0150	0.3617
BG064109	-	Up	0.0346	0.3617
BC010480	-	Up	0.0050	0.2916
AK009013	-	Up	0.0047	0.2897
BB137915	-	Up	0.0253	0.3617
BB191623	-	Up	0.0040	0.2723
BB125781	-	Up	0.0494	0.3617
BC005570	-	Up	0.0414	0.3617
BB304800	-	Up	0.0313	0.3617
BI872156	-	Up	0.0179	0.3617
AI551151	-	Up	0.0293	0.3617
BG060698	-	Up	0.0275	0.3617
BB026554	-	Up	0.0030	0.2645
AK010967	-	Up	0.0354	0.3617
AK016155	-	Up	0.0206	0.3617
BE851943	-	Up	0.0007	0.1927
BG085035	-	Up	0.0352	0.3617
BG072280	-	Up	0.0471	0.3617
AK006313	-	Up	0.0088	0.3617
BG076521	-	Up	0.0268	0.3617
BB041180	-	Up	0.0168	0.3617
M27608	-	Up	0.0488	0.3617
AK014616	-	Up	0.0372	0.3617
BM239853	-	Up	0.0061	0.3217
BG071002	-	Up	0.0005	0.1927
BG067710	-	Up	0.0096	0.3617
BF456247	-	Up	0.0135	0.3617
BB546771	-	Up	0.0418	0.3617
BB525809	-	Up	0.0341	0.3617

AW557885	Calb1	Down	0.0029	0.1330
BC017646	Calb2	Down	0.0289	0.2506
BF660296	Camk4	Down	0.0151	0.2061
AF127766	Capn3	Down	0.0117	0.1936
AK014839	Ccdc39	Down	0.0074	0.1636
AF091432	Ccne2	Down	0.0238	0.2386
AJ131357	Ccr9	Down	0.0258	0.2433
BB543291	Ccrl1	Down	0.0046	0.1466
L24495	Cd27	Down	0.0242	0.2388
NM_007650	Cd5	Down	0.0294	0.2516
BB154331	Cd8a	Down	0.0221	0.2323
BB483759	Cd93	Down	0.0105	0.1881
BB041150	Cdc20	Down	0.0436	0.2729
BG143419	Cdc45l	Down	0.0386	0.2645
BB273513	Cdh8	Down	0.0289	0.2506
BB191966	Cdk5rap2	Down	0.0107	0.1896
BB371618	Cdkn2aip	Down	0.0072	0.1634
BB546429	Chka	Down	0.0256	0.2425
NM_007697	Chl1	Down	0.0354	0.2602
BB748443	Chrna4	Down	0.0349	0.2602
BE627927	Cldnd1	Down	0.0013	0.1043
BB425924	Clint1	Down	0.0068	0.1608
NM_025469	Clps	Down	0.0230	0.2370
BB207504	Cmah	Down	0.0474	0.2757
BC006866	Cntd1	Down	0.0111	0.1912
AK010095	Col22a1	Down	0.0265	0.2440
BB631643	Colq	Down	0.0312	0.2546
BB283617	Cops3	Down	0.0121	0.1951
BG067914	Crygb	Down	0.0059	0.1553
NM_025583	Ctrb1	Down	0.0003	0.0844
NM_021445	Cts6	Down	0.0230	0.2370
BC012653	Cx3cr1	Down	0.0342	0.2595
NM_133657	Cyp2a12	Down	0.0137	0.2028
BF783609	Cyp2j5	Down	0.0187	0.2199
AK021319	D530049N12Rik	Down	0.0465	0.2751
BB136172	Depdc1b	Down	0.0426	0.2718
AW554652	Diap1	Down	0.0409	0.2684
NM_010052	Dlk1	Down	0.0058	0.1553
AF022078	Dlx6	Down	0.0032	0.1330
AV129500	Dnajc1	Down	0.0489	0.2788
C77692	Dpm1	Down	0.0313	0.2546
AV161550	Dpysl3	Down	0.0059	0.1553
NM_031174	Dscam	Down	0.0243	0.2388
BB293562	Dtl	Down	0.0121	0.1951
AI509011	Dzip1	Down	0.0205	0.2265
BM231657	Dzip3	Down	0.0070	0.1625
BM936291	Dzip3	Down	0.0018	0.1211
BB536333	E030049G20Rik	Down	0.0087	0.1739
BB539070	E130016E03Rik	Down	0.0167	0.2120
BB426176	E130307A14Rik	Down	0.0355	0.2602
AV285272	E330016A19Rik	Down	0.0056	0.1553
BB356446	Efr3b	Down	0.0380	0.2645
BB668107	EG218444	Down	0.0256	0.2425
AI504688	EG240038	Down	0.0317	0.2564
BC028643	EG245174	Down	0.0176	0.2163
BB807687	EG624855	Down	0.0252	0.2425
BF463689	EG668525	Down	0.0401	0.2679
BI439550	Ela3	Down	0.0052	0.1515
AV060902	Ela3	Down	0.0282	0.2501
BB829575	Elovl4	Down	0.0112	0.1914
AK010698	ENSMUSG00000074075	Down	0.0435	0.2729
NM_053103	Entpd7	Down	0.0203	0.2265
X79084	Epha7	Down	0.0239	0.2386
NM_133245	Eraf	Down	0.0037	0.1349
BB131270	Fam13c	Down	0.0005	0.0844
AI480750	Fam54b	Down	0.0010	0.0898
BB499286	Fancm	Down	0.0461	0.2749
NM_010177	Fasl	Down	0.0336	0.2593
AW048300	Fat2	Down	0.0406	0.2680
BM120467	Fat3	Down	0.0304	0.2541
BE947102	Fbxl7	Down	0.0126	0.1951
AK011118	Fgb	Down	0.0300	0.2537
M92416	Fgf6	Down	0.0151	0.2061
AV283456	Fhl4	Down	0.0041	0.1392
BB318003	Fibcd1	Down	0.0015	0.1125
AK021261	Fndc3b	Down	0.0443	0.2731
AW557298	Fnip1	Down	0.0000	0.0123
BB662927	Foxd1	Down	0.0291	0.2513
BQ175863	Gabra5	Down	0.0305	0.2541
AF276754	Gcg	Down	0.0228	0.2364
BC018297	Gcnt3	Down	0.0066	0.1603
AU017649	Gdap1	Down	0.0430	0.2729
BB824003	Gemin5	Down	0.0125	0.1951
BB183081	Gfap	Down	0.0234	0.2370
NM_010278	Gfi1	Down	0.0324	0.2564
NM_080438	Glra3	Down	0.0489	0.2789
BB535531	Gpr146	Down	0.0024	0.1322
AK002633	Grhl2	Down	0.0163	0.2114
AV231424	Grhl3	Down	0.0042	0.1404
X03051	H2-D1	Down	0.0433	0.2729
M16810	H2-D1	Down	0.0420	0.2695
X03052	H2-D1	Down	0.0443	0.2731
M16810	H2-D1	Down	0.0259	0.2434
NM_010398	H2-D1	Down	0.0117	0.1936
U96752	H2-K1	Down	0.0383	0.2645
BB786529	Hamp2	Down	0.0049	0.1489
BB803143	Hcrtr1	Down	0.0105	0.1881
C80147	Hdgf	Down	0.0083	0.1684
NM_053149	Hemgn	Down	0.0162	0.2111
AK019671	Hhipl2	Down	0.0390	0.2645
BB486740	Hif3a	Down	0.0362	0.2628
AK017366	-	Up	0.0081	0.3617
AW121350	-	Up	0.0157	0.3617
AK020414	-	Up	0.0079	0.3617
AB001737	-	Up	0.0485	0.3617
BG070662	-	Up	0.0156	0.3617
BI732990	-	Up	0.0433	0.3617
BG074029	-	Up	0.0471	0.3617
AI646399	-	Up	0.0025	0.2465
BB215043	-	Up	0.0081	0.3617
BB041802	-	Up	0.0418	0.3617
AV166476	-	Up	0.0448	0.3617
AA619999	-	Up	0.0325	0.3617
BG064321	-	Up	0.0102	0.3617
AK013759	-	Up	0.0097	0.3617
BB614236	-	Up	0.0220	0.3617
BM234751	-	Up	0.0424	0.3617
BB388293	-	Up	0.0465	0.3617
BB787201	-	Up	0.0393	0.3617
BG072474	-	Up	0.0350	0.3617
BG076300	-	Up	0.0379	0.3617
AK007172	-	Up	0.0228	0.3617
BB082380	-	Up	0.0397	0.3617
AK017568	-	Up	0.0043	0.2765
AK020658	-	Up	0.0253	0.3617
BE948587	-	Up	0.0462	0.3617
AV228155	-	Up	0.0422	0.3617
BG075663	-	Up	0.0062	0.3239
BB279619	-	Up	0.0216	0.3617
AK014899	-	Up	0.0197	0.3617
BF581125	-	Up	0.0468	0.3617
BG066316	-	Up	0.0482	0.3617
BC002257	-	Up	0.0206	0.3617
BG070525	-	Up	0.0455	0.3617
AK020016	-	Up	0.0422	0.3617
BF456117	-	Up	0.0489	0.3617
AV274143	-	Up	0.0024	0.2465
BG069104	-	Up	0.0463	0.3617
NM_019509	-	Up	0.0292	0.3617
AU022864	-	Up	0.0492	0.3617
AV274889	-	Up	0.0180	0.3617
AK017949	-	Up	0.0175	0.3617
BQ176688	-	Up	0.0060	0.3216
BB055532	-	Up	0.0403	0.3617
BG066485	-	Up	0.0213	0.3617
C87101	-	Up	0.0313	0.3617
AK015368	-	Up	0.0401	0.3617
AV352500	-	Up	0.0311	0.3617
AK016792	-	Up	0.0481	0.3617
BM121794	-	Up	0.0274	0.3617
AV324577	-	Up	0.0282	0.3617
AI551328	-	Up	0.0386	0.3617
AK016327	-	Up	0.0359	0.3617
BB429200	-	Up	0.0485	0.3617
BM936732	-	Up	0.0094	0.3617
AW125829	-	Up	0.0384	0.3617
AV169581	-	Up	0.0055	0.3129
BF471100	-	Up	0.0295	0.3617
AK021065	-	Up	0.0364	0.3617
BG075835	-	Up	0.0214	0.3617
BB346592	-	Up	0.0469	0.3617
AI551889	-	Up	0.0348	0.3617
NM_023450	-	Up	0.0447	0.3617
AK011368	-	Up	0.0432	0.3617
BM114149	-	Up	0.0275	0.3617
NM_026351	-	Up	0.0330	0.3617
AK016607	-	Up	0.0342	0.3617
NM_016793	-	Up	0.0421	0.3617
BG071123	-	Up	0.0096	0.3617
BG072095	-	Up	0.0224	0.3617
BG795494	-	Up	0.0418	0.3617
NM_025376	1110002H13Rik	Up	0.0390	0.3617
AK004439	1190002A17Rik	Up	0.0212	0.3617
AK005650	1700003M02Rik	Up	0.0087	0.3617
BB546123	1700008O03Rik	Up	0.0159	0.3617
AV044666	1700018G05Rik	Up	0.0364	0.3617
AK006434	1700027L20Rik	Up	0.0002	0.1221
BB256666	1700041C02Rik	Up	0.0328	0.3617
AK007013	1700086D15Rik	Up	0.0468	0.3617
BI526033	1700086L19Rik	Up	0.0169	0.3617
AK018926	1700094C09Rik	Up	0.0381	0.3617
AK010406	2410004P03Rik	Up	0.0025	0.2465
BC020160	4833422F24Rik	Up	0.0028	0.2516
AK015309	4930434E21Rik	Up	0.0312	0.3617
BC024760	4930529M08Rik	Up	0.0411	0.3617
NM_133689	4930579J09Rik	Up	0.0113	0.3617
AI414902	5033428B15Rik	Up	0.0033	0.2696
AK017258	5430402E10Rik	Up	0.0345	0.3617
AV258949	6430537H07Rik	Up	0.0368	0.3617
AV342060	6430571L13Rik	Up	0.0436	0.3617
BB751940	9130014G24Rik	Up	0.0437	0.3617
AK020284	9130221D24Rik	Up	0.0063	0.3249
AF319955	9530002B09Rik	Up	0.0491	0.3617
AV255758	A330021E22Rik	Up	0.0498	0.3617
NM_023383	Aadac	Up	0.0206	0.3617
NM_028514	Actrt1	Up	0.0328	0.3617
BB171354	Adcyap1	Up	0.0357	0.3617
AI323434	Adcyap1	Up	0.0041	0.2723
BB155250	Agps	Up	0.0318	0.3617
BC023499	Agr3	Up	0.0322	0.3617
AK006382	Akap13	Up	0.0153	0.3617
NM_033603	Amn	Up	0.0127	0.3617

AV127319	Hist2h2be	Down	0.0009	0.0898
BB281327	Hkdc1	Down	0.0484	0.2781
X64550	Hmmr	Down	0.0098	0.1835
BB552443	Hoxa10	Down	0.0033	0.1330
AV116521	Hoxa11as	Down	0.0396	0.2666
NM_008264	Hoxa13	Down	0.0036	0.1349
NM_008275	Hoxd13	Down	0.0119	0.1951
NM_008294	Hsd3b4	Down	0.0175	0.2161
NM_013821	Hsd3b6	Down	0.0493	0.2799
NM_019960	Hspb3	Down	0.0064	0.1570
BB446602	Igdcc3	Down	0.0016	0.1157
BB794657	Igf2bp1	Down	0.0001	0.0694
NM_023670	Igf2bp3	Down	0.0149	0.2045
BG092043	Igf2bp3	Down	0.0323	0.2564
X88902	Igh	Down	0.0242	0.2388
NM_008355	Il13	Down	0.0247	0.2400
AJ277831	Il1rapl2	Down	0.0159	0.2093
AV301185	Incenp	Down	0.0467	0.2752
BQ176731	Inpp4b	Down	0.0035	0.1349
BI083036	Iqgap3	Down	0.0310	0.2546
NM_029926	Irak4	Down	0.0211	0.2271
BM245932	Itk	Down	0.0291	0.2513
C78652	Jmjd6	Down	0.0064	0.1570
BF322939	Kcnk12	Down	0.0118	0.1937
AV133559	Kcnt2	Down	0.0267	0.2448
BB239851	Kdm6a	Down	0.0193	0.2207
BG082186	Khdc1b	Down	0.0003	0.0844
NM_024245	Kif23	Down	0.0139	0.2030
NM_019974	Klk11	Down	0.0043	0.1404
BC013660	Klk1b11	Down	0.0140	0.2030
AU020154	Krr1	Down	0.0246	0.2400
NM_016880	Krt35	Down	0.0275	0.2469
NM_008476	Krt6a	Down	0.0303	0.2541
BB144669	Krt78	Down	0.0353	0.2602
AK014635	Krtap31-1	Down	0.0130	0.1988
AK012581	Limd2	Down	0.0472	0.2752
BB518473	Lrat	Down	0.0180	0.2166
BB494139	Lrba	Down	0.0346	0.2602
AV231827	Lrch2	Down	0.0076	0.1648
BB093349	Lrfn4	Down	0.0301	0.2537
AK006656	Lrrc50	Down	0.0396	0.2666
AK014850	Lrrc63	Down	0.0145	0.2038
NM_008522	Ltf	Down	0.0132	0.1993
AI326034	Lyzl6	Down	0.0440	0.2731
AV284857	Maf	Down	0.0256	0.2425
BQ176021	Maneal	Down	0.0384	0.2645
BE947490	Mapk10	Down	0.0376	0.2645
AF128892	Mapk14	Down	0.0322	0.2564
BB184171	Mapk8	Down	0.0451	0.2741
NM_008552	Mas1	Down	0.0261	0.2440
BB328498	Mast4	Down	0.0162	0.2111
AK010648	Mcm10	Down	0.0327	0.2564
BB310008	Mecr	Down	0.0279	0.2498
NM_053172	Mepe	Down	0.0306	0.2541
NM_010795	Mgat3	Down	0.0092	0.1767
BG063791	Mib1	Down	0.0044	0.1430
BG095454	Mocs3	Down	0.0226	0.2354
BM115860	Morf4l1	Down	0.0440	0.2731
NM_010824	Mpo	Down	0.0004	0.0844
BB390252	Mtmr3	Down	0.0399	0.2678
NM_010850	Mycs	Down	0.0011	0.0926
BB011213	Myh8	Down	0.0141	0.2030
BB251623	Nanos1	Down	0.0149	0.2045
AI451602	Nav1	Down	0.0478	0.2768
AU067780	Nav2	Down	0.0494	0.2801
BF019797	Nbeal2	Down	0.0141	0.2030
AK010307	Ndufab1	Down	0.0142	0.2033
M20480	Nefl	Down	0.0213	0.2271
NM_021360	Neurl1a	Down	0.0400	0.2678
AW049749	Neurl1a	Down	0.0034	0.1330
AF369980	Nfat5	Down	0.0218	0.2310
AF289078	Nfatc2	Down	0.0460	0.2749
BB485864	Nfia	Down	0.0036	0.1349
NM_008694	Ngp	Down	0.0207	0.2265
BB204913	Nlrc3	Down	0.0236	0.2380
AK005959	Nol4	Down	0.0031	0.1330
NM_010933	Nppc	Down	0.0293	0.2516
NM_016708	Npy5r	Down	0.0073	0.1636
NM_008734	Nrg3	Down	0.0387	0.2645
NM_018766	Ntsr1	Down	0.0414	0.2688
AI853080	Nudt10	Down	0.0281	0.2501
AV270366	Nup210l	Down	0.0286	0.2506
AB067535	Oas2	Down	0.0434	0.2729
AA144866	Oaz3	Down	0.0464	0.2751
BM118129	Odz3	Down	0.0470	0.2752
AI461774	OTTMUSG00000015160	Down	0.0040	0.1383
BB370469	Pcbp2	Down	0.0038	0.1358
C76364	Pdcd6ip	Down	0.0009	0.0898
BE981897	Pfkfb2	Down	0.0379	0.2645
AK014380	Phf19	Down	0.0285	0.2506
BB490622	Plekha5	Down	0.0149	0.2045
BB280013	Plekhg3	Down	0.0452	0.2741
AI326372	Pnlip	Down	0.0060	0.1553
NM_011128	Pnliprp2	Down	0.0467	0.2752
BB501129	Pnpt1	Down	0.0444	0.2731
NM_030715	Polh	Down	0.0138	0.2030
AK005318	Pot1a	Down	0.0471	0.2752
AK004360	Ppp3cb	Down	0.0002	0.0784
BB283625	Prdm14	Down	0.0284	0.2501
AB062122	Prkcq	Down	0.0344	0.2599
X75557	Prl2c2	Down	0.0055	0.1553
AI194999	Apoa1	Up	0.0478	0.3617
AI527359	Apoa1	Up	0.0058	0.3178
BC021776	Apoc3	Up	0.0457	0.3617
BB193413	Aqp4	Up	0.0445	0.3617
AF297029	Arhgap10	Up	0.0019	0.2387
AK014880	Armc3	Up	0.0025	0.2465
BG069160	Atp10b	Up	0.0083	0.3617
BC027319	Atp1b1	Up	0.0371	0.3617
BC027319	Atp1b1	Up	0.0425	0.3617
BB456872	Atp8a2	Up	0.0140	0.3617
AV274423	B230396O12Rik	Up	0.0019	0.2387
BB178985	B3gnt6	Up	0.0116	0.3617
BE979636	Bai3	Up	0.0143	0.3617
NM_019446	Barhl1	Up	0.0408	0.3617
BB001852	BC033915	Up	0.0384	0.3617
BG066622	Brwd3	Up	0.0179	0.3617
BB467799	Bzrap1	Up	0.0171	0.3617
NM_021415	Cacna1h	Up	0.0402	0.3617
AF247140	Cacna2d2	Up	0.0219	0.3617
BM238658	Cand2	Up	0.0027	0.2465
BG093811	Ccdc102a	Up	0.0192	0.3617
AV275573	Ccdc108	Up	0.0421	0.3617
AV279772	Ccdc113	Up	0.0408	0.3617
AI845986	Ccdc28b	Up	0.0481	0.3617
AV280756	Ccdc40	Up	0.0305	0.3617
AW208904	Ccdc88b	Up	0.0328	0.3617
BB479310	Cdc14a	Up	0.0108	0.3617
AI425726	Cdh4	Up	0.0303	0.3617
NM_021050	Cftr	Up	0.0063	0.3239
BB184676	Chfr	Up	0.0004	0.1927
BC011490	Chrna2	Up	0.0363	0.3617
AF486651	Cldn19	Up	0.0307	0.3617
NM_016675	Cldn2	Up	0.0401	0.3617
NM_025469	Clps	Up	0.0192	0.3617
BC024605	Cml5	Up	0.0192	0.3617
AW553181	Cntn4	Up	0.0451	0.3617
BQ173923	Cobl	Up	0.0025	0.2465
AU019881	Cphx	Up	0.0356	0.3617
BC027090	Crocc	Up	0.0430	0.3617
AU045294	Crxos1	Up	0.0209	0.3617
BB698378	D15Wsu169e	Up	0.0325	0.3617
NM_028146	Dbndd1	Up	0.0274	0.3617
BB179083	Disp2	Up	0.0430	0.3617
AW492102	Dnahc9	Up	0.0320	0.3617
AV276323	E130009J12Rik	Up	0.0166	0.3617
BE947444	E230016M11Rik	Up	0.0272	0.3617
AV380908	EG627821	Up	0.0276	0.3617
BC026387	Eppk1	Up	0.0213	0.3617
NM_133191	Eps8l2	Up	0.0180	0.3617
NM_007950	Ereg	Up	0.0472	0.3617
AB073079	Erg	Up	0.0480	0.3617
NM_007954	Es1	Up	0.0400	0.3617
NM_007980	Fabp2	Up	0.0154	0.3617
BB474395	Fam179a	Up	0.0058	0.3178
AI595406	Fbll1	Up	0.0342	0.3617
NM_008011	Fgfr4	Up	0.0260	0.3617
BB234926	Foxn1	Up	0.0226	0.3617
NM_013521	Fpr1	Up	0.0431	0.3617
BB468955	Garnl1	Up	0.0336	0.3617
NM_008127	Gjb4	Up	0.0465	0.3617
AU041329	Gnb5	Up	0.0058	0.3178
BE947846	Gpr125	Up	0.0283	0.3617
BB028100	Gprasp2	Up	0.0447	0.3617
NM_008165	Gria1	Up	0.0439	0.3617
NM_133256	Gsx2	Up	0.0181	0.3617
BB821611	Gtf3c1	Up	0.0218	0.3617
BB347007	Hecw1	Up	0.0291	0.3617
AB008174	Hnf1b	Up	0.0006	0.1927
NM_010483	Htr5b	Up	0.0398	0.3617
AF065324	Igh-6	Up	0.0361	0.3617
M28052	Il2rb	Up	0.0295	0.3617
NM_018885	Irx4	Up	0.0465	0.3617
NM_008423	Kcnd1	Up	0.0148	0.3617
AU067277	Kif5a	Up	0.0014	0.2387
NM_010915	Klk1b4	Up	0.0237	0.3617
AK009086	Krtap26-1	Up	0.0383	0.3617
AK013209	Lhx9	Up	0.0194	0.3617
NM_010727	Lnx1	Up	0.0255	0.3617
NM_020028	Lpar2	Up	0.0424	0.3617
AK015164	Lrrc67	Up	0.0113	0.3617
AW541674	Map2k7	Up	0.0328	0.3617
M18775	Mapt	Up	0.0348	0.3617
NM_008629	Msi1	Up	0.0481	0.3617
AW743000	Mybpc1	Up	0.0245	0.3617
BC003985	Myo5c	Up	0.0157	0.3617
NM_032394	Myo7b	Up	0.0187	0.3617
BB049667	Nek5	Up	0.0256	0.3617
AK018265	Nhsl1	Up	0.0162	0.3617
AI527293	Nlrp12	Up	0.0288	0.3617
BM225203	Nlrp4e	Up	0.0404	0.3617
BB071996	Nlrp6	Up	0.0392	0.3617
AV323033	Nol4	Up	0.0229	0.3617
BE980583	Nr4a3	Up	0.0306	0.3617
AK020731	Nrip3	Up	0.0097	0.3617
BE647993	Nrn1l	Up	0.0011	0.2293
AB067530	Oas1h	Up	0.0020	0.2387
BB132229	Olfr78	Up	0.0372	0.3617
BB101283	Pappa2	Up	0.0094	0.3617
BB745015	Pcdh24	Up	0.0482	0.3617
NM_008795	Pctk3	Up	0.0140	0.3617
AV174487	Pdzrn4	Up	0.0089	0.3617

NM_013639	Prph	Down	0.0169	0.2120
AK014289	Prr8	Down	0.0026	0.1330
AW537978	Prtg	Down	0.0126	0.1951
AF113598	Psg28	Down	0.0085	0.1707
NM_011195	Ptcra	Down	0.0273	0.2469
AU044268	Ptdss1	Down	0.0190	0.2199
AU018448	Pttg1ip	Down	0.0069	0.1617
BM239661	Pum2	Down	0.0311	0.2546
BB474318	Pvr	Down	0.0471	0.2752
AW060288	Qk	Down	0.0100	0.1855
NM_134050	Rab15	Down	0.0126	0.1951
BB817863	Rabgap1	Down	0.0368	0.2637
AA990557	Raf1	Down	0.0022	0.1312
NM_009019	Rag1	Down	0.0010	0.0919
U27178	Rbl1	Down	0.0058	0.1553
NM_133242	Rbm39	Down	0.0025	0.1322
BB449095	Rbm5	Down	0.0212	0.2271
NM_011252	Rbmx	Down	0.0231	0.2370
NM_030598	Rcan2	Down	0.0412	0.2686
BE986062	Repin1	Down	0.0283	0.2501
BB041370	Rhobtb1	Down	0.0148	0.2045
AV269723	Rhox13	Down	0.0021	0.1311
NM_021300	Rhox4b	Down	0.0041	0.1392
NM_008955	Rhox6	Down	0.0109	0.1901
AK018316	Rif1	Down	0.0369	0.2637
BG065807	Rif1	Down	0.0461	0.2749
BE956483	Rlbp1l1	Down	0.0022	0.1311
BB144036	Rnf185	Down	0.0160	0.2098
AF155141	Rp1	Down	0.0214	0.2272
AF155141	Rp1	Down	0.0303	0.2541
AW536522	Rpap1	Down	0.0131	0.1992
X57960	Rpl7	Down	0.0414	0.2688
BG143502	Rreb1	Down	0.0136	0.2015
BB053418	Rsf1	Down	0.0057	0.1553
BB700837	Rttn	Down	0.0037	0.1349
AW489945	Rundc3a	Down	0.0351	0.2602
AK012504	Rwdd1	Down	0.0479	0.2769
NM_053190	S1pr5	Down	0.0469	0.2752
AK015593	Samd8	Down	0.0312	0.2546
BB706191	Saps3	Down	0.0373	0.2643
NM_018732	Scn3a	Down	0.0009	0.0898
AW540070	Seh1l	Down	0.0022	0.1311
NM_011345	Sele	Down	0.0006	0.0870
AI462524	Serpinb5	Down	0.0112	0.1914
AV225626	Serpinb9d	Down	0.0140	0.2030
AI466898	Setd2	Down	0.0001	0.0568
BB410394	Sfmbt2	Down	0.0364	0.2630
BB410537	Sgol1	Down	0.0367	0.2637
BI714008	Shc2	Down	0.0029	0.1330
AI893638	Six4	Down	0.0251	0.2424
AI647939	Slc16a5	Down	0.0289	0.2506
NM_009200	Slc1a6	Down	0.0219	0.2315
BB448377	Slc4a1	Down	0.0007	0.0880
NM_011403	Slc4a1	Down	0.0004	0.0844
BB003029	Slc5a9	Down	0.0302	0.2541
NM_020519	Slurp1	Down	0.0123	0.1951
BB135507	Smarca5	Down	0.0115	0.1926
M60510	Smarcd2	Down	0.0002	0.0844
BB238756	Snai3	Down	0.0484	0.2781
BF318702	Spag17	Down	0.0033	0.1330
BG066112	Spata5	Down	0.0445	0.2731
NM_007463	Speg	Down	0.0184	0.2188
BC027796	Spin4	Down	0.0005	0.0844
AI593292	Spred2	Down	0.0289	0.2506
BB440691	Srcap	Down	0.0013	0.1043
AV369536	Steap2	Down	0.0419	0.2695
AW146083	Stfa1	Down	0.0005	0.0844
BB667930	Stfa2l1	Down	0.0053	0.1533
AW536442	Suz12	Down	0.0162	0.2111
BB087539	Svopl	Down	0.0206	0.2265
NM_009317	Tal2	Down	0.0343	0.2595
BB352105	Tbc1d16	Down	0.0409	0.2684
NM_009323	Tbx15	Down	0.0058	0.1553
NM_009331	Tcf7	Down	0.0021	0.1311
AK017244	Tchhl1	Down	0.0446	0.2731
BF318536	Tcrb-V13	Down	0.0498	0.2804
M16120	Tcrb-V13	Down	0.0339	0.2595
M11456	Tcrb-V13	Down	0.0452	0.2741
U46841	Tcrb-V13	Down	0.0164	0.2114
U63547	Tcrb-V13	Down	0.0072	0.1634
BB217651	Terf2ip	Down	0.0232	0.2370
BI134710	Tigd5	Down	0.0313	0.2546
BG066908	Timm8a2	Down	0.0188	0.2199
NM_011904	Tll2	Down	0.0015	0.1125
AW545765	Tmed7	Down	0.0023	0.1322
AW551464	Tmem19	Down	0.0122	0.1951
BF138523	Tmprss13	Down	0.0363	0.2628
AW045551	Tnik	Down	0.0034	0.1330
AF307746	Tnk1	Down	0.0145	0.2038
AV007148	Tnni2	Down	0.0081	0.1668
AA119213	Tnpo1	Down	0.0038	0.1373
AY034576	Tpbpb	Down	0.0030	0.1330
BB224683	Traf6	Down	0.0485	0.2781
BG069740	Trim61	Down	0.0096	0.1803
BQ174474	Trim9	Down	0.0028	0.1330
AK018525	Trub1	Down	0.0196	0.2224
BE628523	Tsepa	Down	0.0472	0.2753
BG806300	Tsix	Down	0.0048	0.1482
AF138745	Tsix	Down	0.0475	0.2757
BB711506	Ttc14	Down	0.0372	0.2643
BF715463	Tube1	Down	0.0427	0.2721
AV100095	Tube1	Down	0.0058	0.1553

NM_008827	Pgf	Up	0.0451	0.3617
AK020902	Pigh	Up	0.0079	0.3617
NM_008840	Pik3cd	Up	0.0188	0.3617
AV275795	Plb1	Up	0.0017	0.2387
AV257260	Plcd4	Up	0.0383	0.3617
BB274799	Plcl1	Up	0.0210	0.3617
BC026410	Pls1	Up	0.0450	0.3617
BM119522	Plxnb1	Up	0.0439	0.3617
M22958	Prlr	Up	0.0018	0.2387
AV329802	Prrt3	Up	0.0128	0.3617
D28531	Ptprs	Up	0.0013	0.2346
AV216875	Ran	Up	0.0128	0.3617
BC008101	Rasl11b	Up	0.0017	0.2387
NM_009040	Rdh16	Up	0.0198	0.3617
NM_017473	Rdh7	Up	0.0431	0.3617
BB427703	RP23-100C5.8	Up	0.0164	0.3617
BB335578	RP23-12I24.6	Up	0.0271	0.3617
NM_029987	Rpe65	Up	0.0011	0.2293
BB449218	Rps6ka6	Up	0.0089	0.3617
AI413817	Rsph10b2	Up	0.0155	0.3617
BB739342	Sall1	Up	0.0165	0.3617
BI133839	Scube2	Up	0.0282	0.3617
AK003320	Shroom3	Up	0.0299	0.3617
AK005699	Slc16a13	Up	0.0305	0.3617
NM_009202	Slc22a1	Up	0.0422	0.3617
AI847460	Slc24a2	Up	0.0159	0.3617
NM_019741	Slc2a5	Up	0.0278	0.3617
BF661644	Smc2	Up	0.0370	0.3617
C80870	Smg1	Up	0.0430	0.3617
BF160651	Smyd5	Up	0.0388	0.3617
BM214097	Snap91	Up	0.0165	0.3617
BC024958	Sox9	Up	0.0359	0.3617
X81633	Spag5	Up	0.0458	0.3617
NM_027609	Speer4f	Up	0.0003	0.1362
AV360142	Sptlc3	Up	0.0254	0.3617
BB189586	Stoml3	Up	0.0449	0.3617
NM_011902	Tekt2	Up	0.0383	0.3617
BG075369	Tmem174	Up	0.0006	0.1927
BB528158	Tmem44	Up	0.0317	0.3617
NM_015775	Tmprss2	Up	0.0337	0.3617
NM_080727	Tmprss3	Up	0.0322	0.3617
AK016101	Tomm20l	Up	0.0212	0.3617
U42405	Tpsab1	Up	0.0247	0.3617
AV272776	Ttc21a	Up	0.0408	0.3617
BC027099	Ubxn10	Up	0.0393	0.3617
BC027099	Ubxn10	Up	0.0475	0.3617
NM_023649	Ush1c	Up	0.0245	0.3617
NM_021408	Ush2a	Up	0.0123	0.3617
NM_009509	Vil1	Up	0.0386	0.3617
AW553039	Vps29	Up	0.0239	0.3617
BB212004	Vwce	Up	0.0183	0.3617
BC027038	Wasf3	Up	0.0323	0.3617
AW909375	Wbp11	Up	0.0104	0.3617
AK006118	Wdr16	Up	0.0460	0.3617
AF334269	Wfdc2	Up	0.0479	0.3617
BC026460	Wfikkn1	Up	0.0433	0.3617
BB045268	Zbtb12	Up	0.0404	0.3617
AW413620	Zfp704	Up	0.0362	0.3617

BB762957	Tyrp1	Down	0.0272	0.2469
U46068	U46068	Down	0.0089	0.1748
BB397001	Ubash3a	Down	0.0059	0.1553
BI653033	Ubc	Down	0.0027	0.1330
BE950186	Ubqln2	Down	0.0396	0.2666
AW494942	Ubxn10	Down	0.0027	0.1330
AK003750	Ucma	Down	0.0171	0.2128
BG069400	Unc13c	Down	0.0196	0.2224
AW107924	Usp14	Down	0.0028	0.1330
BB771589	Usp19	Down	0.0433	0.2729
NM_053225	V1rb1	Down	0.0050	0.1504
AW123067	Wapal	Down	0.0047	0.1482
AU017197	Wwc1	Down	0.0226	0.2354
L04961	Xist	Down	0.0368	0.2637
BQ176814	Xkr4	Down	0.0465	0.2751
AW558771	Xrcc6	Down	0.0048	0.1482
NM_009535	Yes1	Down	0.0497	0.2804
BB201861	Yod1	Down	0.0065	0.1589
BI665246	Yy1	Down	0.0033	0.1330
BE986026	Zbtb7a	Down	0.0372	0.2643
BG076273	Zc3h4	Down	0.0250	0.2418
AV041291	Zdhhc25	Down	0.0114	0.1918
NM_021307	Zfp112	Down	0.0065	0.1589
AF281141	Zfp287	Down	0.0126	0.1951
NM_013889	Zfp292	Down	0.0003	0.0844
BG073454	Zfp462	Down	0.0033	0.1330
BF729651	2-Mar	Up	0.0260	0.2437
AV269187	-	Up	0.0124	0.1951
BB255007	-	Up	0.0010	0.0898
BB278809	-	Up	0.0028	0.1330
BE630431	-	Up	0.0027	0.1330
AV210911	-	Up	0.0003	0.0844
BG069060	-	Up	0.0104	0.1881
BB013007	-	Up	0.0389	0.2645
M35669	-	Up	0.0438	0.2731
BE943696	-	Up	0.0229	0.2366
BQ176223	-	Up	0.0090	0.1755
BB250300	-	Up	0.0360	0.2622
AK017941	-	Up	0.0177	0.2166
BB041802	-	Up	0.0052	0.1517
BB137915	-	Up	0.0302	0.2541
AW121529	-	Up	0.0034	0.1330
AK007161	-	Up	0.0033	0.1330
AI662851	-	Up	0.0382	0.2645
AV244995	-	Up	0.0374	0.2643
BE994109	-	Up	0.0100	0.1865
AK006226	-	Up	0.0203	0.2265
BG067318	-	Up	0.0058	0.1553
BB219601	-	Up	0.0164	0.2114
BM228792	-	Up	0.0191	0.2199
BG067908	-	Up	0.0497	0.2804
BB076574	-	Up	0.0105	0.1881
NM_026351	-	Up	0.0005	0.0844
AK020729	-	Up	0.0435	0.2729
BM123577	-	Up	0.0170	0.2128
BB180782	-	Up	0.0166	0.2119
AK010967	-	Up	0.0031	0.1330
AK017366	-	Up	0.0054	0.1540
BF159226	-	Up	0.0082	0.1669
AA517858	-	Up	0.0092	0.1767
BC023100	-	Up	0.0009	0.0898
AI481875	-	Up	0.0414	0.2688
AK011086	-	Up	0.0170	0.2123
BB375376	-	Up	0.0262	0.2440
AK015891	-	Up	0.0073	0.1636
BB125729	-	Up	0.0067	0.1608
AK012683	-	Up	0.0010	0.0919
BB275292	-	Up	0.0034	0.1330
BB253608	-	Up	0.0338	0.2594
AK021053	-	Up	0.0436	0.2729
AK013567	-	Up	0.0144	0.2037
BG065774	-	Up	0.0009	0.0898
AK021412	-	Up	0.0500	0.2809
BB110590	-	Up	0.0179	0.2166
BB430390	-	Up	0.0160	0.2099
AK015097	-	Up	0.0220	0.2315
AW047760	-	Up	0.0234	0.2370
AI649303	-	Up	0.0177	0.2166
BE986579	-	Up	0.0018	0.1226
BB614236	-	Up	0.0060	0.1553
AU022616	-	Up	0.0453	0.2741
AW124794	-	Up	0.0014	0.1093
BB718075	-	Up	0.0062	0.1570
BB663137	-	Up	0.0168	0.2120
AK010815	-	Up	0.0153	0.2061
AK016834	-	Up	0.0419	0.2695
BB627097	-	Up	0.0032	0.1330
AK019632	-	Up	0.0338	0.2594
NM_008785	-	Up	0.0023	0.1322
AK016639	-	Up	0.0460	0.2749
AI593498	-	Up	0.0282	0.2501
BG066467	-	Up	0.0190	0.2199
BB155266	-	Up	0.0210	0.2271
BE957407	-	Up	0.0240	0.2388
NM_026621	-	Up	0.0035	0.1349
AK020674	-	Up	0.0049	0.1489
AK016607	-	Up	0.0234	0.2370
BB205090	-	Up	0.0006	0.0870
BG064549	-	Up	0.0359	0.2622
BB536585	-	Up	0.0495	0.2801
BB375206	-	Up	0.0387	0.2645
AK006986	-	Up	0.0112	0.1914

BG074159	-	Up	0.0328	0.2564
AK020112	-	Up	0.0036	0.1349
AK017010	-	Up	0.0374	0.2643
C79309	-	Up	0.0464	0.2751
BG070618	-	Up	0.0254	0.2425
BB183109	-	Up	0.0453	0.2741
AK015377	-	Up	0.0318	0.2564
BB445605	-	Up	0.0139	0.2030
NM_008271	-	Up	0.0049	0.1489
BG866612	-	Up	0.0125	0.1951
AV230414	-	Up	0.0138	0.2030
BG068545	-	Up	0.0341	0.2595
BB269821	-	Up	0.0350	0.2602
BB040120	-	Up	0.0369	0.2637
BG072095	-	Up	0.0082	0.1668
BB740266	-	Up	0.0347	0.2602
BM730983	-	Up	0.0375	0.2645
BI656407	-	Up	0.0092	0.1767
C80868	-	Up	0.0422	0.2699
AK012411	-	Up	0.0342	0.2595
NM_009310	-	Up	0.0135	0.2014
AB041554	-	Up	0.0384	0.2645
BB026304	-	Up	0.0081	0.1668
AK007209	-	Up	0.0188	0.2199
NM_029241	-	Up	0.0381	0.2645
BE685969	-	Up	0.0468	0.2752
BB763153	-	Up	0.0153	0.2061
AK005945	-	Up	0.0297	0.2524
AK014925	-	Up	0.0294	0.2516
AK009733	-	Up	0.0431	0.2729
BB463139	-	Up	0.0040	0.1383
AK012003	-	Up	0.0388	0.2645
BB045793	-	Up	0.0498	0.2804
AK018489	-	Up	0.0469	0.2752
BM244157	-	Up	0.0398	0.2674
AK009690	-	Up	0.0310	0.2546
BB648848	-	Up	0.0482	0.2780
AK006072	-	Up	0.0197	0.2224
AK014888	-	Up	0.0415	0.2688
AK019986	-	Up	0.0448	0.2736
AK014433	-	Up	0.0148	0.2045
BE952823	-	Up	0.0375	0.2645
AV153710	-	Up	0.0307	0.2544
AV210811	-	Up	0.0124	0.1951
AK017959	-	Up	0.0001	0.0568
BG069076	-	Up	0.0090	0.1752
BG070574	-	Up	0.0030	0.1330
AK013712	-	Up	0.0428	0.2725
AI847438	-	Up	0.0275	0.2469
BB324785	-	Up	0.0168	0.2120
AK013026	-	Up	0.0181	0.2166
AK009736	-	Up	0.0223	0.2334
BF682509	-	Up	0.0134	0.2011
AB054000	1110028A07Rik	Up	0.0026	0.1330
AK005740	1700007N14Rik	Up	0.0387	0.2645
AK006434	1700027L20Rik	Up	0.0257	0.2425
AK007013	1700086D15Rik	Up	0.0243	0.2388
AK009747	2310042D19Rik	Up	0.0211	0.2271
BB349413	2410129H14Rik	Up	0.0274	0.2469
BC024774	2610034M16Rik	Up	0.0207	0.2265
BI134263	2900083I11Rik	Up	0.0251	0.2424
AK014652	4833403I15Rik	Up	0.0284	0.2501
BC020160	4833422F24Rik	Up	0.0169	0.2120
NM_025737	4931417E11Rik	Up	0.0063	0.1570
AK016775	4933411K16Rik	Up	0.0436	0.2729
NM_025746	4933415F23Rik	Up	0.0268	0.2448
AK020314	9230104L09Rik	Up	0.0327	0.2564
M85170	Acr	Up	0.0083	0.1684
NM_007394	Acvr1	Up	0.0165	0.2114
NM_026531	Aen	Up	0.0009	0.0898
BB698513	Afap1	Up	0.0200	0.2255
AJ429133	Alg12	Up	0.0197	0.2225
L34570	Alox15	Up	0.0202	0.2265
AK003821	Anapc5	Up	0.0243	0.2388
BC012275	Ankmy2	Up	0.0372	0.2643
NM_007477	Arf2	Up	0.0004	0.0844
AF297029	Arhgap10	Up	0.0005	0.0844
BG295243	Arhgap22	Up	0.0104	0.1881
BE651011	Arhgef7	Up	0.0454	0.2741
BQ042885	Atcay	Up	0.0467	0.2752
BB393498	Atic	Up	0.0207	0.2265
NM_016745	Atp2a3	Up	0.0309	0.2546
AF200346	Bace1	Up	0.0208	0.2265
BB001852	BC033915	Up	0.0068	0.1608
BF302511	BC048546	Up	0.0102	0.1881
BC004826	Bcam	Up	0.0444	0.2731
NM_009751	Bfsp1	Up	0.0040	0.1383
NM_007563	Bpgm	Up	0.0031	0.1330
BI466363	Brca1	Up	0.0023	0.1322
BE984258	Bre	Up	0.0180	0.2166
AV144956	Bzw1	Up	0.0325	0.2564
BB278653	Cacna2d4	Up	0.0314	0.2551
BE985916	Camsap1	Up	0.0491	0.2794
NM_007617	Cav3	Up	0.0361	0.2627
AK010251	Ccdc77	Up	0.0116	0.1929
AF065933	Ccl2	Up	0.0304	0.2541
AJ416459	Ccnb3	Up	0.0323	0.2564
AL024092	Cct3	Up	0.0024	0.1322
AK005507	Cd59a	Up	0.0069	0.1617
NM_009690	Cd5l	Up	0.0416	0.2689
NM_008339	Cd79b	Up	0.0153	0.2061
AK004547	Cdc5l	Up	0.0101	0.1873

BC006628	Cenpb	Up	0.0412	0.2686
NM_031258	Chrdl1	Up	0.0499	0.2809
AF486651	Cldn19	Up	0.0080	0.1668
NM_011606	Clec3b	Up	0.0257	0.2425
U53455	Clns1a	Up	0.0154	0.2069
NM_007753	Cpa3	Up	0.0158	0.2093
BM933756	Crh	Up	0.0138	0.2030
NM_024223	Crip2	Up	0.0190	0.2199
AU045294	Crxos1	Up	0.0203	0.2265
AK010433	Cryzl1	Up	0.0020	0.1288
AK020589	Cstad	Up	0.0017	0.1186
NM_030249	Cttnbp2nl	Up	0.0423	0.2702
AA023906	Cux1	Up	0.0040	0.1383
AF010586	Cx3cl1	Up	0.0446	0.2731
NM_028375	Cxx1c	Up	0.0391	0.2645
AF332060	Cyb5r3	Up	0.0062	0.1570
NM_007807	Cybb	Up	0.0330	0.2574
BQ175587	D3Bwg0562e	Up	0.0283	0.2501
BE447520	D4Wsu53e	Up	0.0460	0.2749
NM_026302	Dctn4	Up	0.0238	0.2384
AI841578	Ddn	Up	0.0267	0.2448
NM_007845	Defa-rs10	Up	0.0404	0.2680
NM_015789	Dkkl1	Up	0.0016	0.1133
NM_015802	Dlc1	Up	0.0325	0.2564
AK004739	Dll4	Up	0.0008	0.0898
AI664344	Dnajb5	Up	0.0062	0.1570
BB224547	Dnajc21	Up	0.0334	0.2589
BG066325	Dnm2	Up	0.0114	0.1921
NM_007878	Drd4	Up	0.0111	0.1914
NM_025975	Dynlt3	Up	0.0315	0.2557
BC008277	Ednra	Up	0.0026	0.1330
BC025062	Efcab2	Up	0.0105	0.1881
D38146	Efna1	Up	0.0274	0.2469
AI507006	EG627607	Up	0.0010	0.0898
AW701127	Eif3a	Up	0.0123	0.1951
BC005686	Elk3	Up	0.0037	0.1349
AB034693	Emcn	Up	0.0031	0.1330
AF339910	Enpp1	Up	0.0104	0.1881
BQ126025	ENSMUSG0000 0061115	Up	0.0378	0.2645
BE951907	Epb4.1l2	Up	0.0225	0.2349
BB492312	Epb4.9	Up	0.0337	0.2594
AF353717	Epdr1	Up	0.0133	0.1993
NM_007938	Epha6	Up	0.0262	0.2440
AB073079	Erg	Up	0.0177	0.2166
AV329219	Erg	Up	0.0076	0.1648
AK007800	Erp27	Up	0.0125	0.1951
BB612598	Esf1	Up	0.0066	0.1592
BE652876	Fads3	Up	0.0040	0.1383
BB400295	Fam154a	Up	0.0033	0.1330
BB629037	Fam49a	Up	0.0411	0.2686
BB205408	Farsa	Up	0.0078	0.1656
BB555130	Fbxw13	Up	0.0445	0.2731
AF176528	Fbxw14	Up	0.0247	0.2400
M14216	Fcgr2b	Up	0.0024	0.1322
BM224327	Fcgr2b	Up	0.0014	0.1125
BI247584	Fdps	Up	0.0079	0.1661
NM_008006	Fgf2	Up	0.0291	0.2513
BB283818	Fndc7	Up	0.0135	0.2011
U41047	Foxd3	Up	0.0205	0.2265
NM_007984	Fscn1	Up	0.0061	0.1567
BG067296	Fut10	Up	0.0203	0.2265
NM_010243	Fut9	Up	0.0009	0.0898
NM_008067	Gabra3	Up	0.0495	0.2801
AV348821	Gabrg2	Up	0.0232	0.2370
AF348968	Galnt2	Up	0.0185	0.2188
BC021462	Gcc1	Up	0.0482	0.2780
AK013104	Gdi2	Up	0.0179	0.2166
BB750040	Gfap	Up	0.0437	0.2729
AV148957	Gjc1	Up	0.0005	0.0844
AF276917	Glrx	Up	0.0312	0.2546
AV122321	Glt25d1	Up	0.0087	0.1734
AU041329	Gnb5	Up	0.0055	0.1553
BI103049	Gpr146	Up	0.0116	0.1928
NM_022320	Gpr35	Up	0.0484	0.2781
BM899529	Grik3	Up	0.0212	0.2271
NM_010358	Gstm1	Up	0.0059	0.1553
AV325174	Gtf2f1	Up	0.0034	0.1330
BB821611	Gtf3c1	Up	0.0255	0.2425
BC019856	Gulo	Up	0.0002	0.0784
M33151	H2-K1	Up	0.0392	0.2648
AK006835	Hbp1	Up	0.0237	0.2380
BB490701	Hist1h1e	Up	0.0005	0.0870
NM_010438	Hk1	Up	0.0192	0.2207
BB225704	Hmgcl	Up	0.0134	0.2011
BB822465	Hnrnpr	Up	0.0269	0.2457
NM_026489	Hormad1	Up	0.0337	0.2594
NM_019424	Hps1	Up	0.0297	0.2524
BG072212	Hs3st3b1	Up	0.0445	0.2731
AK021006	Hspa13	Up	0.0415	0.2688
BG968304	Hspb7	Up	0.0031	0.1330
BE957307	Iffo1	Up	0.0402	0.2679
U29768	Igk-V1	Up	0.0295	0.2516
NM_008348	Il10ra	Up	0.0405	0.2680
NM_133193	Il1rl2	Up	0.0344	0.2601
AF000304	Il4ra	Up	0.0214	0.2272
AA717838	Il6st	Up	0.0211	0.2271
BB222675	Immt	Up	0.0075	0.1643
BB661182	Ints8	Up	0.0007	0.0880
BB344549	Islr2	Up	0.0292	0.2516
BB623587	Itga8	Up	0.0156	0.2089
NM_008401	Itgam	Up	0.0300	0.2537

AF319542	Kcnk5	Up	0.0063	0.1570
AU079968	Klc1	Up	0.0470	0.2752
BE995545	Klf9	Up	0.0078	0.1656
AW544889	Kpnb1	Up	0.0327	0.2564
AK009746	Kptn	Up	0.0327	0.2564
BB530994	Layn	Up	0.0075	0.1643
BC019124	Lmcd1	Up	0.0237	0.2380
L41881	LOC213481	Up	0.0225	0.2349
NM_022883	Lpin3	Up	0.0007	0.0870
AK005871	Lrrc67	Up	0.0002	0.0784
BM224801	Lrrtm3	Up	0.0054	0.1540
AF232024	Ly6i	Up	0.0125	0.1951
NM_010749	M6pr	Up	0.0383	0.2645
BC012256	Mall	Up	0.0011	0.0926
AF128892	Mapk14	Up	0.0168	0.2120
NM_010766	Marco	Up	0.0008	0.0898
AF072245	Mbd2	Up	0.0081	0.1668
BB003847	Mbnl2	Up	0.0095	0.1801
BC026198	Mcpt4	Up	0.0110	0.1910
AA764430	Mea1	Up	0.0109	0.1901
NM_008591	Met	Up	0.0127	0.1951
NM_027334	Mettl7a1	Up	0.0415	0.2688
BI661422	Mfap3	Up	0.0027	0.1330
AV280494	Mfap3l	Up	0.0334	0.2589
NM_015776	Mfap5	Up	0.0464	0.2751
AW494220	Mgl2	Up	0.0334	0.2589
BQ042988	Mmab	Up	0.0252	0.2425
NM_020021	Mos	Up	0.0410	0.2684
NM_138670	Mpst	Up	0.0240	0.2388
BB041267	Mrpl19	Up	0.0039	0.1383
NM_024174	Mrps23	Up	0.0138	0.2030
BB236617	Ms4a1	Up	0.0403	0.2679
BB462562	Msi1	Up	0.0272	0.2469
NM_026002	Mtdh	Up	0.0383	0.2645
AJ002522	Myh1	Up	0.0403	0.2680
NM_008659	Myo1c	Up	0.0332	0.2583
NM_031189	Myog	Up	0.0003	0.0844
BM212838	Mypn	Up	0.0142	0.2033
BF118393	Ncl	Up	0.0144	0.2037
AI987929	Ndrg1	Up	0.0058	0.1553
M35131	Nefh	Up	0.0145	0.2038
BC019524	Nek6	Up	0.0149	0.2045
BB103193	Nek8	Up	0.0332	0.2583
AK003786	Nfs1	Up	0.0268	0.2448
NM_010913	Nfya	Up	0.0243	0.2388
AK016744	Nmnat3	Up	0.0239	0.2386
AK005123	Nol11	Up	0.0394	0.2658
BF660256	Nop56	Up	0.0253	0.2425
NM_008728	Npr3	Up	0.0363	0.2628
AK008679	Nr4a2	Up	0.0305	0.2541
BI696369	Nrarp	Up	0.0181	0.2166
AW491344	Nrip2	Up	0.0366	0.2634
BB478558	Nsun3	Up	0.0078	0.1656
AB067530	Oas1h	Up	0.0024	0.1322
AW214584	Oaz2	Up	0.0132	0.1993
AK015312	Obfc2a	Up	0.0366	0.2634
BE956398	Ogfod1	Up	0.0104	0.1881
BB238604	Oip5	Up	0.0295	0.2516
AK019142	Ola1	Up	0.0041	0.1392
AF442824	Olfm3	Up	0.0452	0.2741
AI666798	OTTMUSG0000 0011097	Up	0.0159	0.2093
AK006346	Otub2	Up	0.0070	0.1628
AJ251462	P2rx4	Up	0.0241	0.2388
NM_033321	P2rx5	Up	0.0179	0.2166
AK021076	Parp6	Up	0.0338	0.2594
BI734351	Pcdhb14	Up	0.0107	0.1896
AF352788	Pcolce2	Up	0.0125	0.1951
BI107300	Pggt1b	Up	0.0341	0.2595
U73910	Phex	Up	0.0020	0.1311
NM_008840	Pik3cd	Up	0.0022	0.1311
NM_054097	Pip4k2c	Up	0.0204	0.2265
AV209062	Plac8l1	Up	0.0153	0.2061
NM_019788	Pldn	Up	0.0120	0.1951
BB277078	Plekha1	Up	0.0059	0.1553
BB706079	Plk4	Up	0.0204	0.2265
BI739353	Pmm1	Up	0.0044	0.1433
BB280835	Pofut1	Up	0.0008	0.0898
BC018194	Pofut2	Up	0.0064	0.1570
NM_021498	Pole3	Up	0.0010	0.0898
AF259673	Ppm1a	Up	0.0313	0.2546
NM_021400	Prg4	Up	0.0190	0.2199
AV024339	Prkacb	Up	0.0246	0.2400
NM_008856	Prkch	Up	0.0108	0.1901
NM_019991	Prl2a1	Up	0.0369	0.2637
AV209063	Prm1	Up	0.0479	0.2772
BE954519	Prrt3	Up	0.0007	0.0898
BB060144	Psd3	Up	0.0044	0.1430
AB029146	Psmd4	Up	0.0352	0.2602
BB034143	Psmd7	Up	0.0475	0.2757
BC016606	Psmg2	Up	0.0093	0.1767
NM_008965	Ptger4	Up	0.0242	0.2388
L08663	Ptpn11	Up	0.0006	0.0870
AW542340	Rab18	Up	0.0387	0.2645
NM_013862	Rabgap1l	Up	0.0322	0.2564
AK016099	Rabl3	Up	0.0131	0.1992
BC003738	Rad51ap1	Up	0.0178	0.2166
BC021784	Rad9b	Up	0.0060	0.1553
NM_011244	Rarg	Up	0.0282	0.2501
NM_009027	Rasgrf2	Up	0.0040	0.1383
BB537004	Rassf2	Up	0.0029	0.1330
BF011461	Rbbp4	Up	0.0295	0.2516

BB257313	Rgs13	Up	0.0356	0.2607
AF350047	Rgs3	Up	0.0050	0.1504
BF585144	Rgs5	Up	0.0055	0.1553
BB431052	Rlbp1l2	Up	0.0482	0.2780
NM_030743	Rnf114	Up	0.0133	0.1993
AA208652	Rps27	Up	0.0346	0.2602
NM_009100	Rptn	Up	0.0444	0.2731
AV297071	Rrp1	Up	0.0031	0.1330
BC008229	Rsrc2	Up	0.0016	0.1133
BB752007	Rtel1	Up	0.0096	0.1808
AJ428064	Rtp3	Up	0.0404	0.2680
BG060909	Scd2	Up	0.0234	0.2370
BM932452	Scoc	Up	0.0034	0.1330
BC021346	Scrn2	Up	0.0148	0.2045
BI788645	Sdc1	Up	0.0349	0.2602
AK017691	Sdccag8	Up	0.0172	0.2144
C76103	Sec63	Up	0.0152	0.2061
NM_009153	Sema3b	Up	0.0109	0.1902
BB535044	Sema3g	Up	0.0316	0.2562
AK009343	Serpina9	Up	0.0299	0.2537
AF426024	Serpinb1a	Up	0.0383	0.2645
NM_011111	Serpinb2	Up	0.0454	0.2741
NM_009126	Serpinb3a	Up	0.0114	0.1921
AV308638	Sesn2	Up	0.0410	0.2684
AK015776	Sf3a3	Up	0.0037	0.1349
NM_018780	Sfrp5	Up	0.0042	0.1404
AK008803	Sh2d4a	Up	0.0381	0.2645
BQ179335	Sh3bp5	Up	0.0210	0.2271
NM_009171	Shmt1	Up	0.0340	0.2595
AF226605	Sigmar1	Up	0.0176	0.2163
BC006711	Slc25a29	Up	0.0080	0.1662
M75135	Slc2a3	Up	0.0353	0.2602
BB105046	Slc38a3	Up	0.0148	0.2045
BC025870	Slu7	Up	0.0010	0.0898
C80870	Smg1	Up	0.0371	0.2643
BB821035	Smn1	Up	0.0130	0.1992
BF160651	Smyd5	Up	0.0227	0.2363
NM_033174	Snrpn	Up	0.0032	0.1330
C79000	Snw1	Up	0.0327	0.2564
NM_011435	Sod3	Up	0.0146	0.2044
AK008404	Sri	Up	0.0091	0.1767
BC024835	Ssrp1	Up	0.0359	0.2622
NM_138744	Ssx2ip	Up	0.0388	0.2645
AA289490	Stambp	Up	0.0015	0.1133
BB667844	Stbd1	Up	0.0046	0.1466
NM_019675	Stmn4	Up	0.0461	0.2749
BB213765	Susd5	Up	0.0158	0.2093
BB559861	Syt9	Up	0.0036	0.1349
BE862546	Tacc1	Up	0.0192	0.2207
NM_013763	Tbl2	Up	0.0016	0.1133
NM_011560	Tcte3	Up	0.0195	0.2221
BM205069	Thsd4	Up	0.0074	0.1636
NM_011587	Tie1	Up	0.0181	0.2166
BE373761	Tm4sf1	Up	0.0056	0.1553
AK014065	Tmed5	Up	0.0159	0.2093
AK013719	Tmem143	Up	0.0264	0.2440
NM_013749	Tnfrsf12a	Up	0.0365	0.2634
BB480482	Tnni3k	Up	0.0208	0.2265
BB142697	Tns4	Up	0.0001	0.0568
NM_022314	Tpm3	Up	0.0431	0.2729
BE380713	Tpm3	Up	0.0433	0.2729
NM_022314	Tpm3	Up	0.0293	0.2516
NM_026481	Tppp3	Up	0.0182	0.2166
U42405	Tpsab1	Up	0.0072	0.1635
NM_010781	Tpsb2	Up	0.0004	0.0844
BB825279	Trip11	Up	0.0320	0.2564
AF052492	Tspan7	Up	0.0044	0.1433
NM_133795	Ttc1	Up	0.0067	0.1608
AW492503	Ttc15	Up	0.0469	0.2752
NM_053273	Ttyh2	Up	0.0221	0.2323
NM_009446	Tuba3a	Up	0.0204	0.2265
BB284199	Txnrd1	Up	0.0072	0.1635
BC013688	Txnrd2	Up	0.0175	0.2161
NM_011670	Uchl1	Up	0.0052	0.1515
BC012697	Ucp2	Up	0.0353	0.2602
AF441835	Usp12	Up	0.0318	0.2564
BM235696	Usp34	Up	0.0173	0.2152
NM_030739	V1rd4	Up	0.0438	0.2731
NM_026776	Vps25	Up	0.0099	0.1855
NM_021387	Vstm2b	Up	0.0323	0.2564
AK010831	Wdyhv1	Up	0.0318	0.2564
W29605	Wnt7b	Up	0.0009	0.0898
BB280716	Zdhhc17	Up	0.0266	0.2443
AK020384	Zfp142	Up	0.0249	0.2412
NM_011980	Zfp146	Up	0.0410	0.2684
NM_019565	Zfp386	Up	0.0015	0.1126
AW413620	Zfp704	Up	0.0022	0.1311
BB126810	Zic4	Up	0.0074	0.1636

Table S7-B, Microarray Data: Genes identified by clustering analysis. ANOVA was conducted on microarray data without multiple hypothesis correction, yielding 855 genes that were differentially expressed among all groups at P < 0.01. These genes were subjected to hierarchical clustering, yielding a heatmap (Figure 10) showing separation of 6 clusters of differentially expressed genes identified as A-F. Genes in clusters A-F are given below.

**CLUSTER A**			**CLUSTER B**			**CLUSTER C**		
Gene Symbol	RefSeq Transcript ID	Marker ID	Gene Symbol	RefSeq Transcript ID	Marker ID	Gene Symbol	RefSeq Transcript ID	Marker ID
---	---	1438998_at	---	---	1446364_at	42980	NM_011889	1444778_at
---	---	1433000_at	---	---	1447523_at	---	---	1434025_at
---	---	1447239_at	---	---	1444863_at	---	---	1459047_x_at
---	---	1447698_x_at	---	---	1447390_at	---	---	1447081_at
---	---	1442451_at	---	---	1459134_at	---	---	1457732_at
---	---	1435119_at	---	---	1446382_at	---	---	1442977_at
---	---	1427659_at	---	---	1446875_at	---	---	1443018_at
1700028J19Rik	NR_029436 /// XM_901377 /// XM_924669	1432186_at	---	---	1441296_at	---	---	1447119_at
1700110I01Rik	XM_001471969	1453046_at	---	---	1458625_at	---	---	1444498_at
2310015D24Rik	XM_001474641	1431714_at	1700014N06Rik	NM_027067 /// XM_486774 /// XM_906752	1454199_at	---	---	1456122_at
2410042D21Rik	NM_024254	1426495_at	1700039M15Rik	---	1454548_at	---	---	1457326_at
2810405K02Rik	NM_025582	1423266_at	1700082M22Rik	---	1421639_at	---	---	1459352_at
4921525D07Rik	---	1430732_at	2700078F05Rik	---	1454464_at	---	---	1457822_at
4930533L02Rik	---	1432240_at	5830432F11Rik	---	1433346_at	---	---	1442936_at
4930550C17Rik	---	1432089_at	5830490A04Rik	---	1433121_at	---	---	1442092_at
4933406J10Rik	---	1431500_at	9130015A21Rik	XM_488633	1453953_at	---	---	1459069_at
4933425L06Rik	NM_025751	1449922_at	9230102K24Rik	NR_028438 /// XM_001480326 /// XM_001480584	1445056_at	---	---	1459936_at
Adal	NM_029475	1428198_at	Adamts1	NM_009621	1450716_at	---	---	1438881_at
Adap2	NM_172133	1425638_at	Alms1	NM_145223	1456950_at	---	---	1444309_at
Ak3	NM_021299	1423717_at	Alx3	NM_007441	1420555_at	---	---	1457998_at
Aph1b	NM_177583	1456500_at	Arid3a	NM_007880	1419396_at	---	---	1445716_at
BC017647	NM_145430	1424663_at	Atg12	NM_026217	1439457_x_at	---	---	1460149_at
C130078N14	---	1447140_at	Atp5sl	NM_025504	1430450_at	---	---	1459127_at
Cacng6	NM_133183	1425730_at	Bmp8a	NM_007558	1449873_at	---	---	1443042_at
Capza2	NM_007604	1423057_at	Bnip2	NM_001008238 /// NM_016787	1454442_at	---	---	1458437_at
Ccbp2	NM_021609	1422111_at	C77905	---	1459567_at	---	---	1447126_at
Cd200r3	NM_001128132 /// NM_001128133 /// NM_027578 /// NM_029018	1430645_at	Cabin1	NM_172549	1437794_at	---	---	1436398_at
Cd70	NM_011617	1449926_at	Cacna1a	NM_007578	1459996_at	---	---	1445498_at
Coq10a	NM_001081040	1458468_at	Cacna2d1	NM_001110843 /// NM_001110844 /// NM_001110845 /// NM_001110846 /// NM_009784	1449999_a_at	1110069O07Rik	---	1436786_at
Ctsa	NM_001038492 /// NM_008906	1458466_at	Camk2a	NM_009792 /// NM_177407	1457311_at	2310028O11Rik	NM_001163006 /// XM_901483 /// XM_924570	1436001_at
D2Ertd295e	---	1446133_at	Clec2e	NM_153506	1458415_at	2400003C14Rik	NM_028018	1423838_s_at
D6Wsu116e	NM_026585	1433589_at	D6Ertd90e	---	1459480_at	2610018G03Rik	NM_133729	1419033_at
Dclk1	NM_001111051 /// NM_001111052 /// NM_001111053 /// NM_019978	1436659_at	Etf1	NM_144866	1420024_s_at	2900093K20Rik	---	1440917_at
Dclk1	NM_001111051 /// NM_001111052 /// NM_001111053 /// NM_019978	1424271_at	Evi5	NM_007964	1417513_at	3110037C07Rik	---	1432905_at
Dnajc5	NM_016775	1448850_a_at	Fam133b	NM_001042501	1458926_at	3110043O21Rik	NM_001081343	1428468_at
Dpp3	NM_133803	1431594_at	Fam71f1	NM_207258	1437228_at	3110048L19Rik	NR_003549 /// XR_035185 /// XR_035371	1454402_at
Ece1	NM_199307	1434177_at	Fgf22	NM_023304	1460296_a_at	3110067C02Rik	---	1432796_at
Eps15	NM_001159964 /// NM_007943	1419251_at	Gm1821 /// Gm8441 /// Ubb	NM_011664 /// NR_002856 /// NR_002875 /// XR_032507 /// XR_033845	1449436_s_at	4922501C03Rik	NM_199316 /// XM_001000297 /// XM_001480975	1443899_at
Esr2	NM_010157 /// NM_207707	1426103_a_at	Gm4340	NM_001177535 /// XM_001480538 /// XM_001480542 /// XM_001480543	1443961_at	4930474H20Rik	XM_901924	1432058_at
Fam102b	NM_001163567 /// XM_283903 /// XM_974847	1455033_at	Gucy1a2	NM_001033322	1459336_at	4933401L05Rik	---	1432127_at
Fam110c	NM_027828	1418879_at	Ifitm7	NM_028968	1453917_at	6330503K22Rik	NM_182995	1455218_at
Fam110c	NM_027828	1449252_at	Kdm5b	NM_152895	1427143_at	9130221L21Rik	---	1440584_at
Gmfb	NM_022023	1448571_a_at	Kdm5d	NM_011419	1452563_a_at	9430085L16Rik	---	1454533_at
Gmfb	NM_022023	1448570_at	LOC100303645	NR_028425	1444292_at	9530006O14Rik	---	1433196_at
Gna13	NM_010303	1422556_at	LOC631639 /// Lonrf1	NM_001081150 /// XM_905519	1455665_at	Abcd4	NM_008992	1427829_at
Gpr137b /// Gpr137b-ps	NM_031999 /// NR_003568 /// XR_001658	1423164_at	Lsm12	NM_172947	1427998_at	Acat2	NM_009338	1435630_s_at
Gsdma	NM_021347	1457777_at	Mapre2	NM_001162941 /// NM_001162942 /// NM_153058	1442553_at	Adrm1	NM_019822	1438170_x_at
Heatr6	NM_145432	1424687_at	Mbnl1	NM_020007 /// XM_001474780	1416904_at	Ahsp	NM_133245	1449077_at
Ipmk	NM_027184	1436215_at	Nelf	NM_001039386 /// NM_001039387 /// NM_001177654 ///	1449474_a_at	Ajap1	NM_001099299 /// XM_001475023 /// XM_485506	1456197_x_at

Jph4	NM_001003829 /// NM_177049	1439031_at
Kif13a	NM_010617	1439532_s_at
Klf6	NM_011803	1427742_a_at
Klk4	NM_019928	1420763_at
Klrg2	NM_001033171	1430606_at
LOC100044703 /// March2	NM_145486 /// XR_030915	1424888_at
LOC100048130 /// Spata21	NM_177867 /// XR_034408	1455718_at
LOC636303	XR_033307	1445313_at
Lpgat1	NM_001134829 /// NM_172266	1424350_s_at
Mdga1	NM_001081160	1432062_at
Nfic	NM_008688 /// NM_026756	1427665_a_at
Nptn	NM_009145	1415821_at
Nrsn2	NM_001009948	1447717_x_at
Nus1	NM_030250	1434304_s_at
Oxsm	NM_027695	1419973_at
Pabpc2	NM_011033	1419500_at
Pcyt1a	NM_001163159 /// NM_001163160 /// NM_009981	1421957_a_at
Pcyt1a	NM_001163159 /// NM_001163160 /// NM_009981	1450434_s_at
Pdxdc1	NM_001039533 /// NM_053181	1428200_a_at
Pion	NM_175437	1440454_at
Plek	NM_019549	1417523_at
Plk4	NM_011495	1452115_a_at
Pppde1	NM_024282	1451621_at
Pyroxd2	NM_029011	1453491_at
Rap1b	NM_024457	1455349_at
Riok3	NM_024182	1460670_at
Rnd3	NM_028810	1416700_at
Rnf11	NM_013876	1426405_at
Saa3	NM_011315	1450826_a_at
Sesn3	NM_030261	1453313_at
Slc10a1	NM_001177561 /// NM_011387	1424898_at
Slc22a12	NM_009203	1422897_at
Slc25a26	NM_026255	1453886_a_at
Smc2	NM_008017	1429660_s_at
Tmem65	NM_175212	1452942_at
Trim30	NM_009099	1417961_a_at
Vamp3	NM_009498	1433916_at
Ywhaz	NM_011740	1416102_at
Zdhhc2	NM_178395	1452656_at

	NM_001177655 /// NM_020276	
Neurod2	NM_010895	1418995_at
Nipbl	NM_027707 /// NM_201232	1430309_at
Nol9	NM_001159599 /// NM_028727	1432219_at
Rasgrp1	NM_011246	1431749_a_at
Rfxank	NM_001025589 /// NM_011266	1425670_at
Serpinb12	NM_027971	1432173_at
Slc29a2	NM_007854	1448257_at
Slc6a1	NM_178703	1457183_at
Slurp1	NM_020519	1420562_at
Sypl2	NM_008596	1454371_at
Wnk1	NM_198703 /// NR_028257	1436746_at
Wnt16	NM_053116	1422941_at
Wnt5a	NM_009524	1448818_at
Ywhaz	NM_011740	1448219_a_at
Zranb1	NM_207302	1436228_at

AL023051	---	1444675_at
Anapc1	NM_008569	1415680_at
Ap2b1	NM_001035854 /// NM_027915	1458971_at
Armc1	NM_028840	1430472_at
Atp6ap2	NM_027439	1437688_x_at
Atp6ap2	NM_027439	1439456_x_at
Atpaf1	NM_181040	1436358_at
BC059050	---	1444497_at
BC100530 /// Stfa1	NM_001082543 /// NM_001082546	1435761_at
Bcl2l11	NM_009754 /// NM_207680 /// NM_207681	1456005_a_at
Bclaf1	NM_001025392 /// NM_001025393 /// NM_153787	1428844_a_at
Bend4	NM_001164806 /// XM_001477289	1456795_at
Brd2	NM_001025387 /// NM_010238 /// XM_001479277	1437210_a_at
Cacna1d	NM_001083616 /// NM_028981	1427974_s_at
Ccnd3	NM_001081635 /// NM_001081636 /// NM_007632	1438805_at
Ccnt1	NM_009833	1456477_at
Ccr9	NM_001166625 /// NM_009913	1427419_x_at
Cd96	NM_032465	1419226_at
Cdca7	NM_025866	1445681_at
Chchd7	NM_181391	1440158_x_at
Chfr	NM_172717	1433550_at
Chka	NM_001025566 /// NM_013490	1450264_a_at
Clip4	NM_030179 /// NM_175378	1437092_at
Cnot1	NM_153164 /// NM_178078 /// XM_894673 /// XM_914270	1434251_at
Coq5	NM_026504	1417264_at
Ctrb1	NM_025583	1448220_at
D030016E14Rik	NM_177240	1433752_s_at
D230025D16Rik	NM_145604	1452096_s_at
D630004K10Rik	---	1440840_at
D930026N18Rik	---	1435730_at
D930050J11	---	1457484_at
Dner	NM_152915	1456379_x_at
Dtl	NM_029766	1456652_at
Eif2ak1	NM_013557	1421901_at
Elavl4	NM_001038698 /// NM_001163397 /// NM_001163399 /// NM_010488	1452894_at
Fam126b	NM_172513	1456257_at
Fam63a	NM_133858	1438321_x_at
Fam63a	NM_133858	1437046_x_at
Fam83e	NM_001033170	1430717_at
Fancm	NM_178912	1447935_at
Fnip1	NM_173753	1434405_at
Galnt11	NM_144908	1432840_at
Gcn1l1	NM_172719	1433713_at
Gm10443 /// Rps28	NM_016844 /// XM_001478988 /// XM_001480106	1423763_x_at
Gm10471 /// Speer4b	NM_001177579 /// NM_028561 /// XR_030559	1429993_s_at
Gm13611 /// Gm4604 /// LOC100048508 /// Rpl36	NM_018730 /// XM_001480072 /// XM_001480721 /// XM_001480806 /// XM_001480807 /// XM_00	1433510_x_at
Gm13648	---	1443736_at
Gm2643 /// LOC100047186	XM_001474210 /// XM_001477923	1458656_at
Gpi1	NM_008155	1434814_x_at
H2afj	NM_177688 /// XM_906486	1424771_at
H3f3b	NM_008211	1430357_at
Hemgn	NM_053149	1418199_at
Hmgcs1	NM_145942	1433445_x_at
Hmgn1	NM_008251	1438940_x_at
Hnrnpa2b1	NM_016806 /// NM_182650	1433829_a_at
Hnrnpm	NM_001109913 /// NM_029804	1426698_a_at

Hnrnpu	NM_016805	1423050_s_at
Ift80	NM_026641	1427568_a_at
Ift81	NM_009879	1419286_s_at
Igdcc4	NM_020043	1416473_a_at
Kcnma1	NM_010610	1457018_at
Kif18b	NM_197959	1453226_at
Krt42	NM_212483	1439765_x_at
Lin28	NM_145833	1421749_at
LOC100046044 /// Nr2f1	NM_010151 /// XM_001475459	1418157_at
Mcm3ap	NM_019434	1448711_at
Mepce	NM_144913	1449747_at
Mphosph9	NM_001081323	1419909_at
Mpo	NM_010824	1415960_at
Mtf2	NM_013827	1418515_at
Napg	NM_028017	1443366_at
Ncbp1	NM_001033201	1433663_s_at
Ndufa2	NM_010885	1417368_s_at
Nebl	NM_028757	1439897_at
Nfyc	NM_001048168 /// NM_008692	1448963_at
Ngp	NM_008694	1418722_at
Nr2f2	NM_009697 /// NM_183261	1416159_at
Nrip1	NM_173440	1449089_at
Nrip1	NM_173440	1434384_at
Nrxn2	NM_020253	1435908_at
Numa1	NM_133947	1423974_at
Nup107	NM_134010	1426751_s_at
Odz3	NM_001145937 /// NM_011857	1447567_at
Paxip1	NM_018878	1423302_a_at
Paxip1	NM_018878	1434363_x_at
Phf20l1	NM_001081409	1437563_at
Phip	NM_001081216	1423432_at
Phip	NM_001081216	1440653_at
Pigo	NM_020035	1438155_x_at
Pigq	NM_011822	1437999_x_at
Pik3r2	NM_008841	1418463_at
Pknox1	NM_016670 /// NR_027493	1437470_at
Pnliprp1	NM_018874	1415777_at
Pold3	NM_133692	1426838_at
Ppp1cc	NM_013636	1450149_a_at
Prpf19	NM_134129	1456531_x_at
Qrsl1	NM_001081054	1456542_s_at
Rbbp7	NM_009031	1456227_x_at
Rbm25	NM_025930 /// NM_027349	1428907_at
Rbm39	NM_133242	1426671_a_at
Rev3l	NM_011264	1424632_a_at
Rhbdd3	NM_177370	1433760_a_at
Rnf2	NM_011277	1424873_at
Rnpc3	NM_001038696 /// NM_026043	1437461_s_at
Rps12	NM_011295	1433432_x_at
Rps6ka6	NM_025949	1429760_at
Rqcd1	NM_021383	1450887_at
Rsph3a /// Rsph3b	NM_001083945 /// NM_025789 /// XM_001475820 /// XM_001481177	1423119_at
Saps3	NM_001164159 /// NM_028999 /// NM_029456	1423881_at
Scrib	NM_134089	1427064_a_at
Sf3b1	NM_031179	1440818_s_at
Sf3b2	NM_030109	1433461_at
Sfrs1	NM_001078167 /// NM_173374	1428100_at
Sla2	NM_029983	1437504_at
Slc20a2	NM_011394	1434235_at
Slc39a9	NM_026244 /// XM_001472453 /// XM_891220	1452765_at
Slc47a1	NM_026183	1418395_at
Slc4a1	NM_011403	1416464_at
Slc4a1	NM_011403	1434502_x_at
Snhg8	NR_028574	1447896_s_at
Sox5	NM_001113559 /// NM_011444	1432190_at
Sp1	NM_013672	1418180_at
Strbp	NM_009261 /// NM_176932	1457218_at
Tmem170b	NM_001163572 /// XM_886379 /// XM_913065	1434556_at
Tmem183a	NM_001042485 /// NM_020588	1417199_at
Tnik	NM_001163007 /// NM_001163008 /// NM_001163009 /// NM_026910 /// XM_001473602 /// XM_00	1430579_at
Tnks2	NM_001163635 /// XM_001000101 /// XM_917270	1428388_at
Tomm7	NM_025394	1428214_at
Tpr	NM_133780	1426949_s_at
Tube1	NM_028006	1437942_x_at
Tubgcp3	NM_198031	1426707_at

Ubr1	NM_009461	1457796_at
Uck2	NM_030724	1439741_x_at
Usp24	NM_183225 /// XM_001473472 /// XM_001479517 /// XM_001481281 /// XM_915524	1448013_at
Usp34	XM_483996	1434392_at
Usp37	NM_176972	1434242_at
Usp7	NM_001003918	1419920_s_at
Vmn1r10 /// Vmn1r9	NM_053231 /// NM_134185	1450595_at
Vpreb1	NM_016982	1449869_at
Wdr33	NM_001170966 /// NM_001170967 /// NM_001170970 /// NM_028866	1418338_at
Wdr6	NM_031392	1455940_x_at
Whsc1	NM_001081102 /// NM_001177884 /// NM_175231	1435136_at
Ylpm1	NM_178363	1424532_at
Ywhaz	NM_011740	1436981_a_at
Ywhaz	NM_011740	1439005_x_at
Zfp292	NM_013889 /// XM_620009 /// XM_909747	1449515_at
Zfp523	NM_172617 /// XM_001478777	1434085_at
Zkscan17	NM_001130529 /// NM_172941	1434088_at

Cluster D

Gene Symbol	RefSeq Transcript ID	Marker ID
42979	NM_001159717 /// NM_001159718 /// NM_001159719 /// NM_010891	1423472_at
---	---	1434632_at
---	---	1439925_at
---	---	1440032_at
---	---	1445268_at
---	---	1442118_at
---	---	1444843_at
---	---	1443709_at
---	---	1459113_at
1110046J04Rik	XM_895551 /// XM_921831	1457779_at
2010110P09Rik	NM_027363	1431130_at
2310001A20Rik	NM_027977	1452159_at
2310051F07Rik	---	1435636_at
2610027L16Rik	NM_026403	1451039_at
2810002N01Rik	NM_001163388 /// NM_026511	1429248_at
2810410D24Rik	---	1456339_at
3110001D03Rik	NM_025849	1423317_at
3830406C13Rik	NM_146051 /// NM_178141	1436388_a_at
4933427G17Rik	XM_133717 /// XM_915104	1431904_at
5033417F24Rik /// Gm2816 /// LOC100045477	XM_001474374 /// XM_001474840	1431757_s_at
6330408A02Rik	NM_177312	1453086_at
6720456B07Rik	NM_133937	1423189_at
9030224M15Rik	NM_177793	1439689_at
9230104L09Rik	NM_029960	1432531_at
9430098F02Rik /// Ppp2ca	NM_019411	1439912_at
A830082K12Rik	---	1444007_at
Abi1	NM_001077190 /// NM_001077192 /// NM_001077193 /// NM_007380 /// NM_145994	1450890_a_at
Acaa1a /// Acaa1b	NM_130864 /// NM_146230	1416946_a_at
Acaa1a /// Acaa1b	NM_130864 /// NM_146230	1416947_s_at
Acaa2	NM_177470	1428146_s_at
Acp1	NM_001110239 /// NM_021330	1450720_at

Cluster E

Gene Symbol	RefSeq Transcript ID	Marker ID
1110038D17Rik	NM_175133	1428071_at
2010001K21Rik	XM_992633	1447576_at
Agap3	NM_139153	1423910_at
C030009O12Rik	---	1435095_at
Ccl27a	NM_001048179 /// NM_001164044 /// NM_001164045 /// NM_011336	1434962_x_at
Ccl27a	NM_001048179 /// NM_001164044 /// NM_001164045 /// NM_011336	1430375_a_at
Chtf8	NM_145412	1424028_at
Eno3	NM_001136062 /// NM_007933 /// XM_001471607	1417951_at
Evc	NM_021292	1448876_at
Gabrb1	NM_008069	1419719_at
Gm11602	---	1453354_at
Hirip3	NM_172746	1434936_at
Hoxa5	NM_010453	1448926_at
Hoxc4	NM_013553	1422870_at
Igsf3	NM_207205	1455048_at
Prpf6	NM_133701	1424036_at
Srpk1	NM_016795	1455107_at
Tekt2	NM_011902	1418484_at
Tspan14	NM_145928	1423924_s_at
Txnrd1	NM_001042513 /// NM_001042514 /// NM_001042523 /// NM_015762	1421529_a_at
Wdr6	NM_031392	1415770_at

Cluster F

Gene Symbol	RefSeq Transcript ID	Marker ID
---	---	1445393_at
Abcg1	NM_009593	1455221_at
C130022K22Rik	NM_172730	1456879_at
Cct3	NM_009836	1459987_s_at
Cfhr3	---	1432330_at
Cyb5rl	NM_175471	1427423_at
Dgcr2	NM_001109750 /// NM_010048	1421810_at
Dnttip1	NM_133763	1417666_at
Exoc4	NM_009148	1422686_s_at
Fam63b	NM_172772	1436841_at
Fbl	NM_007991	1416685_s_at
Fdft1	NM_010191	1448130_at
Il17rd	NM_134437	1429893_at
Limd1	NM_013860	1422731_at
LOC100046658	XM_001476568	1457109_x_at
Map2k1	NM_008927	1416351_at
Mapkapk5	NM_010765 /// XM_001479242	1417016_at
Ncbp2	NM_026554	1423045_at
Ncdn	NM_011986	1416853_at
Nfib	NM_001113209 /// NM_001113210 /// NM_008687	1427680_a_at
Pcbp4	NM_021567	1449054_a_at
Phka1	NM_008832 /// NM_173021	1422744_at
Phyhip	NM_145981	1455463_at
Prosc	NM_001039077 /// NM_001039078 /// NM_054057	1423044_at
Rars	NM_025936	1416312_at
Rhbdl3	NM_139228	1427579_at
Rnf103	NM_009543	1442414_at
Sun1	NM_024451	1426666_a_at
Thoc5	NM_172438	1452672_at
Tpbpa	NM_009411	1438190_x_at
Trub1	NM_028115 /// NM_029839	1428281_at

Adat3 /// Scamp4	NM_001100606 /// NM_019575	1450393_a_at
Adh5	NM_007410	1443380_at
AI324046	---	1427503_at
Aida	NM_181732	1423870_at
Ak2 /// LOC100047005	NM_001033966 /// NM_016895 /// XM_001477790	1448451_at
Akr1b3	NM_009658	1448319_at
Alg12	NM_001142357 /// NM_145477	1427558_s_at
Alox15	NM_009660	1420338_at
Anapc10	NM_026904	1429376_s_at
Anp32a	NM_009672	1421918_at
Anpep	NM_008486	1421424_a_at
Anxa2	NM_007585	1419091_a_at
Anxa3	NM_013470	1460330_at
Anxa7	NM_001110794 /// NM_009674	1416137_at
Apob48r	NM_138310	1420382_at
Arfgap3 /// LOC100045474	NM_025445 /// XR_032156	1426534_a_at
Arhgef12	NM_027144	1453853_a_at
Arhgef15 /// LOC100045735	NM_177566 /// XM_001474841	1455522_at
Arl2bp	NM_024191 /// NM_024269	1417112_at
Arl6ip5	NM_022992	1417225_at
Atp6v0a1	NM_016920	1425227_a_at
Bace1	NM_001145947 /// NM_011792	1450384_at
BC015286	NM_198171	1457619_at
BC024814	NM_146247	1424434_at
Bcl7b	NM_009745 /// XM_001474834 /// XM_001480220	1448247_at
Bdnf	NM_001048139 /// NM_001048141 /// NM_001048142 /// NM_007540	1422168_a_at
Bfar	NM_001177552 /// NM_025976	1426488_at
Bicd2	NM_001039179 /// NM_001039180 /// NM_029791	1422745_at
Bmyc	NM_023326	1428669_at
Brf2	NM_025686	1429561_at
Brms1	NM_134155	1417897_at
Btk	NM_013482	1422755_at
C1galt1	NM_052993	1422772_at
C1qtnf1	NM_019959	1448912_at
C1qtnf7	NM_001135172 /// NM_175425	1429030_at
C1qtnf9	NM_183175	1442033_at
C230052I12Rik	NM_178643	1454779_s_at
Cand1	NM_027994 /// XM_125901 /// XM_904108	1428648_at
Cast	NM_009817	1451413_at
Ccbp2	NM_021609	1437937_at
Ccdc159	NM_001164614 /// NM_025977	1420430_a_at
Ccng1	NM_009831	1450016_at
Ccrl2	NM_017466	1427736_a_at
Cd151	NM_001111049 /// NM_001111050 /// NM_009842	1420921_at
Cd37	NM_007645	1419206_at
Cd55	NM_010016	1418762_at
Cd55	NM_010016	1460242_at
Cd59a	NM_001111060 /// NM_007652	1418710_at
Cd5l	NM_009690	1449193_at
Cd79a /// LOC100047815	NM_007655 /// XM_001478920	1418830_at
Cd99l2	NM_138309	1456747_x_at
Cdc27	NM_145436	1426076_at
Cdc42	NM_009861	1415724_a_at
Cdc42ep5	NM_021454	1418712_at
Cdkn3	NM_028222 /// XM_899867 /// XM_919022	1430574_at
Cdyl2	NM_027305 /// NM_029441	1432229_a_at
Chst2	NM_018763 /// XM_001471695	1422758_at
Cinp	NM_026048	1425830_a_at
Clec4d	NM_001163161 /// NM_010819	1420804_s_at
Clip1	NM_019765	1450351_a_at
Cltb	NM_028870	1453063_at
Cltb /// LOC100046457	NM_028870 /// XR_032589	1460740_at
Col4a3bp	NM_001164222 /// NM_023420	1449847_a_at
Coro1c	NM_011779	1449660_s_at
Cox19	NM_197980	1434923_at
Cpne8	NM_001033851 /// NM_025815	1430520_at
Cr1l	NM_013499	1422563_at
Creld1	NM_133930	1416537_at
Csgalnact1	NM_172753	1452365_at

Csnk1e	NM_013767	1417176_at
Ctnnal1	NM_018761	1420930_s_at
Ctnnbl1	NM_025680	1448582_at
Cttnbp2nl	NM_001163332 /// NM_001163333 /// NM_030249	1449300_at
Cxcr7	NM_007722	1417625_s_at
Cyb5b	NM_025558	1448844_at
Cyb5b	NM_025558	1417767_at
Cyb5r3	NM_029787	1425329_a_at
Cybb	NM_007807	1436779_at
Cyp4v3	NM_133969	1417070_at
Cysltr1	NM_021476	1418944_at
D630032N06Rik	NR_028329 /// XR_035235 /// XR_035464	1441641_at
Dbi	NM_001037999 /// NM_007830	1422432_at
Dda1	NM_025600 /// XM_001476732 /// XM_912953	1429039_s_at
Ddb1	NM_015735	1415735_at
Ddx54	NM_028041	1460396_at
Dedd	NM_001128609 /// NM_011615	1432000_a_at
Depdc6	NM_001037937 /// NM_145470	1451348_at
Dirc2	NM_153550	1454654_at
Dnajc21	NM_030046 /// XM_001473469 /// XM_001473643	1420220_x_at
Dnajc24	NM_026992	1430718_s_at
Dnm2	NM_001039520	1432005_at
Drg2	NM_021354	1417692_at
E2f1	NM_007891	1431875_a_at
Ecsit	NM_012029	1417080_a_at
Edem2	NM_145537	1423695_at
Efhd1	NM_028889	1448507_at
Egln1	NM_053207	1451110_at
Ehd4	NM_133838	1449852_a_at
Elk3	NM_013508 /// NM_205536	1448797_at
Endod1	NM_028013	1426540_at
Eral1	NM_022313	1420519_a_at
Erg	NM_133659	1440244_at
Evi2a	NM_001033711 /// NM_010161	1450241_a_at
Exoc6	NM_175353	1424231_s_at
F10	NM_007972	1449305_at
Fabp5	NM_010634	1416022_at
Fabp7	NM_021272	1450779_at
Fads3	NM_021890	1449219_at
Fads3	NM_021890	1435910_at
Fah	NM_010176	1417220_at
Fam101b	NM_029658 /// XM_893392 /// XM_983541	1429764_at
Fam101b	NM_029658 /// XM_893392 /// XM_983541	1456603_at
Fam114a1	NM_026667	1448648_at
Fam114a2	NM_001168667 /// NM_001168668 /// NM_026342	1451283_at
Fam120a	NM_001033268	1439120_at
Fam122a	NM_026520	1430769_s_at
Fam158a	NM_033146	1418474_at
Fam47a	NM_001164739 /// XM_135940 /// XM_980994	1431445_at
Fam82a1	NM_201361	1455063_at
Fam96b	NM_026753	1428874_at
Fbxl7	NM_176959	1456220_at
Fbxo43	NM_001081253	1427415_at
Fbxw13	NM_177598	1443298_at
Fbxw5	NM_013908	1417476_at
Fcgr2b	NM_001077189 /// NM_010187	1451941_a_at
Fcgr2b	NM_001077189 /// NM_010187	1455332_x_at
Fdx1l /// Zglp1	NM_001039824 /// NM_001103168 /// XM_001475156 /// XM_001475715	1430293_a_at
Fez2	NM_199448	1434348_at
Fgf2	NM_008006	1449826_a_at
Flt1	NM_010228	1419300_at
Flt1	NM_010228	1440926_at
Fmnl3	NM_011711	1426825_at
Fnta /// LOC100046996	NM_008033 /// XM_001477383	1417465_at
Frg1	NM_013522	1417253_at
Fscn1	NM_007984	1416515_at
Ftsjd2	NM_028791	1451082_at
Gch1	NM_008102	1420499_at
Gimap6	NM_153175	1427891_at
Glt25d1	NM_146211	1433496_at
Gltp	NM_019821	1419027_s_at
Gm10345	XM_001476072	1428995_at
Gm10575	---	1440507_at
Gm4627	XM_001479807 /// XM_001480898	1458844_at

Gm5802	XR_033737 /// XR_034695	1455890_x_at
Gm8154	XM_984447	1446637_at
Gmpr2	NM_177992	1416356_at
Gna11	NM_010301	1449144_at
Gnai1	NM_010305	1427510_at
Gng2	NM_001038637 /// NM_010315	1428157_at
Gorasp1	NM_028976	1451671_at
Gpm6b	NM_001177955 /// NM_001177956 /// NM_001177957 /// NM_001177958 /// NM_001177959 /// NM	1425942_a_at
Gpr137b /// LOC100044979	NM_031999 /// XM_001473441	1449670_x_at
Gpr172b	NM_029643	1456278_x_at
Gpx7	NM_024198	1417836_at
Grb2	NM_008163	1449111_a_at
Gstm1	NM_010358	1425627_x_at
H2-gs10 /// H2-Q6 /// LOC68395	NM_001143689 /// NM_207648 /// XM_903697 /// XR_032175 /// XR_032223 /// XR_034205	1451644_a_at
Hapln3	NM_178255	1431152_at
Haus1	NM_146089	1424955_at
Haus2	NM_025475	1430577_at
Hey1	NM_010423	1415999_at
Higd2a	NM_025933	1418002_at
Hivep2	NM_010437	1422018_at
Hoxc6	NM_010465	1427361_at
Hsbp1	NM_024219	1423906_at
Hspa4	NM_008300	1416147_at
Hspa4l	NM_011020	1418253_a_at
Ifi27l1	NM_026790 /// NM_194066 /// NM_194067 /// NM_194068 /// NM_194069	1452956_a_at
Ifnar2	NM_001110498 /// NM_010509	1451462_a_at
Ikbkg	NM_001136067 /// NM_001161421 /// NM_001161422 /// NM_001161423 /// NM_001161424 /// NM	1421209_s_at
Ikbkg	NM_001136067 /// NM_001161421 /// NM_001161422 /// NM_001161423 /// NM_001161424 /// NM	1435647_at
Il10ra	NM_008348	1448731_at
Il10rb	NM_008349	1419455_at
Il17re	NM_001034029 /// NM_001034031 /// NM_145826	1452109_at
Il34	NM_001135100 /// NM_029646	1431920_a_at
Impad1	NM_177730	1437290_at
Islr2	NM_001161535 /// NM_001161536 /// NM_001161537 /// NM_001161538 /// NM_001161539 /// NM	1434421_at
Itln1	NM_010584	1418165_at
Jagn1	NM_026365	1417064_at
Junb	NM_008416	1415899_at
Kank2	NM_145611	1425991_a_at
Katnal1	NM_153572	1439606_at
Kcnk3	NM_010608	1426058_a_at
Kctd10	NM_001159941 /// NM_026145	1423694_at
Kctd20	NM_025888	1416323_at
Klf13	NM_021366	1432543_a_at
Klf2	NM_008452	1448890_at
Klf4	NM_010637	1417394_at
Klhl8	NM_178741	1433526_at
l7Rn6	NM_026304	1419351_a_at
Laptm4b	NM_033521	1416148_at
Lasp1	NM_010688	1448207_at
Lats2	NM_015771 /// NM_153382	1425420_s_at
Lhfp	NM_175386	1433776_at
Lin37	NM_029377	1426577_a_at
Lin7c	NM_011699	1418898_at
Lman1	NM_001172062 /// NM_027400	1428129_at
Lmbr1	NM_020295	1426273_at
LOC100045106 /// Vstm2b	NM_021387 /// XM_001473683	1419118_at
LOC100045146 /// Ranbp9	NM_019930 /// XM_001473754	1422736_at
LOC100046464 /// Mfng	NM_008595 /// XR_032638	1416992_at
LOC100046704 /// Nras	NM_010937 /// XM_001476989	1422688_a_at

LOC100046746 /// Smap1	NM_028534 /// XM_001476743	1432029_a_at
LOC634379 /// Nkd1	NM_001163660 /// NM_027280 /// XM_909079	1417278_a_at
Lrrc57	NM_001159609 /// NM_001159610 /// NM_001159612 /// NM_025657 /// NR_027510	1430057_s_at
Lrrc57	NM_001159609 /// NM_001159610 /// NM_001159612 /// NM_025657 /// NR_027510	1428312_at
Lysmd1	NM_028134 /// NM_153121	1460438_at
Lyz1	NM_013590	1436996_x_at
Lyz1	NM_013590	1439426_x_at
Lyz1	NM_013590	1450822_at
Lyz2	NM_017372	1423547_at
M6pr	NM_010749	1416385_a_at
Maoa	NM_173740	1442676_at
Mapre1	NM_007896	1422764_at
Mapre2	NM_001162941 /// NM_001162942 /// NM_153058	1426244_at
Mblac2	NM_028372	1437704_at
Mcpt4	NM_010779	1425967_a_at
Me3	NM_181407	1429071_at
Med14	NM_001048208 /// NM_012005	1450229_at
Mfsd8	NM_028140	1452335_at
Mitd1	NM_026913	1426133_a_at
Mkrn2	NM_023290	1448897_at
Mlec	NM_175403	1436508_at
Mmrn2	NM_153127	1439518_at
Mnat1	NM_008612	1432177_a_at
Mrgprf	NM_145379	1425894_at
Mrm1	NM_145433	1424219_at
Mrpl4	NM_023167	1416510_at
Mrpl42	NM_026065	1418439_at
Mtap	NM_024433	1451346_at
Mvk	NM_023556	1418052_at
Mxd4	NM_010753	1422574_at
Myct1	NM_026793	1452072_at
Myct1	NM_026793	1438048_at
Nacc2	NM_001037098 /// NM_026495	1429582_at
Nagk	NM_001164187 /// NM_019542	1419509_a_at
Nap1l1	NM_001146707 /// NM_015781	1420478_at
Ncam1	NM_001081445 /// NM_001113204 /// NM_010875	1421966_at
Ncln	NM_134009	1424203_at
Nde1	NM_001114085 /// NM_023317	1435737_a_at
Ndfip2	NM_029561	1452066_a_at
Ndufa4l2	NM_001098789 /// XM_001004705 /// XM_979012	1434905_at
Nedd1	NM_008682	1423196_at
Nfya	NM_001110832 /// NM_010913	1422082_a_at
Nhsl2	NM_001163610 /// XM_001478287 /// XM_896183 /// XM_919948 /// XR_031588	1455040_s_at
Nipa1	NM_153578	1434864_at
Nln	NM_029447	1424981_at
Npc2	NM_023409	1416901_at
Nras	NM_010937	1454060_a_at
Nudt13	NM_026341	1454075_s_at
Nup50	NM_016714	1450722_at
Nupl1	NM_170591	1429878_a_at
Olfm1	NM_001038612 /// NM_001038613 /// NM_001038614 /// NM_019498	1426562_a_at
Orm2	NM_011016	1420438_at
P2rx5	NM_033321	1449433_at
P2rx7	NM_001038839 /// NM_001038845 /// NM_001038887 /// NM_011027	1419853_a_at
Palm	NM_001161747 /// NM_023128	1422034_a_at
Pamr1	NM_173749	1433529_at
Paqr7	NM_027995	1460674_at
Pard3	NM_001013580 /// NM_001013581 /// NM_001122850 /// NM_033620	1436765_at
Parva	NM_020606	1431375_s_at
Pde2a	NM_001008548 /// NM_001143848 /// NM_001143849 /// NR_026574	1452202_at
Pdgfb	NM_011057	1450413_at

Pdrg1	NM_178939	1428308_at
Pdrg1	NM_178939	1428309_s_at
Pdzd11	NM_028303	1451269_at
Pea15a	NM_011063	1416407_at
Phactr2	NM_001033257 /// XM_001473029 /// XM_001473842	1452404_at
Pias3	NM_001165949 /// NM_018812 /// NM_146135	1421646_a_at
Pigc	NM_001039045 /// NM_026078	1424190_at
Plcb4	NM_013829	1425339_at
Plekhg1	NM_001033253 /// NM_001159942	1440991_at
Pno1	NM_025443	1450735_at
Poc5	NM_026173	1419265_at
Podxl	NM_013723	1448688_at
Pold4	NM_027196	1427885_at
Pole3	NM_021498	1452743_at
Polr3c	NM_028925 /// XM_131049 /// XM_908475	1451658_a_at
Pot1a	NM_133931	1456369_at
Ppp1r7	NM_023200	1417919_at
Prdm9	NM_144809 /// XM_619431 /// XM_911812	1426107_at
Prdx4	NM_016764	1416167_at
Prg4	NM_001110146 /// NM_021400 /// XM_355243	1449824_at
Prkch	NM_008856	1422079_at
Prkra	NM_011871	1447737_at
Prrx1	NM_001025570 /// NM_011127 /// NM_175686	1425526_a_at
Pter	NM_008961	1417667_a_at
Ptpmt1	NM_025576	1426581_at
Pvrl2	NM_001159724 /// NM_008990	1424456_at
Rab3il1	NM_144538	1425133_s_at
Rac1	NM_009007	1451086_s_at
Rai12	NM_018740 /// NR_027884 /// NR_027977	1431411_a_at
Rap1gds1	NM_001040690 /// NM_145544	1425266_a_at
Rasgrp3	NM_001166493 /// NM_207246	1438030_at
Rasip1	NM_028544	1428016_a_at
Rassf3	NM_138956	1448546_at
Rbm42	NM_133693	1454183_at
Rcan1	NM_001081549 /// NM_019466	1416600_a_at
Rcan2	NM_030598 /// NM_207649	1450243_a_at
Rcan3	NM_022980	1434027_at
Rcbtb2	NM_001170694 /// NM_134083 /// NR_033185	1416389_a_at
Rftn1	NM_181397	1428696_at
Rnasek	NM_173742	1426745_at
Rnf214	NM_178709	1427274_at
Rps6kc1	NM_178775	1434563_at
Rpusd2	NM_173450	1429442_at
Rras2	NM_025846	1417398_at
Rtn1	NM_001007596 /// NM_153457	1429761_at
Ryk	NM_001042607 /// NM_013649	1426388_s_at
S100a16	NM_026416	1425560_a_at
S100a3	NM_011310	1421856_at
S1pr1	NM_007901	1423571_at
Samd4	NM_001037221 /// NM_001163433 /// NM_028966	1429449_at
Sap18	NM_009119	1419443_at
Sbds	NM_023248	1426480_at
Scrn3	NM_029022	1436573_at
Scyl2	NM_198021	1440269_at
Sdc1	NM_011519	1415943_at
Sdccag3	NM_001085407 /// NM_001085408 /// NM_026563	1453195_at
Sema3c	NM_013657	1420696_at
Senp2	NM_029457 /// NR_027488	1451717_s_at
Serpinb1a	NM_025429	1416318_at
Serpinb2	NM_001174170 /// NM_011111	1419082_at
Serpinb9	NM_009256	1439790_at
Setd3	NM_028262	1451748_a_at
Sh3kbp1	NM_001135727 /// NM_001135728 /// NM_021389	1431592_a_at
Siae	NM_011734	1416538_at
Sike1	NM_025679	1450679_at
Slc11a1	NM_013612	1420361_at
Slc22a2	NM_013667	1419117_at
Slc22a4	NM_019687	1417639_at
Slc24a3	NM_053195	1424308_at

Slc25a19	NM_026071	1424316_at
Slc41a3	NM_001037493 /// NM_027868	1425439_a_at
Slc48a1	NM_026353	1450410_a_at
Slfn1	NM_011407	1447614_at
Slfn5	NM_183201	1456288_at
Smo	NM_176996	1427048_at
Smoc2	NM_022315	1431362_a_at
Snap23	NM_001177792 /// NM_001177793 /// NM_009222	1420897_at
Snrnp25	NM_030093	1428004_at
Snx3	NM_017472	1422480_at
Sod2	NM_013671	1417193_at
Sparc	NM_009242	1448392_at
Spata6	NM_026470	1418650_at
Spg20	NM_001144987 /// NM_001144988 /// NM_144895	1424876_s_at
Spire1	NM_176832 /// NM_194355	1454962_at
Srd5a3	NM_020611	1423574_s_at
Sri	NM_001080974 /// NM_025618	1450878_at
Sri	NM_001080974 /// NM_025618	1423155_at
Srp72	NM_025691	1428876_at
St3gal3	NM_001161774 /// NM_009176	1450406_a_at
Stom	NM_013515	1419099_x_at
Stra13	NM_016665	1416553_at
Stx17	NM_026343	1427924_at
Surf2	NM_013678	1433609_s_at
Suv420h1	NM_001167884 /// NM_001167885 /// NM_001167886 /// NM_001167887 /// NM_001167888 /// NM	1451817_at
Synj2bp	NM_025292	1448853_at
Tada3	NM_133932	1417467_a_at
Tbcd	NM_029878	1452194_at
Tek	NM_013690	1418788_at
Tgfb1	NM_011577	1420653_at
Thop1	NM_022653	1448907_at
Timd4	NM_178759	1455318_at
Tlr4	NM_021297	1442827_at
Tm4sf1	NM_008536	1450958_at
Tmcc3	NM_001168684 /// NM_172051 /// NM_177026	1434252_at
Tmem106a	NM_144830	1425025_at
Tmem106b	NM_027992	1435075_at
Tmem109	NM_134142	1416033_at
Tmem14a	NM_029398	1428447_at
Tmem160	NM_026938	1460424_at
Tmem206	NM_025864	1417030_at
Tmem209	NM_178625	1452199_at
Tmem38b	NM_028053	1424700_at
Tmem41a	NM_025693	1424191_a_at
Tmem85	NM_026519	1418124_at
Tmem88	NM_025915	1453285_at
Tmem98	NM_029537	1424133_at
Tnfrsf10b	NM_020275	1422344_s_at
Tom1l1	NM_028011	1451117_a_at
Top1mt	NM_028404	1460370_at
Tpd52l2	NM_025482	1417107_at
Tppp3	NM_026481	1416713_at
Tpsab1	NM_031187 /// NR_033521	1426175_a_at
Tpsb2	NM_010781	1422180_a_at
Tradd	NM_001033161	1429117_at
Trak2	NM_172406	1435016_at
Trappc4	NM_021789	1415674_a_at
Trim34	NM_030684	1426092_a_at
Tspan18	NM_183180 /// XM_001478824 /// XM_001479670	1437095_at
Tspan7	NM_019634	1417502_at
Ttll1	NM_178869	1426427_at
Tubd1	NM_019756	1421748_a_at
Txndc12	NM_025334	1415738_at
Txndc17	NM_026559	1423035_s_at
Txndc5	NM_145367	1451091_at
Uaca	NM_028283	1452985_at
Ube2d3	NM_025356	1423113_a_at
Ube2l3	NM_009456	1417907_at
Ube2n	NM_080560	1422559_at
Ugt1a1 /// Ugt1a10 /// Ugt1a2 /// Ugt1a5 /// Ugt1a6a /// Ugt1a6b /// Ugt1a7c /// Ugt1a9	NM_013701 /// NM_145079 /// NM_201410 /// NM_201641 /// NM_201642 /// NM_201643 /// NM_	1426261_s_at
Unc119	NM_011676	1418123_at
Unc45b	NM_178680	1436939_at
Urm1	NM_026615	1437227_at
Vars	NM_011690	1448472_at
Vgll4	NM_177683	1455992_at

Vldlr	NM_001161420 /// NM_013703	1451156_s_at		
Vwa5a	NM_001145957 /// NM_172767	1426221_at		
Wdr18	NM_175450	1433635_at		
Wdr26	NM_145514	1451188_at		
Wdr33	NM_001170966 /// NM_001170967 /// NM_001170970 /// NM_028866	1453554_a_at		
Yif1b	NM_001110201 /// NM_029887	1428445_at		
Zbtb3	NM_001098237 /// NM_133759	1427106_at		
Zc3h12a	NM_153159	1443993_at		
Zdhhc2	NM_178395	1453481_at		
Zdhhc2	NM_178395	1452655_at		
Zfand2a	NM_001159908 /// NM_133349	1415940_at		
Zfp319	NM_024467	1439008_at		
Zfp664	NM_001081750	1434063_at		
